# Real-time prediction of bladder urine leakage using fuzzy inference system and dual Kalman filtering in cats

**DOI:** 10.1038/s41598-024-53629-5

**Published:** 2024-02-16

**Authors:** Amirhossein Qasemi, Alireza Aminian, Abbas Erfanian

**Affiliations:** Department of Biomedical Engineering, School of Electrical Engineering, Iran Neural Technology Research Center, Iran University of Science and Technology (IUST), Tehran, Iran

**Keywords:** Biomedical engineering, Signal processing

## Abstract

The use of electrical stimulation devices to manage bladder incontinence relies on the application of continuous inhibitory stimulation. However, continuous stimulation can result in tissue fatigue and increased delivered charge. Here, we employ a real-time algorithm to provide a short-time prediction of urine leakage using the high-resolution power spectrum of the bladder pressure during the presence of non-voiding contractions (NVC) in normal and overactive bladder (OAB) cats. The proposed method is threshold-free and does not require pre-training. The analysis revealed that there is a significant difference between voiding contraction (VC) and NVC pressures as well as band powers (0.5–5 Hz) during both normal and OAB conditions. Also, most of the first leakage points occurred after the maximum VC pressure, while all of them were observed subsequent to the maximum VC spectral power. Kalman-Fuzzy method predicted urine leakage on average 2.2 s and 1.6 s before its occurrence and an average of 2.0 s and 1.1 s after the contraction started with success rates of 94.2% and 100% in normal and OAB cats, respectively. This work presents a promising approach for developing a neuroprosthesis device, with on-demand stimulation to control bladder incontinence.

## Introduction

Two primary bladder functions, urine storage and micturition, are coordinated by the lower urinary tract (LUT) neural activity. Neurologic lesions (e.g. spinal cord injury, multiple sclerosis, Parkinson’s disease, and others) can affect the LUT functions^1^. A syndrome that is associated with the lower urinary tract dysfunction is overactive bladder (OAB), which is defined as “urinary urgency, with or without urge incontinence, usually with frequency and nocturia” by the International Continence Society (ICS)^2,3^.

Sacral neuromodulation (SNM) is an established electrical stimulation system for treatment of OAB^4–8^. In addition to SNM, electrical stimulation of other nerve locations has shown promising results for the management of patients with OAB and preventing urinary incontinence^4–8^. Several studies have shown that electrical stimulation applied to the pudendal nerve^9–13^, sacral dorsal root ganglion (DRG)^14,15^, and pelvic nerve^16,17^, at certain frequencies can inhibit bladder hyperreflexia, maintain continence, and increase the bladder capacity. In these studies, the inhibitory pathways were stimulated continuously, and habituation in response to continuous stimulation may affect the effectiveness of bladder inhibition^18,19^, as has occurred for sympathetic skin response during repeated electrical stimulation^20^.

To overcome the problem of continuous stimulation, a conditional stimulation scheme has been proposed, in which the stimulation is triggered at the onset of an involuntary bladder contraction^21^. Conditional stimulation has the potential to reduce the habituation of inhibitory reflexes^22^ and prevent loss of continence. This enables the bladder to store more urine before an uncontrollable contraction occurs.

A critical issue in conditional stimulation is the requirement of a signal to determine the onset of a hyper-reflexive bladder contraction. The pudendal nerve trunk (PNT) electroneurogram (ENG) has been used to detect the onset of hyper-reflexive bladder contraction in alpha-chloralose anesthetized cats during isovolumetric condition using the cumulative sum (CUSUM) algorithm^23^. It was demonstrated that the CUSUM algorithm performed better than either a constant threshold or a dynamic threshold algorithm in detecting hyper-reflexive bladder contraction. Also, it was demonstrated that conditional stimulation controlled by PNT ENG can increase the bladder capacity compared to continuous stimulation^20^. Furthermore, it has been shown that the afferent sacral root nerve activity (S1 nerve root) can be utilized to detect hyperreflexia-like bladder contractions using the CUSUM algorithm^24^.

Monitoring the Electromyography (EMG) of the external anal sphincter (EAS) and external urethral sphincter (EUS) has been also proposed for detecting bladder contraction^25–27^. It was shown that the onset of bladder contractions can be detected by CUSUM analysis of the EAS EMG in both synergic and dyssynergic cats as well as humans^25^. The EUS EMG has also been proposed for the detection of bladder contractions in patients with both neurogenic detrusor overactivity (NDO) and detrusor sphincter dyssynergia (DSD)^26,27^. The detection method is based on the kurtosis-based scaling function of the root mean square (RMS) of EMG over a sliding window. Kurtosis is a measure of the tailedness of a distribution and it is assumed that undesired bladder contractions occur during a single motor unit contraction, resulting in a high value of kurtosis. On the other hand, a strong continuous bladder contraction leads to a low value of kurtosis.

Several studies have demonstrated the utilization of the measured or estimated bladder pressure to detect the onset of bladder contraction^28–35^. Crossing the bladder pressure from a predefined threshold (e.g. 10 cmH_2_O)^28^ and monotonic increasing the estimated bladder pressure from a predefined pressure threshold for a period of time^33,34^ have been proposed as the stimulation trigger in a closed-loop control. However, bladder pressure may be susceptible to influence from various artifacts, such as bladder non-voiding contraction (NVC) caused by exercise, coughing, laughing, and sneezing. To cope with this problem, wavelet decomposition of the measured bladder pressure was used to decompose the bladder contraction with voiding contraction (VC) from NVC^31^. It was assumed that the detail component at the fifth level corresponds to the artifact and the coarse component to the bladder contraction.

Streng et al.^29^ investigated the pattern changes of the bladder pressure during filling the bladder in rats. It was demonstrated that the amplitude and frequency of the phasic activity changed during the filling phase. In the early part of the filling, no oscillation could be observed (phase 0). The phase 0 was followed by a period with slow oscillation (with frequency below 0.1 Hz). Then, this phase was followed by a period of small, fast oscillation that superimposed on the slow oscillation with a larger amplitude (phase II). In the final phase (III), immediately before micturition, the frequency of the oscillations decreased, and the amplitude increased. Due to these changes in the frequency pattern of the bladder pressure prior to voiding, some studies have developed methods to detect and predict the onset of bladder voiding contraction^30,32,35^. Clavica et al.^30^ developed a method for prediction of voiding contraction in intact and OAB anesthetized rats based on the power of the bladder pressure in the band of 0.2–0.6 Hz. The mean power of the band of 0.2–0.6 Hz during 100 s before the start of contraction was considered as the threshold. Then, the power of each 10 s window of the bladder pressure in the band of 0.2–0.6 Hz was compared to the defined threshold for prediction. The real-time of this algorithm was implemented with dorsal penile nerve (DPN) stimulation in an anesthetized rat^32^.

Recently, a method based on machine learning was proposed for the prediction of voiding contraction using the band-power of the measured bladder pressure in anesthetized rats^35^. A linear discriminant analysis (LDA) classifier using the band-power of the measured bladder pressure as the feature inputs was able to predict voiding contractions by more than 25 s in advance. But this method needs a training phase. Also, predicting VCs 25 s in advance requires long-lasting stimulation time, which may cause tissue fatigue or increase battery power consumption.

All previous studies have primarily focused on detecting the onset of VCs, defined as either an increase in baseline pressure preceding a VC or the appearance of high-frequency oscillations in a rat's bladder pressure. The primary purpose of detecting the onset of contractions is to utilize it as the stimulation trigger signal for incontinence control, aiming to prevent urine leakage. However, in the case of the neurogenic bladder with detrusor overactivity resulting from suprasacral spinal cord injury, high pressure NVCs (i.e., non-phasic contractions) occur that may or may not precede a voiding contraction^36,37^.

The major contribution of the current study is to predict short-time urine leakage from intravesical pressure. For this purpose, a real-time algorithm based on Kalman filtering and fuzzy logic is developed to predict urine leakage in alpha-chloralose anesthetized cats during normal and OAB conditions.

## Methods

All animal care and surgical procedures were approved by the Animal Care and Ethics Committee of the Iran Neural Technology Research Centre, Iran University of Science and Technology. All protocols and methods were performed according to the recommendations and relevant guidelines for the care and use of laboratory animals and written informed consent was obtained from all pet owners upon recruitment. Moreover, the study was carried out in compliance with the ARRIVE guidelines.

### Surgical preparation and setup equipment

Twelve sexually intact adult domestic short-hair male cats (7–19 month, 1.9–5.8 kg, median: 2.9 kg) were used in this study. Animals with irregular voiding period, feline herpesvirus, calicivirus, chlamydia, toxoplasmosis, and low weight compared to their body structure were excluded from the experiment before surgery. In two cats, the catheter was blocked and fluid infusion into the bladder was interrupted. Two cats were also excluded due to a non-reflexive bladder after the initial dose of alpha-chloralose. Finally, eight cats were recruited in this study. Anesthesia was induced in a chamber with 5% of isoflurane carried by 100% O_2_ and maintained with endotracheal intubation during surgery. To facilitate the metabolization of isoflurane, the surgery was limited to a duration of less than three hours. After surgery, anesthesia was induced by alpha-chloralose (Sigma C0128, Sigma-Aldrich) with initial dose of 50 mg/kg and followed by supplemental doses of 10 mg/kg as required. The alpha-chloralose powder was thoroughly dissolved in sterile saline solution (1% concentration) over approximately 30 min at a temperature of 90 °C. Subsequently, it was administered intravenously at a controlled rate of 0.5 ml/min, which effectively minimized the risk of acidosis. Gentamicin (1.2 mg/kg, SQ) and ketoprofen (5 mg/kg, IM) were administered prior to surgery.

An artificial respirator (LTV-950, Pulmonetic Systems, US) was attached to an endotracheal tube (Size 3, Biotek Medical Technology Co., China) to measure end-tidal CO_2_. Blood oxygen saturation level (SpO_2_), end-tidal CO_2_, heart rate, eye reflex, and body temperature were monitored continuously throughout the surgical process and experimental tests. A polyethylene (PE) 90 tube (inner diameter 0.9 mm, outer diameter 0.5 mm) was cannulated into the right carotid artery and connected to a pressure transducer (MX960, Smith Medical, UK) for continuous measurement of arterial blood pressure. Body temperature was measured and kept within the range of 37–38 °C by a heating pad. A pulse oximeter probe (2054, Masimo, US) was used for measuring the heart rate and blood oxygen saturation level (SpO_2_). An intravenous injection (0.9% saline, 5% dextrose; 10 ml/kg/h) was performed using a 22G angiocath canulated into the left cephalic vein. The experimental procedure in this study was terminal. At the end of the experiments, the animals were euthanized using potassium chloride (10 ml of 2 mEq/ml) under a high rate of isoflurane anesthesia.

### Bladder catheter placement

The bladder was exposed through a midline abdominal incision, and a modified suprapubic catheter (14G Angiocath) was inserted into the bladder dome and secured with a purse-string suture (4-0 silk suture, Supa, Iran). Subsequently, the abdominal wall and skin were closed in layers using 3-0 Nylon sutures (Supa, Iran). A 3-way connector was used to connect the suprapubic catheter to a pressure transducer (NovaTrans Transducer Systems MX860, Smiths Medical, UK) and an infusion pump (SN-50C6, Sino Medical-Device Technology Co., China).

**Figure 1:**
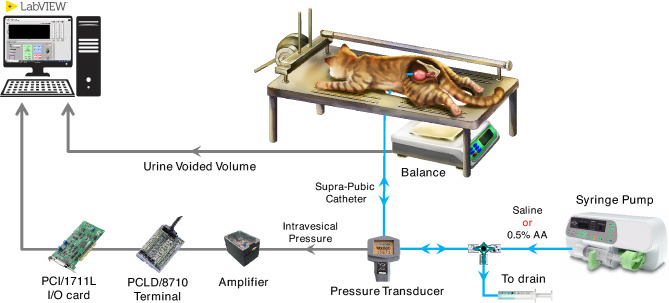
Schematic representation of the experimental setup for recording CMG trials at normal (Saline) and OAB (0.5% AA) conditions. The cat is placed on a stereotaxic setup in a prone position to measure intravesical pressure, leakage point, and voided volume. Modified suprapubic catheter, pressure transducer, syringe pump, and drain syringe are connected via a 3-way connector. A digital scale is placed beneath the cat to measure the voided volume. The residual volume can be calculated by continuously subtracting the voided volume from the filled volume. The pressure signal is first amplified and then digitalized for processing using a custom-written software in LabVIEW.

The bladder pressure was amplified 900 times using a custom-made amplifier and sampled at a rate of 50 Hz using a 12-bit analog-to-digital converter (Advantech PCI-1711L I/O card, Advantech Co., Ltd., Taiwan). A suprapubic catheter was used to prevent any blockage of the urethral outlet during each micturition reflex. Following the completion of the surgical procedure, the cat was positioned in a stereotaxic setup (SN-1N, Narishige Group Product, Japan). A digital scale (GF-300, A&D Instrument, Japan) was placed beneath the cat to measure the leakage point and voided volume. By continuously subtracting the voided volume from the injected volume, the residual volume could be calculated. All the signals were simultaneously recorded using a custom-designed LabVIEW software. The experimental setup is depicted in Fig. 1.

### Cystometrogram (CMG) testing

Prior to the start of the experiment, five CMGs were performed to recover the bladder following surgery. After the recovery period, the bladder was filled with body temperature saline (38 °C) using an infusion pump at a physiological rate ranging from 20 to 120 ml/h ($59.6\pm 32.2$ ml/h). Average of the filling rate for each cat is written in Table 1. The pump was turned off whenever urine leakage occurred. Each trial was concluded two minutes after the pump was turned off. A 15-min rest interval between fillings was considered sufficient for bladder relaxation. To create an acute OAB model, the bladder was irritated with a dilute acetic acid (AA) solution (saline with 0.5% glacial acetic acid). The experiment commenced with three preliminary trials of bladder filling using the 0.5% AA solution^38^. However, these trials were excluded from the data analysis. Three cats were randomly selected for the OAB experiment and assigned number cat 6, cat 7, and cat 8.

**Table 1:** The results of CMG test for each cat.

		#Trial	Weight (kg)	Filling rate (ml/h)	Bladder volume (ml)	Max NVC pressure (cmH_2_O)	Max VC pressure (cmH_2_O)	VC duration (s)	Max VC pressure Time (s)	Leakage pressure (cmH_2_O)	Leakage time (s)
				${\text{Mean}}$	${\text{Mean}}\pm {\text{SD}}$	${\text{Mean}}\pm {\text{SD}}$	${\text{Mean}}\pm {\text{SD}}$	${\text{Mean}}\pm {\text{SD}}$	${\text{Mean}}\pm {\text{SD}}$	${\text{Mean}}\pm {\text{SD}}$	${\text{Mean}}\pm {\text{SD}}$
Cat 1		5	$5.8$	$120.0$	$22.0\pm 4.7$	$12.3\pm 4.6$	$35.5\pm 9.5$	$56.7\pm 19.6$	$4.7\pm 1.2$	$33.6\pm 7.7$	$4.9\pm 1.1$
Cat 2		5	$4.8$	$120.0$	$13.4\pm 4.8$	$25.3\pm 15.4$	$69.5\pm 12.3$	$111.0\pm 91.9$	$3.0\pm 0.7$	$43.5\pm 11.1$	$4.2\pm 2.5$
Cat 3		5	$4.8$	$120.0$	$4.2\pm 3.1$	$26.1\pm 8.4$	$48.4\pm 7.4$	$14.8\pm 11.6$	$1.8\pm 0.4$	$34.1\pm 5.8$	$2.9\pm 0.4$
Cat 4		5	$3.7$	$60.0$	$4.0\pm 2.1$	$39.2\pm 11.7$	$69.3\pm 9.8$	$19.7\pm 15.0$	$3.3\pm 0.9$	$58.1\pm 11.8$	$3.8\pm 0.8$
Cat 5		7	$2.8$	$42.8$	$3.4\pm 0.9$	$9.4\pm 11.9$	$47.8\pm 6.3$	$34.0\pm 2.7$	$6.1\pm 1.0$	$41.0\pm 4.5$	$6.7\pm 1.4$
Cat 6	Normal	6	$2.3$	$50.0$	$4.3\pm 0.9$	$31.4\pm 8.1$	$141.8\pm 12.9$	$64.6\pm 14.3$	$5.6\pm 1.2$	$127.5\pm 10.4$	$5.8\pm 1.4$
	OAB	8		$50.0$	$1.3\pm 0.7$	$22.2\pm 7.5$	$138.3\pm 21.6$	$47.7\pm 21.9$	$3.3\pm 0.8$	$123.2\pm 12.8$	$3.9\pm 1.1$
Cat 7	Normal	10	$2.9$	$40.0$	$2.2\pm 0.5$	$5.8\pm 9.7$	$71.6\pm 17.1$	$13.4\pm 2.4$	$2.2\pm 0.6$	$46.8\pm 6.6$	$2.4\pm 0.8$
	OAB	7		$40.0$	$1.4\pm 0.2$	$0.8\pm 2.1$	$92.7\pm 17.1$	$16.3\pm 4.1$	$2.6\pm 2.2$	$51.0\pm 4.0$	$2.0\pm 0.2$
Cat 8	Normal	9	$1.9$	$41.1$	$2.4\pm 0.4$	$20.2\pm 6.0$	$84.8\pm 6.6$	$11.5\pm 3.1$	$1.9\pm 0.7$	$64.9\pm 5.6$	$2.6\pm 1.1$
	OAB	6		$30.0$	$1.6\pm 0.1$	$No NVC$	$74.1\pm 2.0$	$14.4\pm 2.6$	$1.9\pm 0.3$	$59.1\pm 1.6$	$2.3\pm 0.4$
Normal		52	$3.5$	$66.9$	$6.0\pm 6.5$	$19.4\pm 14.1$	$72.7\pm 31.3$	$36.0\pm 41.3$	$3.5\pm 1.8$	$56.8\pm 28.8$	$4.0\pm 1.9$
OAB		20	$2.4$	$40.5$	$1.7\pm 0.4$	$8.1\pm 11.5$	$103.1\pm 31.7$	$26.7\pm 20.2$	$2.7\pm 1.4$	$78.7\pm 34.5$	$2.8\pm 1.1$
Total		72	$3.6$	$59.6$	$4.8\pm 5.9$	$16.2\pm 14.3$	$81.1\pm 34.1$	$33.4\pm 36.8$	$3.2\pm 1.7$	$62.9\pm 31.8$	$3.7\pm 1.8$

### Prediction model

**Figure 2:**
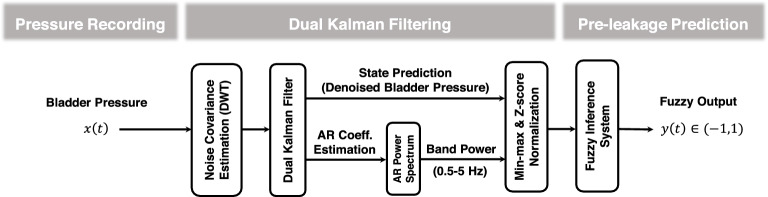
The structure of the proposed approach for urine leakage prediction from intravesical pressure.

The structure of the proposed prediction model, custom written in MATLAB (MathWorks, Natick, MA), is shown in Fig. 2. The method is based on an adaptive dual Kalman filter (DKF) for estimating the high-resolution power spectrum density (PSD) as well as denoised bladder pressure and a fuzzy inference system (FIS) for predicting urine leakage. The algorithm is sequentially applied on each new sample of the recorded bladder pressure and instantaneous band power and denoised bladder pressure sample are estimated. Then, based on the estimated instantaneous power band and pre-defined fuzzy rule base, the decision about the urine leakage is made. The rule base, which has been already defined, is used for all sessions of experiments in all cats.

For this purpose, the non-stationary bladder pressure signal was first modeled using an autoregressive dynamic model structure. This dynamic structure was selected because of its intrinsic generality and peak matching capabilities. It is desirable to estimate the formant frequency (peak) rather than the valley.

### Dynamic model of the bladder pressure

The bladder pressure signal can be described by the time-varying dynamic AR model^39^ as follows

1 $$x(n)={\sum }_{k=1}^{P}{a}_{k}(n)x(n-k)+v(n)$$

where $x(n)$ is the bladder pressure signal at time instant $n$, $P$ is the AR model order which is set to 4 in this study, ${a}_{k}$ is the time-varying model parameters, $x(n-k)$ are delayed samples of the bladder pressure signal, and $v(n)$ is an independent and normally distributed Gaussian noise with zero mean. We use DKF to estimate the time-varying AR parameters (i.e., ${a}_{k}$), in which both the states of the dynamical system and its parameters are estimated simultaneously, given only the observed signal of the bladder pressure^39^. In DKF, two separate Kalman filters run concurrently: one for state estimation and another for model parameters estimation. At every time step, the state Kalman filter estimates the state using the current estimate ${\widehat{{\text{a}}}}_{n}$, while the weight Kalman filter estimates the parameter using the current state estimate ${\widehat{x}}_{n}$ ^39^. The dynamic system (Eq. (1)) can be represented in the stat-space model as follows

2 $$\left(\begin{array}{c}x\left(n\right)\\ x\left(n-1\right)\\ x\left(n-2\right)\\ \vdots \\ x\left(n-p+1\right)\end{array}\right)=\left(\begin{array}{ccccc}{a}_{1}\left(n\right)& {a}_{2}\left(n\right)& \cdots & {a}_{p-1}\left(n\right)& {a}_{p}\left(n\right)\\ 1& 0& \cdots & 0& 0\\ 0& 1& \cdots & 0& 0\\ \vdots & \vdots & \ddots & \vdots & \vdots \\ 0& 0& \cdots & 1& 0\end{array}\right)\left(\begin{array}{c}x\left(n-1\right)\\ x\left(n-2\right)\\ \vdots \\ \vdots \\ x\left(n-p\right)\end{array}\right)+\left(\begin{array}{c}w\left(n\right)\\ 0\\ 0\\ \vdots \\ 0\end{array}\right)$$

3 $$\begin{aligned} x\left(n\right) &={\text{A}}\left(n\right)\mathbf{x}\left(n-1\right)+v\left(n\right) \\  y(n) & ={\text{H}}(n)\mathbf{x}(n)+w(n) \end{aligned}$$

where $x(n)$ is the state of the process, $y(n)$ is the observable at time *n*, ${\text{A}}(n-1)$ is the process matrix, $H(n)$ is the measurement matrix, and $w(n)$ and $v(n)$ are the process and measurement noise, respectively, which are assumed to be zero-mean Gaussian noise. The measurement matrix is $${\text{H}}\left(n\right)=\left[\begin{array}{ccc}1& 0& \begin{array}{cc}\cdots & 0\end{array}\end{array}\right]$$. The time-varying AR model coefficients and process and measurement noise variance are unknown which need to be estimated at each time step.

The parameter update for the weight Kalman filter is

4 $${\left\{\begin{array}{l}{\widehat{{\text{a}}}}_{n}^{-}= {\widehat{{\text{a}}}}_{n-1}\\ {{\text{P}}}_{{a}_{n}}^{-}={{\text{P}}}_{{a}_{n-1}}+{{\text{R}}}_{n-1}^{r}={{\lambda }^{-1}{\text{P}}}_{{a}_{n-1}}\end{array}\right.}$$

5 $$\left\{\begin{array}{l}{{\text{K}}}_{n}^{a}={{\text{P}}}_{{a}_{n}}^{-}{{(\widehat{\mathbf{x}}}_{n}^{-})}^{T}[{\widehat{\mathbf{x}}}_{n}^{-}{{\text{P}}}_{{a}_{n}}^{-}{{(\widehat{\mathbf{x}}}_{n}^{-})}^{T}+{{\text{R}}}_{n}^{e}{]}^{-1}\\ {\widehat{{\text{a}}}}_{n}={\widehat{{\text{a}}}}_{n}^{-}+{{\text{K}}}_{n}^{a}\left[{y}_{n}-{\widehat{\mathbf{x}}}_{n}^{-}{\widehat{{\text{a}}}}_{n}^{-}\right]={\widehat{{\text{a}}}}_{n}^{-}+{{\text{K}}}_{n}^{a}{e}_{n}\\ {{\text{P}}}_{{a}_{n}}=\left[{\text{I}}-{{\text{K}}}_{n}^{a}{\widehat{\mathbf{x}}}_{n}^{-}\right]{{\text{P}}}_{{a}_{n}}^{-}\end{array}\right.$$

where ${\widehat{{\text{a}}}}_{n}^{-}$ is a prior parameter estimate at time step $n$ given knowledge of the process prior to step $n$, ${\widehat{{\text{a}}}}_{n}$ is a posterior parameter estimate at time step $n$ given measurement ${y}_{n}$, ${{\text{K}}}_{n}^{a}$ is a Kalman gain specified for the parameter equation, ${\widehat{x}}_{n}^{-}$ is a prior state estimate at step $n$ given the knowledge of the process prior to step $n$, ${y}_{n}$ is the measurement at time instant $n$, and $\lambda$ is the forgetting factor.

**Figure 3:**
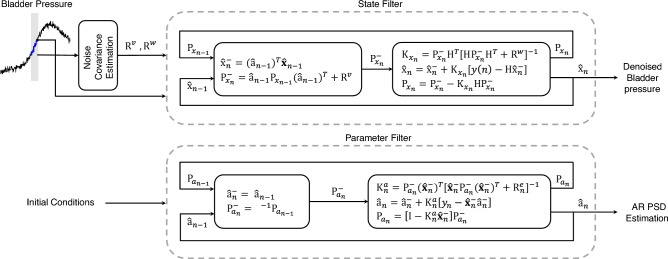
Structure of the adaptive DKF for estimating AR coefficients and denoised bladder pressure.

The state update for the state Kalman filter is

6 $$\left\{\begin{array}{l}{\widehat{{\text{x}}}}_{n}^{-}=({\widehat{{\text{a}}}}_{n-1}{)}^{T}{\widehat{\mathbf{x}}}_{n-1}\\ {{\text{P}}}_{{x}_{n}}^{-}={\widehat{{\text{a}}}}_{n-1}{{\text{P}}}_{{x}_{n-1}}({{\widehat{{\text{a}}}}_{n-1})}^{T}+{{\text{R}}}^{v}\end{array}\right.$$

7 $$\left\{\begin{array}{l}{{\text{K}}}_{{x}_{n}}={{\text{P}}}_{{x}_{n}}^{-}{{\text{H}}}^{T}[H{{\text{P}}}_{{x}_{n}}^{-}{{\text{H}}}^{T}+{{\text{R}}}^{w}{]}^{-1}\\ {\widehat{{\text{x}}}}_{n}={\widehat{{\text{x}}}}_{n}^{-}+{{\text{K}}}_{{x}_{n}}[y\left(n\right)-H{\widehat{{\text{x}}}}_{n}^{-}]\\ {{\text{P}}}_{{x}_{n}}={{\text{P}}}_{{x}_{n}}^{-}-{{\text{K}}}_{{x}_{n}}H{{\text{P}}}_{{x}_{n}}^{-}\end{array}\right.$$

where ${{\text{K}}}_{\widehat{x},n}$ is Kalman gain specified for state equation, ${\widehat{x}}_{n}$ is a posterior state estimate at time step $n$ given measurement ${y}_{n}$, ${{\text{P}}}_{\widehat{x},n}^{-}$ is a prior estimate error covariance for state equation, ${{\text{P}}}_{\widehat{x},n}$ is a posterior estimate error covariance for state equation, and ${{\text{R}}}^{v}=q\mathbf{I}$ and ${{\text{R}}}^{w}$ are process and measurement noise covariance for state equation, respectively. The structure of DKF is schematically is shown in Fig. 3.

### Noise covariance estimation

In this study, we employ the discrete wavelet transform (DWT) to estimate the measurement noise. The DWT is a digital implementation technique for wavelet transform. It decomposes a signal into a set of mutually orthogonal wavelet basis functions by a convolution between an original signal and two filters: a high-pass filter that computes wavelet detail coefficient and a low-pass filter that computes wavelet approximation coefficient. To estimate the measurement noise, at each time step, the DWT using Daubechies-3 mother wavelet is applied to a sliding window. The variance of the reconstructed bladder pressure, obtained using the detail coefficient at the first level of decomposition, is considered as the estimated variance of the measurement noise (i.e., $${R}^{w})$$.

### Instantaneous PSD estimation

The AR PSD of the bladder pressure using the estimated AR parameters by the DKF is used to analyze the time–frequency representation of the bladder pressure during bladder filling. The average of the power spectrum at the band (0.5–5 Hz) is used as the feature for leakage prediction. This band is selected because the bladder changes occur below 5 Hz^40^. To adjust the data recorded from different cats to a common scale and improve the generalizations of the prediction mode, the recorded bladder pressure and the estimated band power spectrum were normalized. For this purpose, the mean and variance of the pressure, as well as the estimated band power spectrum, were calculated from the first trial of the experiment for each cat, and these values were used for the Z-score normalization of the data. Then, the data underwent min–max normalization to scale them between 0 and 1.

### Fuzzy leakage prediction

**Figure 4:**
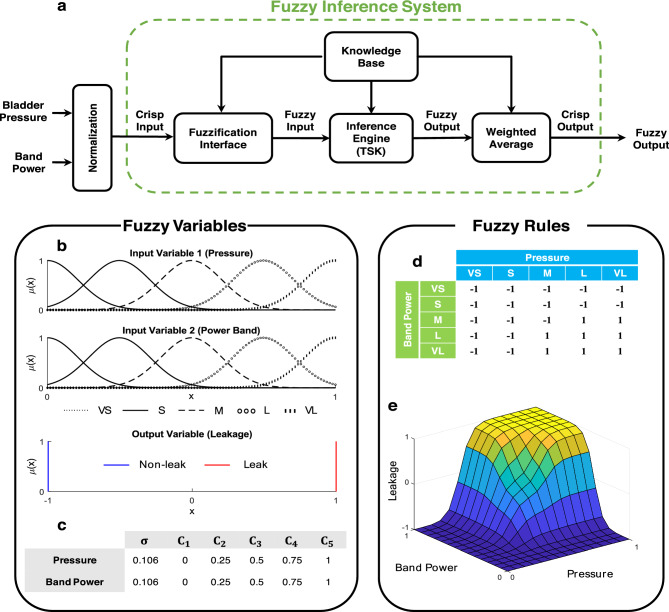
(**a**) Fuzzy inference system structure includes fuzzification interface, TSK inference engine, and defuzzification interface. (**b**) Fuzzy sets and membership functions for fuzzy input and output variables. VS (very small), S (small), M (medium), L (large), and VL (very large) are fuzzy sets for input variables. 1 and − 1 represent leakage and non-leakage events for output variable, respectively. (**c**) Membership function parameters for fuzzy logic classifier's input variables. (**d**) Fuzzy rules table for urine leakage prediction. (**e**) Three-dimensional surface of fuzzy rules.

In this study, we use FIS to predict bladder urine leakage. Fuzzy models offer a different perspective on modeling complicated systems. Their simplicity and ease of use without requiring training make them well-suited for real-time processes and biomedical applications that involve black-box systems. A fuzzy inference system consists of three basic components: inference rule base, fuzzification, and defuzzification (Fig. 4a). The fuzzification and defuzzification are the interfaces between the fuzzy systems and the crisp systems. The rule base includes a set of “*If*… *Then*…” rules. Each rule describes a relation between the input fuzzy sets and the output fuzzy sets. Takagi, Sugeno and Kang (TSK)^41^ is one of the most used fuzzy modeling techniques that employs If–Then rules with fuzzy antecedents and a mathematical function in the form:

8 $$\begin{aligned} & {R}^{i}:\hspace{1em}If\hspace{0.33em}{x}_{1}\hspace{0.33em}is\hspace{0.33em}{A}_{1}^{i}\hspace{0.33em}and\hspace{0.33em}{x}_{2}\hspace{0.33em}is\hspace{0.33em}{A}_{2}^{i}\hspace{0.33em}and\hspace{0.33em}\cdots \hspace{0.33em}and\hspace{0.33em}{x}_{r}\hspace{0.33em}is\hspace{0.33em}{A}_{r}^{i} \\ &Then \; {y}^{i}={a}_{0}^{i}+{a}_{1}^{i}{x}_{1}+{a}_{2}^{i}{x}_{2}+\cdots +{a}_{r}^{i}{x}_{r}\hspace{1em}for\hspace{1em}i=1, 2,\cdots ,k \end{aligned}$$

where $k$ is the number of rules, ${x}_{j}(j=1, 2,\dots ,r)$ is the $j$ th input, ${y}^{i}$ is the output of fuzzy rules ${R}^{i}$, ${\mu }_{{A}_{1}^{i}},{\mu }_{{A}_{2}^{i}},\dots ,{\mu }_{{A}_{r}^{i}}$ are fuzzy sets that are characterized by the membership function ${\mu }_{{A}_{j}^{i}}({x}_{j})$. TSK does not require a defuzzification process and the ${\mu }_{{A}_{1}^{i}}$ output is calculated by a linear combination of the consequents^41^. The final output of the TSK fuzzy system ($y$) is computed as follows:

9 $$y=\frac{\sum_{i=1}^{k}{y}^{i}\left(\prod_{j=1}^{r}{\mu }_{{A}_{j}^{i}}({x}_{j})\right)}{\sum_{i=1}^{k}\left(\prod_{j=1}^{r}{\mu }_{{A}_{j}^{i}}({x}_{j})\right)}$$

The complete structure of the FIS is shown in Fig. 4.

### Fuzzy sets and variables

In the proposed fuzzy inference system, inputs are the pressure and the spectral power at the band (0.5–5 Hz) and the output is the estimated leakage point which can take the value between 1 and − 1. The values 1 and − 1 represents the highest probability of being associated with leakage and non-leakage event, respectively. By setting a constant threshold (zero) on fuzzy outputs, outputs greater or equal to zero classify as leakage events. Gaussian membership functions are selected for the input variables as

10 $$\mu _{A}^{i} \left( {x_{j} } \right) = \exp \left[ { - \frac{1}{2}\left( {\frac{{x_{j}  - c^{i} }}{{\sigma ^{i} }}} \right)^{2} } \right]$$

where “$A$” represents the fuzzy sets depicted in Fig. 4b, ${c}^{i}$ and ${\sigma }^{j}$ are the center and the width of the $i$th Gaussian membership function, respectively, and ${x}_{j}$ represents bladder pressure or band power. The membership functions parameters (i.e., ${c}^{i}$ and ${\sigma }^{j}$) are fixed and tabulated in Fig. 4c.

### Fuzzy rules

Using a predefined rule table (Fig. 4d), the inference engine maps the input fuzzy sets to an output fuzzy set. The fuzzy rules were heuristically defined based on the prior knowledge obtained from the literatures and expert, then tuned from data obtained from trial 1 of cat 1, thereafter used for prediction on all cats without any re-adjustment. Fuzzy rules 3D surface is shown in Fig. 4e.

### Performance evaluation

**Figure 5:**
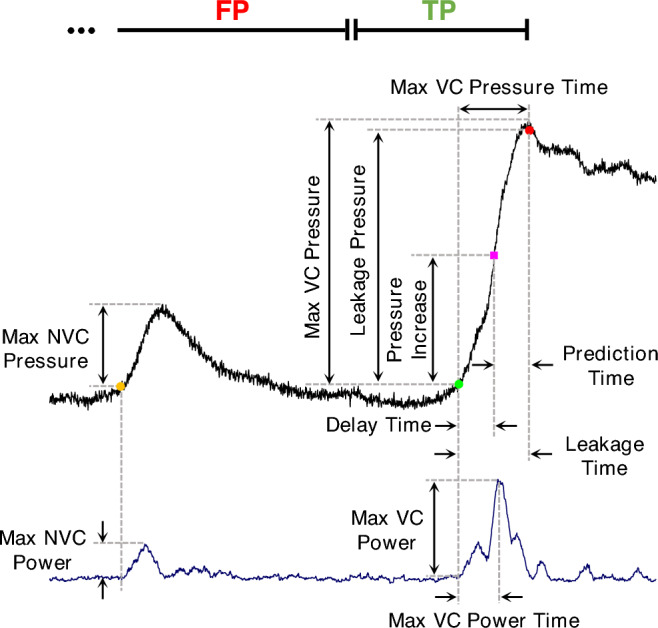
Different measures for evaluating the CMG trials and proposed method in leakage prediction: Leakage point (red circle), predicted leakage (pink square), start of VC (green circle), start of NVC (orange circle), bladder pressure (black line), and bladder band power (blue line). A leakage detection during the pre-leakage is considered as the true positive (TP) and during the non-leakage or post-leakage interval as the false positive (FP). A prediction during post-leakage is considered as FP if no leakage is predicted before the first leakage.

To assess the performance of the proposed method in prediction of the urine leakage, the bladder pressure signal is divided into three intervals (Fig. 5):

- Pre-leakage interval: The 5-s interval prior to the onset of first leakage point.
- Non-leakage interval: The interval preceding the pre-leakage interval.
- Post-leakage interval: The interval after the first leakage point.

Then, the following performance criteria are defined as

11 $$Success\; Rate(\%)=\frac{TP}{TP+FP}$$

12 $$Sesitivity(\%)=\frac{TP}{TP+FN}$$

13 $$Specificity(\%)=\frac{TN}{TN+FP}$$

where

- True positive (TP): Prediction during the pre-leakage interval.
- True negative (TN): Non-prediction during non-leakage interval.
- False positive (FP): A leakage prediction during the non-leakage or post-leakage interval. A prediction during post-leakage is considered as FP if no leakage is predicted before the first leakage.
- False negative (FN): Non-prediction during pre-leakage interval.

To determine the starting points of NVC and VC, the instants of the band power that crosses the baseline power value were manually determined from the band power plot and were assigned the starting points of NVC and VC. Moreover, different measures were also reported to evaluate the proposed method as follows (Fig. 5):

- Leakage pressure (i.e., the pressure at the time instant of the first leakage point)
- Leakage power (i.e., the band power at the time instant of the first leakage point)
- Leakage time (i.e., the first urine leakage time relative to the start of VC),
- Max VC pressure, (i.e., the maximum pressure associated with the VC),
- Max VC power, (i.e., the maximum band power associated with the VC),
- Max VC pressure time (i.e., the time of the maximum pressure relative to the start of VC),
- Max VC power time (i.e., the time of the maximum band power relative to the start of VC),
- Max NVC pressure (i.e., the maximum pressure associated with the largest NVC),
- Max NVC power (i.e., the maximum band power associated with the largest NVC),
- Leakage prediction time (i.e., the urine leakage prediction time relative to the actual leakage time),
- Delay time (i.e., urine leakage prediction time relative to the start of VC),
- Pressure increase (i.e., the bladder pressure at the instant of the predicted leakage point),
- Normalized leakage pressure (i.e., pressure increase/max VC pressure),
- Normalized delay time (i.e., delay time/leakage time).

One-way ANOVA followed by the post-hoc Tukey–Kramer’s multiple comparison test was used to assess the statistical difference of the results, and $p<0.05$ (one star), $p<0.01$ (two stars), $p<0.001$ (tree stars) indicated a significant difference.

## Results

### CMG test

**Figure 6:**
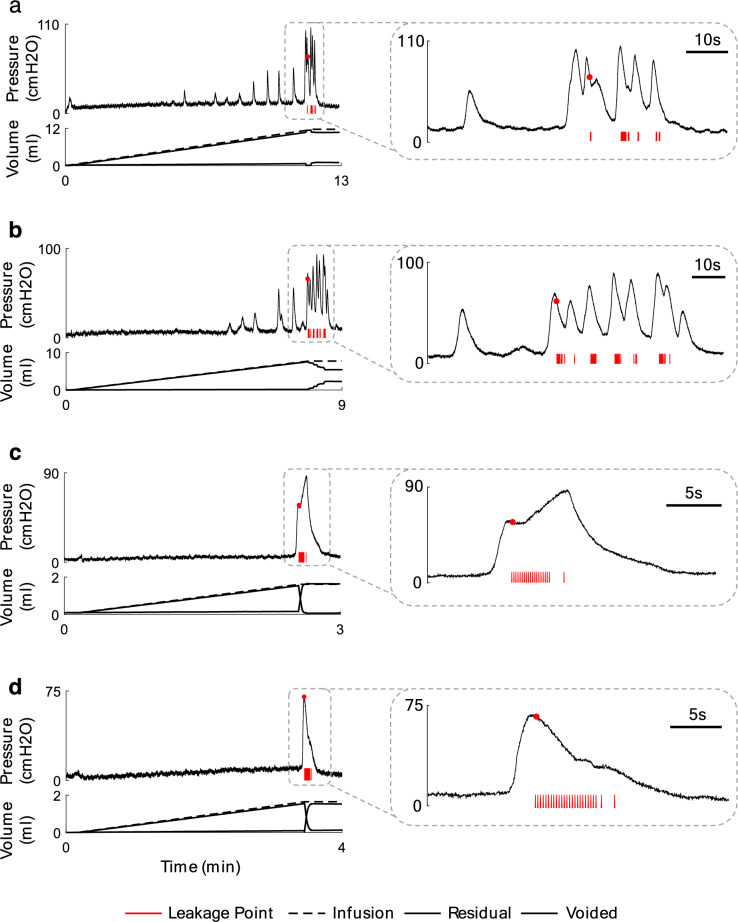
An example of the bladder CMG during normal condition in cat 2, trial 4 (**a**) and cat 4, trial 1 (**b**). An example of the CMG during OAB condition in cat 7, trial 1 (**c**) and cat 8, trial 5 (**d**). The right plots show the close-up view of a segment of the recorded bladder pressure.

In total, 72 CMG trials of the experiment were conducted on 8 cats (52 trials with saline infusion in cats 1–8 and 20 trials with 0.5% AA infusion in cats 6–8) with an open urethra. Figure 6 shows typical CMG trials with saline (normal) and AA (OAB) infusions. It can be seen that the dynamics of the bladder pressure with saline infusion are different from that with AA infusion. NVCs before voiding contraction are observed during bladder filling with saline (Fig. 6a,b), but the bladder does not show NVC during filling with AA (Fig. 6c,d). All 72 trials of experiment can be seen in the supplementary materials in normal (Supplementary Fig. S1) and OAB (Supplementary Fig. S2) conditions.

Table 1 summarizes the measured parameters during CMG tests in normal and OAB conditions including bladder volume, max NVCs pressure, max VC pressure, VC duration, max VC pressure time, leakage pressure, and leakage time. The range of the bladder volume, max VC pressure, VC duration, max VC pressure time, leakage pressure, and the leakage time vary from 1.6 to 29.4 ml ($6.0\pm 6.5$ ml), 34.1 to 160.9 cmH_2_O ($72.7\pm 31.3$ cmH_2_O), 7.6 to 235.5 s ($36.0\pm 41.3$ s), 1.2 to 7.4 s ($3.5\pm 1.8$ s), 23.2 to 139.9 cmH_2_O ($56.8\pm 28.8$ cmH_2_O), and 1.2 to 8.6 s ($4.0\pm 1.9$ s), respectively, during saline infusion (cat 1–8, 52 trials).

In OAB condition bladder volume, max VC pressure, VC duration, max VC pressure time, leakage pressure, and the leakage time ranged from 1.2 to 2.6 ml ($1.7\pm 0.4$ ml), 64.0 to 181.7 cmH_2_O ($103.1\pm 31.7$ cmH_2_O), 10.2 to 79.4 s ($26.7\pm 20.2$ s), 1.1 to 6.2 s ($3.5\pm 1.8$ s), 44.2 to 141.5 cmH_2_O ($56.8\pm 28.8$ cmH_2_O), and 1.7 to 6.0 s ($2.8\pm 1.1$ s), respectively (cat 6–8, 20 trials).

**Figure 7:**
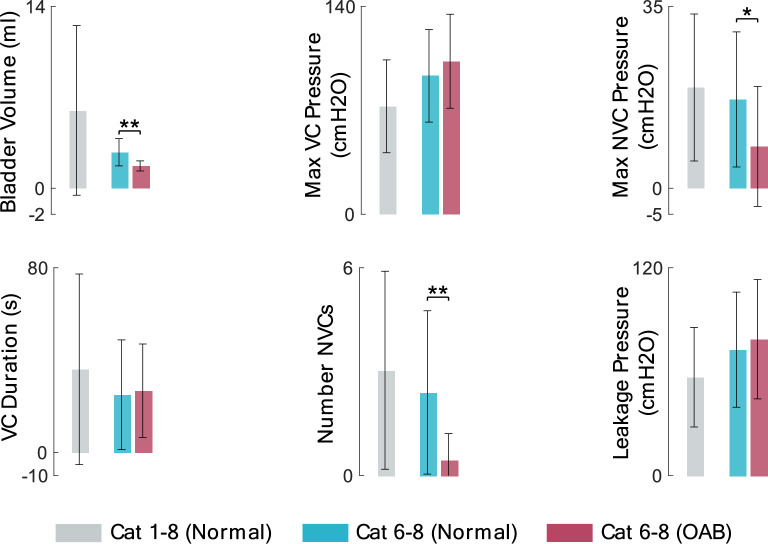
Average of the measured values from CMG test over 52 normal trials in 1–8 cats (gray bar), 20 normal trials in cat 6–8 (cyan bar), and 20 OAB trials in cat 6–8 (red bar).

Bar plot of the measured parameters is shown in Fig. 7. The results indicate that there is significant difference between the number of NVC during the saline and AA infusion ($$p<0.01)$$. The filling time between normal ($$3.8\pm 1.1$$ min) and OAB ($$2.7\pm 0.9$$ min) trials exhibits a significant difference ($$p<0.01$$). Therefore, the lower number of NVCs may related to the very small OAB volumes, as there is not sufficient filling time for NVCs to occur. Also, Max NVC pressures during saline and AA infusions are significantly different ($$p<0.05$$). The leakage pressure as well as the VC duration is not significantly different.

The results point out that there is no significant difference between max VC pressures during filling with saline and AA. It has been already reported that there is no significant difference in the max VC pressures in saline and AA in cats^38^ and rats^42^. Bladder volume significantly reduces when filled with AA ($p<0.001$), indicating the ability of AA to simulate OAB in cats. This observation is in agreement with other studies reporting the significant reduction in bladder capacity during filling with AA in cats^38^ and rats^42^.

### Time–frequency analysis

**Figure 8:**
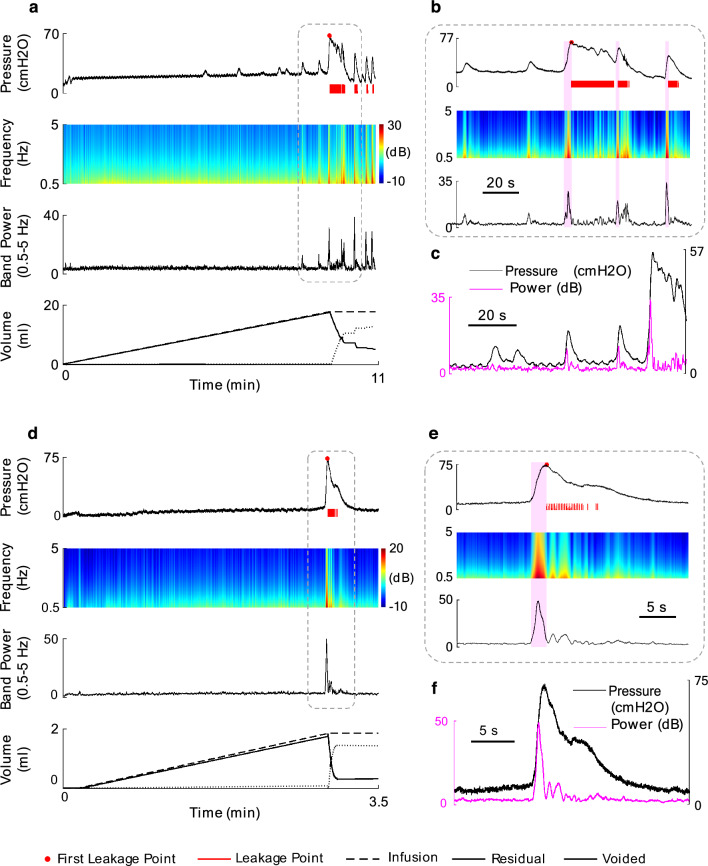
The results of the time–frequency analysis of a typical bladder pressure during normal and OAB conditions using DKF in cat 1, Trial 2 (**a**) and cat 8, trial 6 (**d**), respectively. (**c**) and (**f**) provides a close-up view of the time–frequency analysis. (**b**) and (**e**) comprise the pressure versus power band.

Figure 8 shows the AR PSD of the bladder pressure using DKF during a typical trial of an experiment with saline (cat 1, trial 2, Fig. 8a) and AA (cat 8, trial 6, Fig. 8d) infusions. The plots also show the recorded bladder pressure, leakage point, the infused volume, the residual volume, the voided volume, and the average of AR PSD at the band (0.5–5 Hz). The time–frequency analysis based on DKF shows different patterns of event-related synchronization (ERS) associated with the phasic (prodromal) and non-phasic (non-voiding) contractions. During this trial of the experiment with saline infusion (cat 1, trial 2, Fig. 8a), the max VC pressure and the max NVC pressure were 45.1 cmH_2_O and 14.5 cmH_2_O, respectively. Max VC power and the max NVC power were 26.9 dB and 9.1 dB, respectively. The leakage observed 3.9 s after the start of the contraction, when the bladder volume reached 17.8 ml. The leakage occurred 1.7 s after the max VC power and 0.1 s after the max VC pressure. During simulating OAB condition with AA infusion (cat 8, trial 6, Fig. 8d), the max VC pressure and the max NVC pressure were 73.4 cmH_2_O and 0.0 cmH_2_O, respectively. Max VC power and the max NVC power are 54.2 dB and 0.0 dB, respectively. Furthermore, the leakage took place 2.4 s after the start of the contraction, when the bladder volume reached 1.7 ml. This leakage event occurred 1.3 s after the max VC power and 0.5 s after the max VC pressure.

Table 2 summarizes the results of the time–frequency analysis of the bladder pressure using DKF for both conditions. The time–frequency analysis of all 72 trials of the experiment can be seen in the supplementary materials in normal (Supplementary Fig. S1) and OAB (Supplementary Fig. S2) conditions.

**Table 2:** The results of measured values extracted from time–frequency analysis of the bladder pressure during CMG tests using DKF for each cat.

		#Trial	VC Max power (dB)	VC max power time (s)	NVC max power (dB)	Leakage power (dB)
			${\text{Mean}}\pm {\text{SD}}$	${\text{Mean}}\pm {\text{SD}}$	${\text{Mean}}\pm {\text{SD}}$	${\text{Mean}}\pm {\text{SD}}$
Cat 1		$5$	$19.1\pm 9.5$	$2.6\pm 1.1$	$5.9\pm 3.4$	$5.8\pm 2.7$
Cat 2		$5$	$30.5\pm 2.7$	$1.7\pm 0.8$	$10.3\pm 7.4$	$16.1\pm 3.6$
Cat 3		$5$	$46.0\pm 3.9$	$1.7\pm 0.4$	$15.3\pm 6.7$	$19.0\pm 9.7$
Cat 4		$5$	$23.8\pm 9.3$	$1.6\pm 0.7$	$11.4\pm 1.0$	$7.0\pm 2.8$
Cat 5		$7$	$7.3\pm 1.9$	$4.4\pm 2.1$	$0.6\pm 1.1$	$3.9\pm 1.3$
Cat 6	Normal	$6$	$60.9\pm 7.7$	$4.1\pm 1.7$	$11.6\pm 4.6$	$8.1\pm 4.6$
	OAB	$8$	$60.7\pm 7.3$	$2.4\pm 0.9$	$8.2\pm 6.6$	$8.0\pm 3.8$
Cat 7	Normal	$10$	$31.1\pm 9.8$	$1.5\pm 0.6$	$2.1\pm 3.7$	$8.1\pm 6.6$
	OAB	$7$	$36.5\pm 11.1$	$1.1\pm 0.1$	$0.8\pm 2.2$	$6.2\pm 4.9$
Cat 8	Normal	$9$	$49.0\pm 13.1$	$1.4\pm 0.7$	$6.2\pm 4.1$	$6.0\pm 2.9$
	OAB	$6$	$48.2\pm 12.7$	$1.1\pm 0.2$	$No NVC$	$5.3\pm 1.9$
Normal		$52$	$34.0\pm 18.4$	$2.4\pm 1.6$	$7.0\pm 6.2$	$8.6\pm 6.5$
OAB		$20$	$48.5\pm 14.4$	$1.6\pm 0.8$	$3.2\pm 5.5$	$6.6\pm 3.8$
Total		$72$	$38.0\pm 18.4$	$2.1\pm 1.5$	$5.9\pm 6.2$	$8.1\pm 5.9$

**Figure 9:**
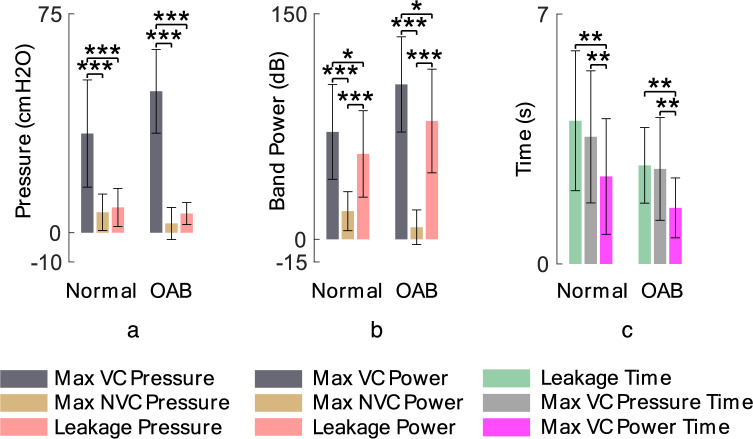
Average of the pressure parameters (**a**), band power parameters (**b**), time parameters (**c**) in normal (8 cats, 52 trials) and OAB (3 cats, 20 trials) cats during CMG tests. The figure does not present a comparison between the normal and OAB results.

**Figure 10:**
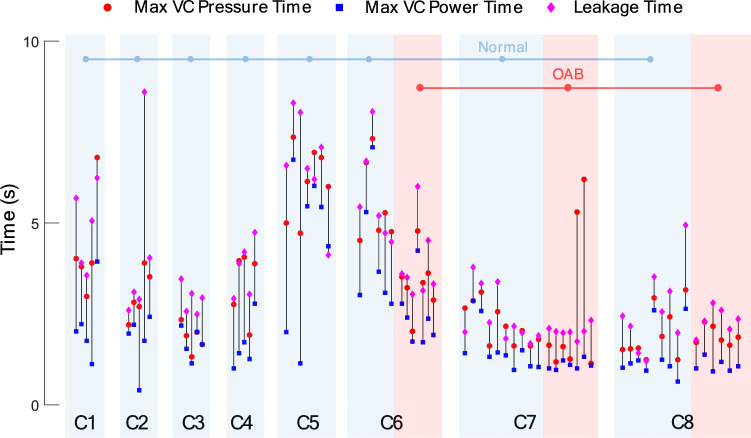
The event times occurred for each cat and each trial of experiment (52 normal trials in 8 cats and 20 OAB trials in 3 cats) including leakage time, max VC pressure time, and max VC power time. Each vertical line represents one trial, with the pink diamond, red circle, and blue square indicating leakage time, max VC pressure time, and max VC power time, respectively.

Figure 9a shows the average of the max VC pressure, max NVC pressure, and the leakage pressure over all cats during saline infusion as well as during AA infusion. It can be seen that, in both normal and OAB condition, the average of the leakage pressure is significantly lower than the max VC pressure ($$p<0.$$05) and significantly higher than the max NVC pressure ($$p<0.$$001). Also, the average of max VC pressure is significantly higher than the max NVC pressure ($$p<0.$$001). But there is no significant difference between the max NVC power and the leakage point power (Fig. 9b). However, the average of the leakage power is significantly lower than the max VC power ($$p<0.$$001). Figure 9c shows that the leakages occurred after the max VC pressure time as well as the max VC power time in all cases. The results show that the leakage time and the max VC power time (Fig. 9c) are significantly different ($$p<0.01)$$ for both normal and OAB conditions, but there is no significant difference between the max VC pressure time and the leakage time ($$p = 0.227$$ for normal and $$p = 0.798$$ for OAB). Even, the max VC power time is significantly earlier than the max VC pressure time ($$p<0.001$$). These evidences indicate that the DKF-PSD can be a suitable criterion for leakage prediction. Figure 10 shows the event time for all normal and OAB cats.

### Fuzzy prediction of urinary leakage

**Figure 11:**
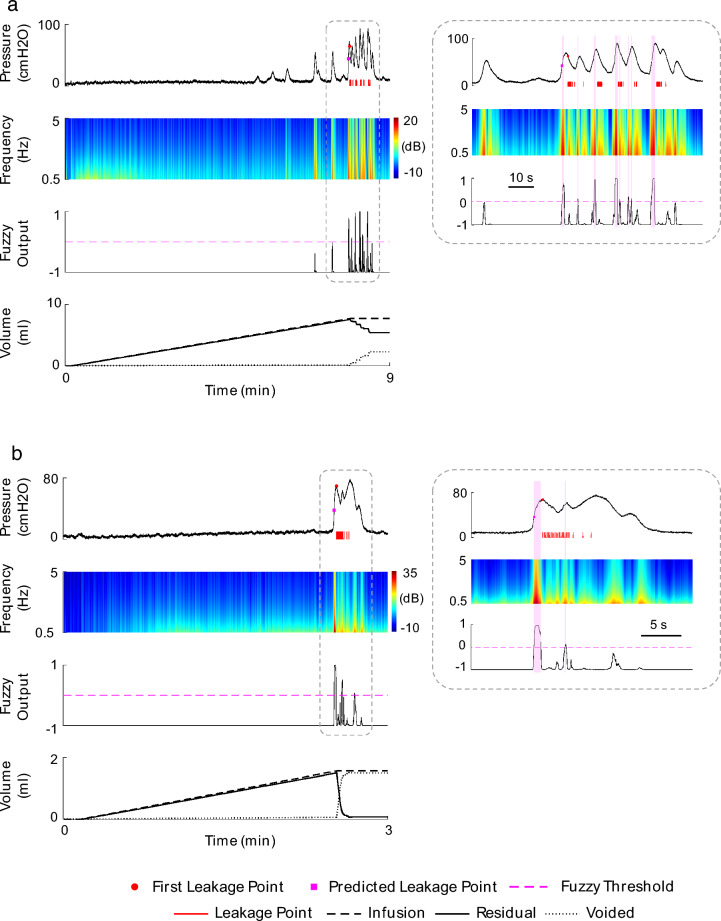
The results of the urine leakage prediction for a typical trial of experiment during normal (**a**) and OAB (**b**) conditions. Right plots show a close-up view of a selected segment of the bladder pressure and its corresponding time–frequency.

Typical results of leakage prediction using proposed method for a trial of the experiment conducted under normal (cat 4, trial 1) and OAB (cat 8, trial 1) conditions are shown in Fig. 11.

**Figure 12:**
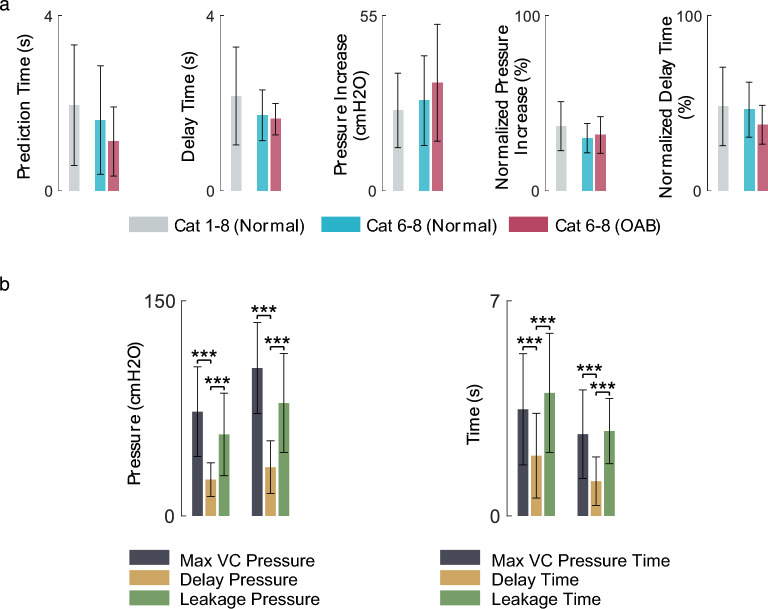
(**a**) Average of the prediction performance over 52 normal trials in 8 cats (gray bar), 20 normal trials in 3 cats (cyan bar), and 20 OAB trials in 3 cats (red bar). (**b**) Average of the maximum pressure (max VC pressure), average pressure at the instant of leakage (delay pressure), average pressure at the instant of leakage prediction (leakage pressure), average time that the max VC pressure has occurred (max VC pressure time), average time that the leakage has occurred (delay time), and average time of the leakage perdition (leakage prediction).

According to the defined measures (Fig. 5), the fuzzy prediction time, delay time, pressure increase, normalized leakage pressure, normalized delay time, and success rate for each cat and for both normal and OAB conditions are summarized in Table 3. The average of measured values is also shown in a bar plot in Fig. 12.

**Table 3:** The performance of the proposed method in leakage prediction for each cat.

		#Trial	Prediction time (s)	Delay time (s)	Pressure increase (cmH_2_O)	Normalized pressure increase (%)	Normalized delay time (%)	Success rate (%)
			${\text{Mean}}\pm {\text{SD}}$	${\text{Mean}}\pm {\text{SD}}$	${\text{Mean}}\pm {\text{SD}}$	${\text{Mean}}\pm {\text{SD}}$	${\text{Mean}}\pm {\text{SD}}$	
Cat 1		$5$	$3.1\pm 0.9$	$1.8\pm 1.2$	$13.3\pm 2.8$	$41.0\pm 18.4$	$34.5\pm 16.9$	$100$
Cat 2		$5$	$4.8\pm 3.0$	$1.4\pm 0.4$	$24.3\pm 4.1$	$35.2\pm 4.3$	$40.4\pm 21.7$	$40$
Cat 3		$5$	$1.3\pm 0.2$	$2.1\pm 1.0$	$24.9\pm 7.4$	$53.2\pm 20.6$	$75.1\pm 45.8$	$100$
Cat 4		$5$	$2.2\pm 0.4$	$1.5\pm 0.9$	$30.9\pm 7.9$	$45.5\pm 14.2$	$38.9\pm 14.2$	$100$
Cat 5		$7$	$2.8\pm 1.2$	$3.9\pm 1.6$	$20.0\pm 7.4$	$42.1\pm 14.6$	$57.7\pm 16.4$	$100$
Cat 6	Normal	$6$	$2.4\pm 0.4$	$2.3\pm 1.3$	$49.7\pm 10.8$	$34.8\pm 6.0$	$56.2\pm 8.8$	$100$
	OAB	$8$	$2.0\pm 0.3$	$1.9\pm 0.9$	$57.4\pm 6.9$	$41.9\pm 5.3$	$46.3\pm 12.8$	$100$
Cat 7	Normal	$10$	$1.4\pm 0.2$	$1.0\pm 0.6$	$17.3\pm 3.7$	$26.2\pm 10.6$	$38.4\pm 12.7$	$100$
	OAB	$7$	$1.4\pm 0.2$	$0.6\pm 0.1$	$18.0\pm 2.7$	$19.9\pm 4.7$	$30.3\pm 6.3$	$100$
Cat 8	Normal	$9$	$1.6\pm 0.5$	$1.2\pm 0.5$	$26.5\pm 2.8$	$31.5\pm 5.0$	$48.9\pm 18.7$	$100$
	OAB	$6$	$1.5\pm 0.2$	$0.9\pm 0.2$	$25.4\pm 1.9$	$34.4\pm 3.2$	$36.7\pm 6.7$	$100$
Normal		$52$	$2.2\pm 1.1$	$2.0\pm 1.4$	$25.3\pm 11.7$	$37.0\pm 14.0$	$48.3\pm 22.5$	$94.2$
OAB		$20$	$1.6\pm 0.4$	$1.1\pm 0.8$	$34.0\pm 18.4$	$32.0\pm 10.5$	$37.8\pm 11.1$	$100$
Total		$72$	$2.0\pm 1.0$	$1.7\pm 1.3$	$27.7\pm 14.3$	$35.6\pm 13.2$	$45.4\pm 20.4$	$95.8$

The supplementary materials present the outcomes of the fuzzy prediction conducted on all 72 experiment trials. These results are showcased for the saline infusions (normal) in Supplementary Fig. S1 and for the AA infusions (OAB) in Supplementary Fig. S2.

Using the Fuzzy algorithm, on average in normal condition (saline infusion), the urine leakage was predicted $2.1\pm$ 1.0 s before its occurrence and $2.0\pm 1.4$ s after the start of the contraction (delay time) at the bladder pressure of $25.3\pm 11.7$ cmH_2_O (pressure increase) with the success rate, sensitivity, and specificity of 94.2%, 100%, and 94.2%. The average values of normalized pressure increase, and the normalized delay time were $37.0\pm 14.0$% and $48.3\pm 22.5$%, respectively (cat 1–8, 52 trials).

The proposed method could predict urine leakage in OAB cats (AA infusion) $1.6\pm 0.4$ s before its occurrence and with a delay of $1.1\pm 0.8$ s from the start of the contraction at the pressure of $34.0\pm 18.4$ cmH_2_O (pressure increase) with the success rate, sensitivity, and specificity of 100%, 100%, and 100%. The average values of normalized pressure increase and the normalized delay time were $32.0\pm 10.5$% and $37.8\pm 11.1$%, respectively (cat 6–8, 20 trials).

The results show (Fig. 12b) that the leakage pressure is significantly lower than the max VC pressure for both normal ($p<0.001$) and OAB ($p<0.001$) cats. Furthermore, it is evident from Fig. 12b that the delay time is significantly shorter than the leakage time in both normal ($p<0.001$) and OAB ($p<0.001$) cats.

## Discussion

In this paper, a method based on a fuzzy inference system and dual Kalman filtering was proposed for urine leakage prediction during CMG experiments. Two variables including high-resolution power spectrum estimated by DKF and denoised bladder pressure were considered as the input and the leakage time as the output of the fuzzy inference system. In contrast to the previous studies^23–35^, in which long-lasting prediction or the estimated onset of the bladder contraction was used as the stimulation trigger signal, the proposed method predicts the instant of the urine leakage. The main motivation in the current study was to provide a short-term prediction of the leakage to shorten the stimulation duration and minimize the delivered stimulation which results in battery consumption reduction and reduces the tissue fatigue as well as risk of the tissue damage. Although, the model was applied offline to the previously collected dataset, but the model can be used in a real-time scenario as it showed its ability to predict urine leakage in new unseen data.

The analyses on the 72 trials of experiment in this study show that the $84.7\%$ of the leakages occur $0.8\pm 0.9$ s and $85.0\%$ of them $0.6\pm 0.4$ s after the maximum voiding contraction during saline and AA infusion, respectively, and $4.0\pm 1.9$ s and $2.8\pm 1.1$ s after the start of contraction. The time–frequency analysis show that the leakage occurs $1.7\pm 1.4$ s and $1.2\pm 0.4$ s after the maximum voiding contraction power during saline and AA infusion, respectively. The proposed method predicts the leakage $2.2\pm 1.1$ s and $1.6\pm 0.4$ s before its occurrence and $2.0\pm 1.4$ s and $1.1\pm 0.8$ s after the start of contraction in normal and OAB conditions, respectively.

A major advantage of the proposed method is that it is free from defining a threshold and does not require pre-training as in the previous methods^23–35^. Inter-subject and intra-subject variability reduce the generalization ability of the threshold-based methods seriously, which further limited the applications of methods in real life. The proposed fuzzy method is based on predefined rules, and these general rules are utilized for prediction in all trials of the experiment during both normal and OAB conditions in all cats.

A critical issue in estimation the start of bladder contraction as well as the leakage prediction is NVCs. In some studies^30,32,35^, prodromal NVCs are utilized as a criterion for detecting VCs. However, detection is in a range of 100 s^30^ or 25 s^35^ before occurrence of voiding which consequently requires too long stimulation duration to control incontinence. Our high-resolution time–frequency analyses using DKF show that the time–frequency pattern of the NVCs and VCs are significantly different. Consequently, the utilization of this pattern difference within the proposed method enables the prediction of leakage points with a high success rate of 94.2% in normal and 100% in OAB.

Three trials in cat 2 were rare cases that were characterized by prolonged, high-amplitude pressure where the leakage was occurred either midway or at the end of the duration (see Supplementary Fig. S1, cat 2, trials 1, 2, and 3). However, such contraction dynamics were not observed in the subsequent trials of this cat (trials 4 and 5). In these three trials, the algorithm predicted urine leakage during non-leakage intervals (FP), which occurred slightly before high-amplitude voiding contractions.

In this study, different subjects with different levels of bladder volume were engaged (1.6 to 29.4 ml). Different levels of bladder volume have been reported in the literatures. For example, the average bladder volumes of 13.8 ml and 12.6 ml were reported in other works^12,14^, respectively. It should be noted that different factors such as ethic, weight, maintenance protocol before surgery, anesthesia depth, anesthesia duration may affect the bladder volume. As suggested in Xu et al.^43^, previously administered agents, may reduce the sensation of bladder filling for future trials. The average of bladder volumes in this study were $6.0\pm 6.5$ ml in normal and $1.7\pm 0.4$ ml in OAB, respectively, which were smaller than that reported in literatures. One factor that may affect the bladder volume is weight. In this study, the mean cat weight was $3.5\pm 1.4$ kg ranging from 1.9 to 5.8 kg. It is observed that cat 1 and cat 2, which have higher weight compared to the other cats (5.8 kg and 4.8 kg), have a high bladder volume ($22.0\pm 4.7$ ml and $13.4\pm 4.9$ ml). Meanwhile, the weight of the cat 3 is 4.8 kg, but the bladder volume is not high ($4.2\pm 3.1$ ml). However, regardless of whether the bladder volume is low or high, the proposed method could predict the urine leakage with a high success rate (94.2% in normal and 100% in OAB).

Anesthesia depth is another important factor that may affect the bladder volume^43^. Anesthesia can inhibit the central nervous system reflexes and increase the bladder volume. The goal in this study was to imitate natural awake condition. Hence, a low-dose of alpha-chloralose (50 mg/kg) was administrated to minimally suppress the autonomic bladder reflexes.

The proposed experimental setup for recording CMG trials, along with the identification of a leakage point, in alpha-chloralosed anesthetized cats offers a broader range of applicability compared to previous studies conducted under isovolumetric conditions^23,24^. This approach has been successfully tested on CMGs without NVC as well as in the presence of NVC, which could potentially simulate certain OAB models in humans.

The clinical application of the proposed method lies in the development of implantable sensor for measuring the bladder pressure. The proposed approach could potentially incorporate implantable pressure sensor^44–46^ for leakage prediction and control of bladder incontinence. However, there are a number of challenges facing artificial sensors such as invasiveness, artifacts from patient movement, abdominal pressure changes, and material biocompatibility. In previous work, we developed a method for estimating the bladder pressure/volume from neural activity recorded directly from the spinal cord gray matter neurons^47^. This technique represents a promising approach for adapting the proposed method to deal with clinical pathways.

### Supplementary Information

Supplementary Figure S1.

Supplementary Figure S2.

## Data Availability

All datasets obtained during the current study are available from the corresponding author on reasonable request.
