# Supplementary material for: Real-time prediction of bladder urine leakage using fuzzy inference system and dual Kalman filtering in cats
Source: Sci Rep. 2024 Feb 16;14:3879. doi: 10.1038/s41598-024-53629-5 (PMC10873426; doi:10.1038/s41598-024-53629-5)
Supplement: Supplementary file 1 — Supplementary Figure S1. [file 41598_2024_53629_MOESM1_ESM.pdf]

Real-time prediction of bladder urine leakage using fuzzy inference system and dual Kalman filtering in cats

(Results of the CMG test and leakage Prediction during bladder infusion with saline)

Amirhossein Qasemi[1], Alireza Aminian[1], and Abbas Erfanian[1]

[1]Department of Biomedical Engineering, School of electrical engineering, Iran Neural Technology Research Center, Iran University of Science and Technology (IUST). Correspondence and requests for materials should be addressed to A.E. (email: erfanian@iust.ac.ir)

Figure S1. The results of the CMG test including measure bladder pressure, time-frequency analysis of the bladder pressure using dual Kalman filtering, infused bladder volume, voided volume, and residual bladder volume during bladder infusion with saline, as well as leakage Prediction using fuzzy logic inference system during normal condition on 8 cats (52 trials with saline infusion in cats 1-8).

Cat1_Trial1_Normal

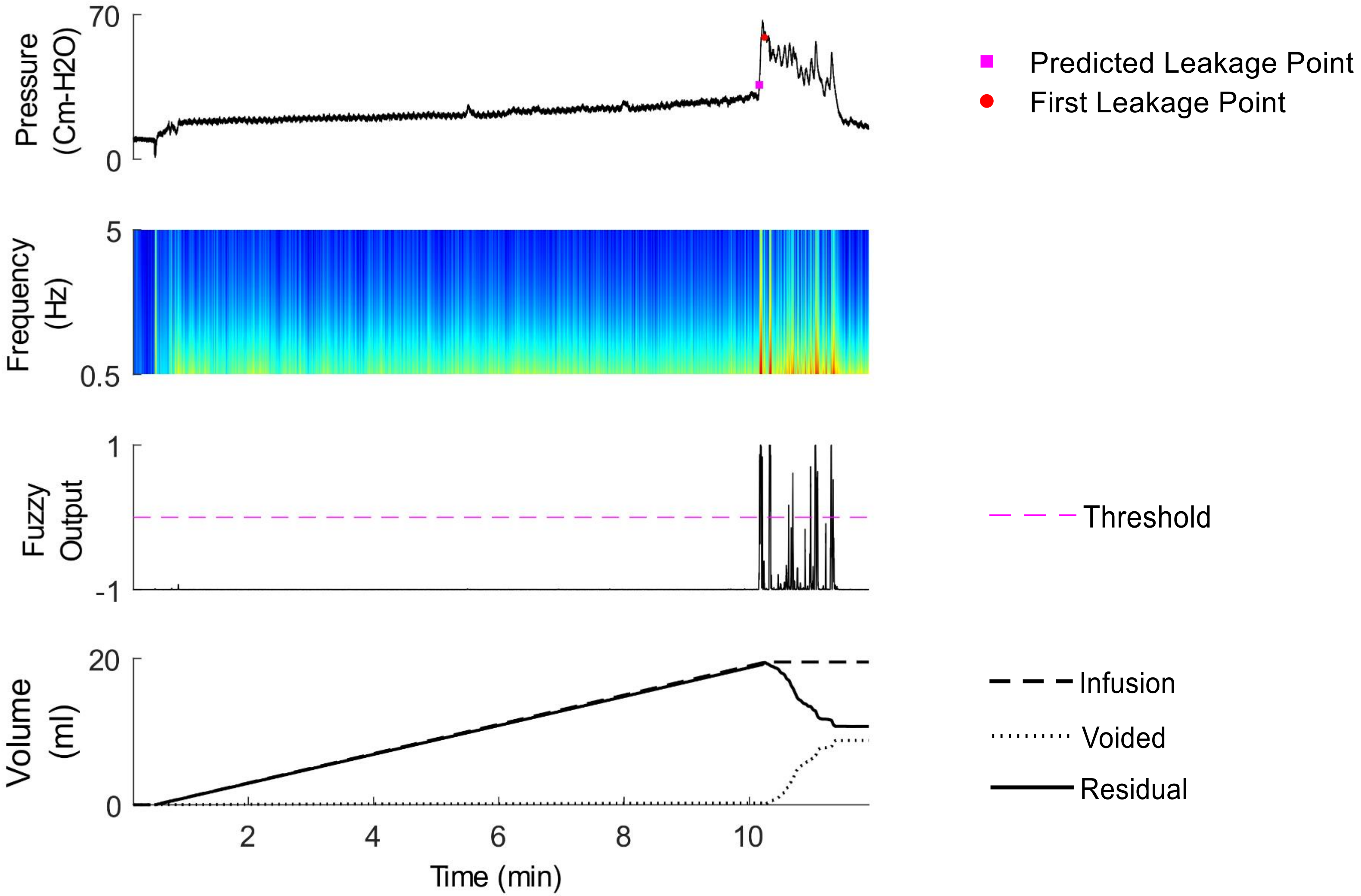

Gender = *male*, **Weight =** *5.8 kg*, **Infusion Rate =** *120 ml/h*, **Prediction Time =** *4.08 s*, **Delay Time =** *1.6 s*, **Pressure Increase =** *14.59 cmH2O*

Cat1_Trial2_Normal

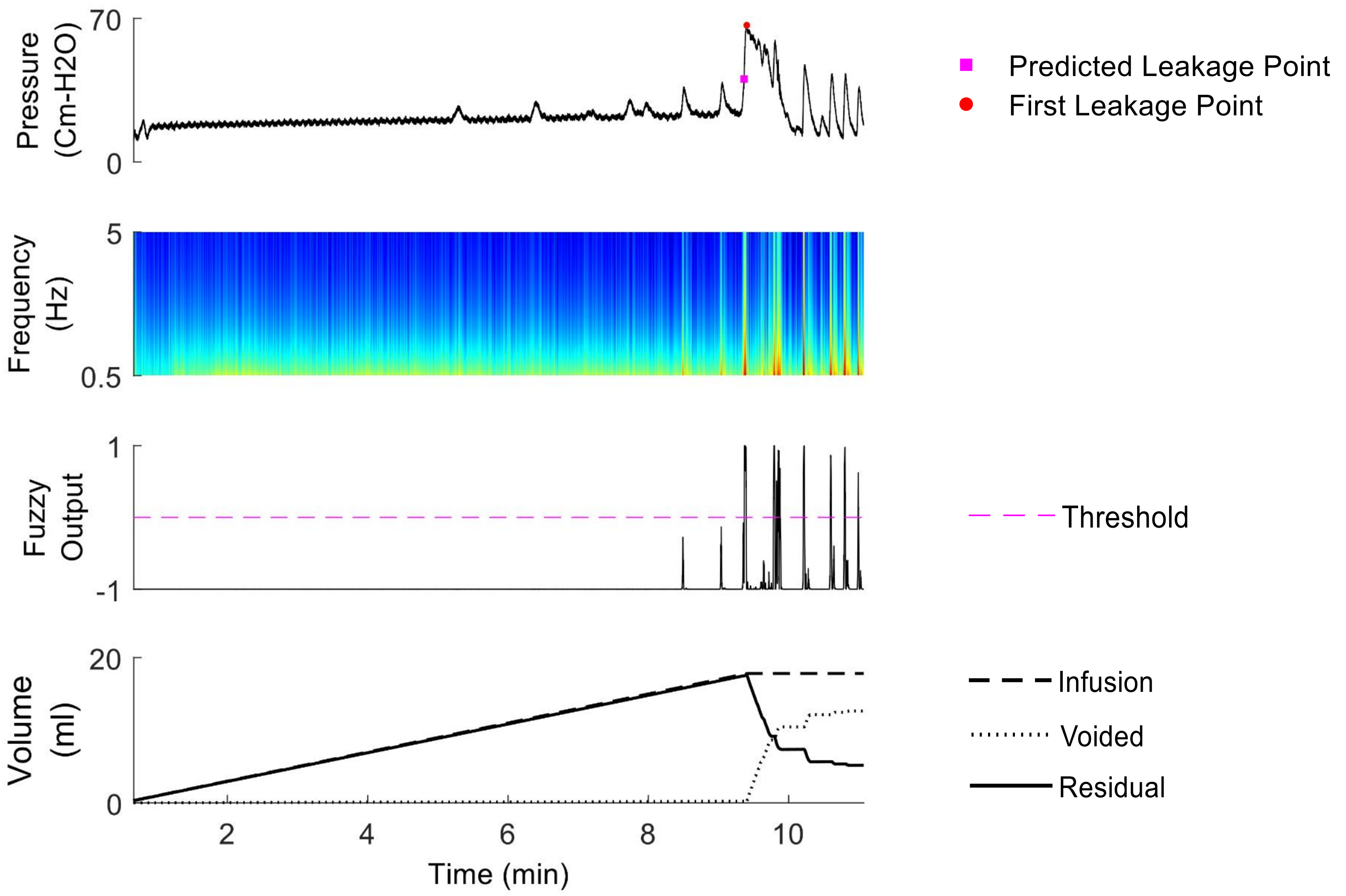

Gender = *male*, **Weight =** *5.8 kg* , **Infusion Rate =** *120 ml/h*, **Prediction Time =** *2.2s*, **Delay Time =** *1.7 s*, **Pressure Increase =** *14.88 cmH2O*

Cat1_Trial3_Normal

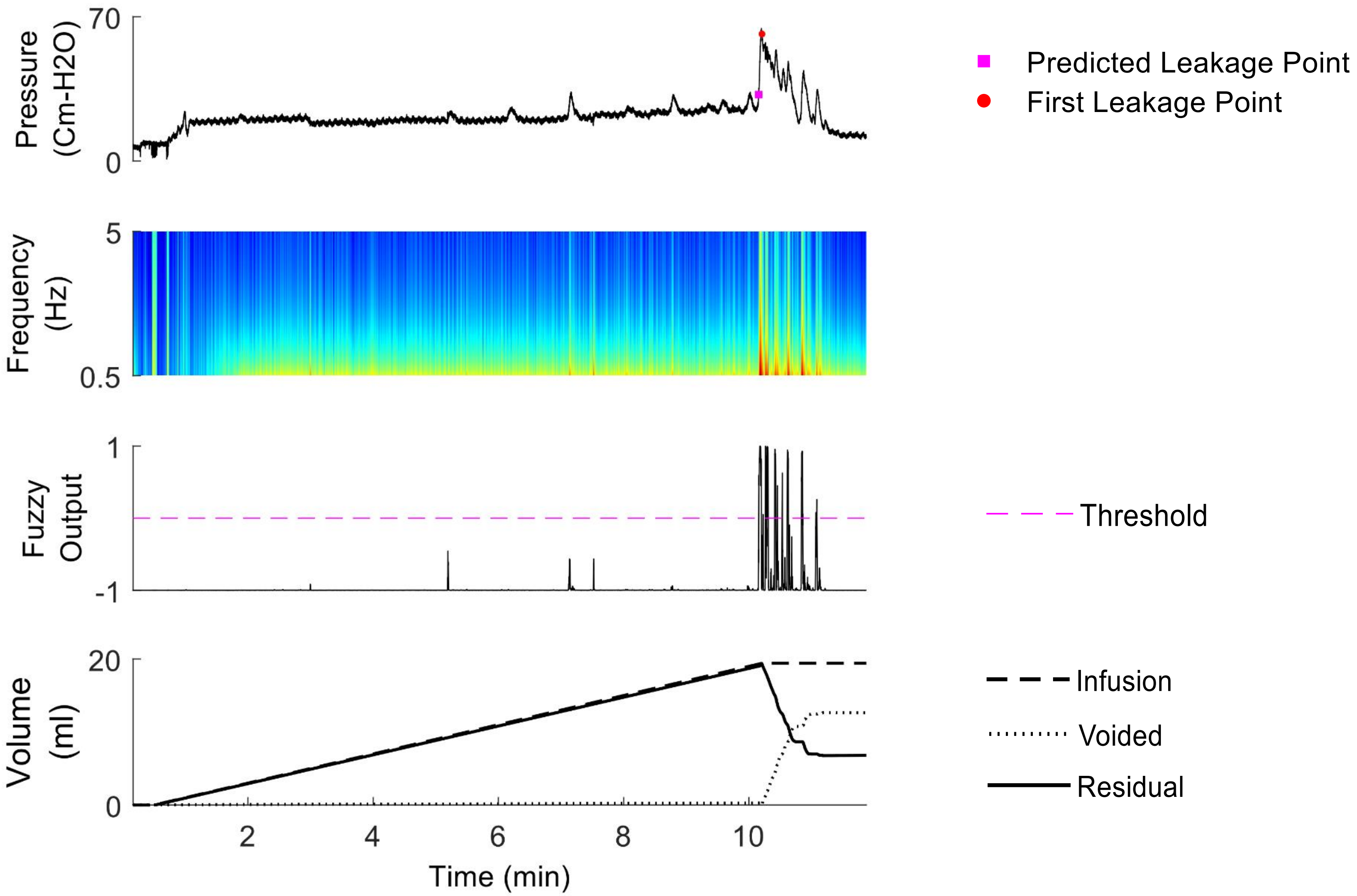

Gender = *male*, **Weight =** *5.8 kg*, **Infusion Rate =** *120 ml/h*, **Prediction Time =** *2.76 s*, **Delay Time =** *0.8 s*, **Pressure Increase =** *8.23 cmH2O*

Cat1_Trial4_Normal

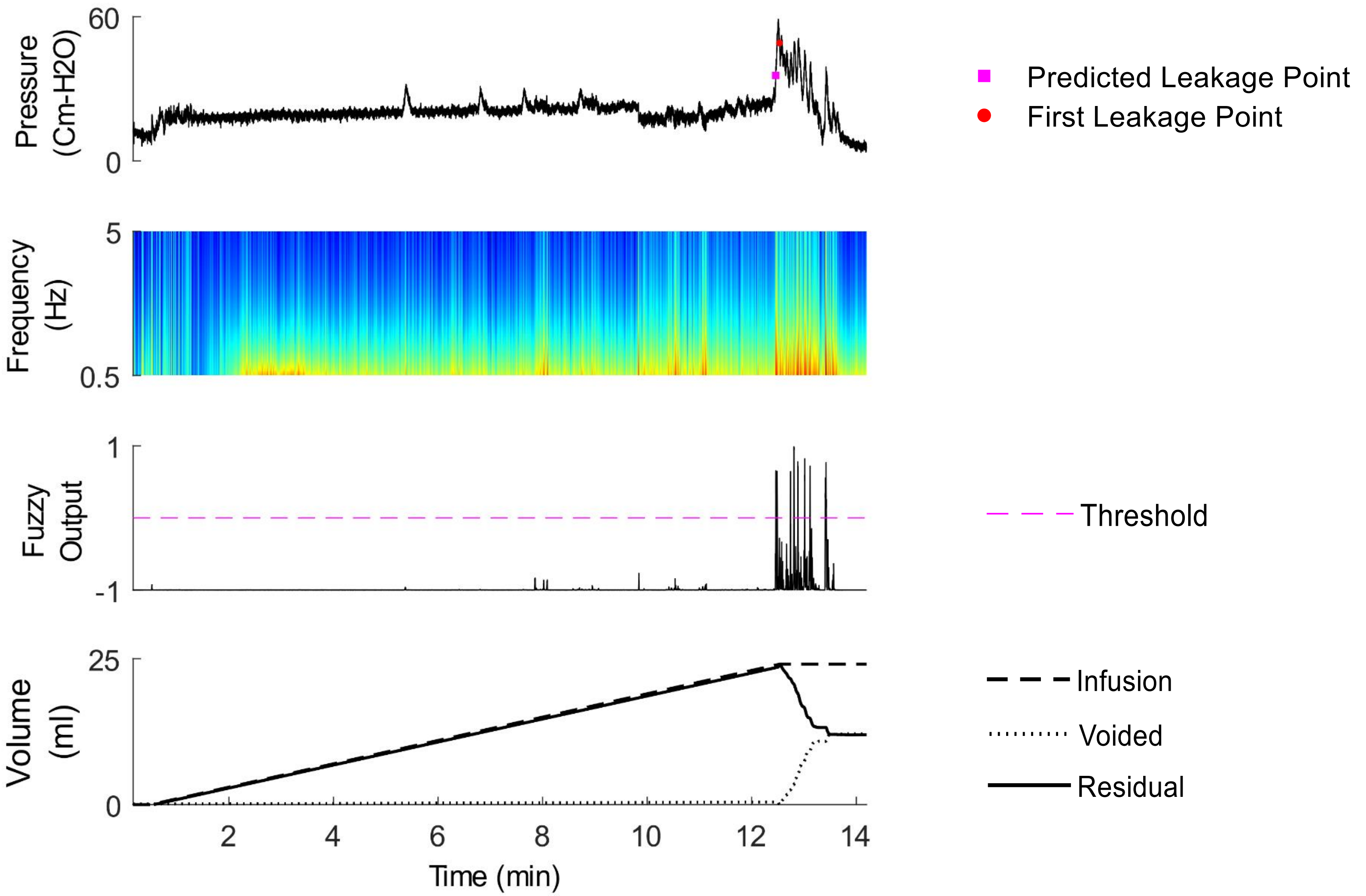

Gender = *male*, **Weight =** *5.8 kg*, **Infusion Rate =** *120 ml/h*, **Prediction Time =** *4.06 s*, **Delay Time =** *0.996s*, **Pressure Increase =** *14.65 cmH2O*

Cat1_Trial5_Normal

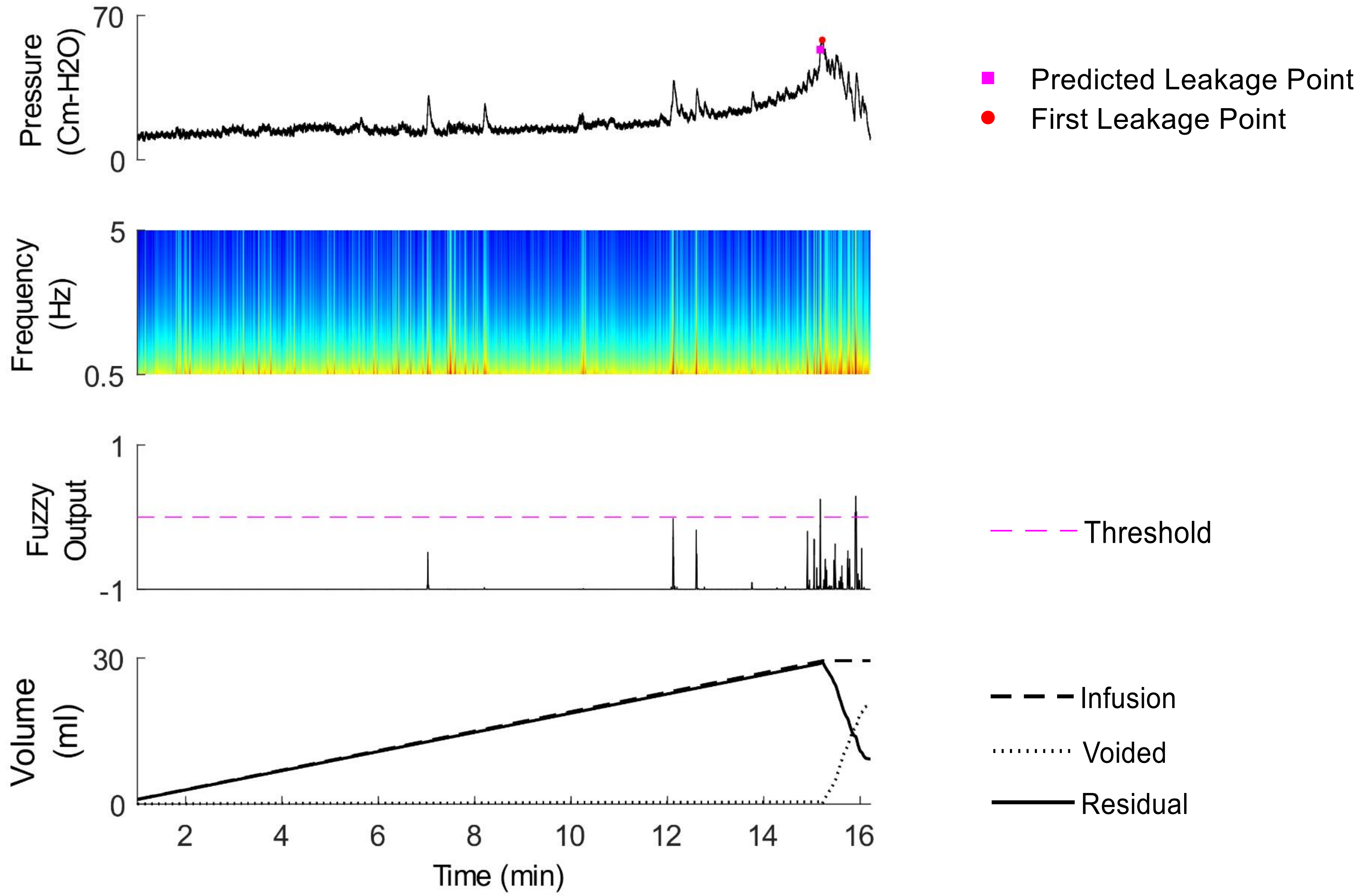

Gender = *male*, **Weight =** *5.8 kg*, **Infusion Rate =** *120 ml/h*, **Prediction Time =** *2.48 s*, **Delay Time =** *3.76 s*, **Pressure Increase =** *14.045 cmH2O*

Cat2_Trial1_Normal

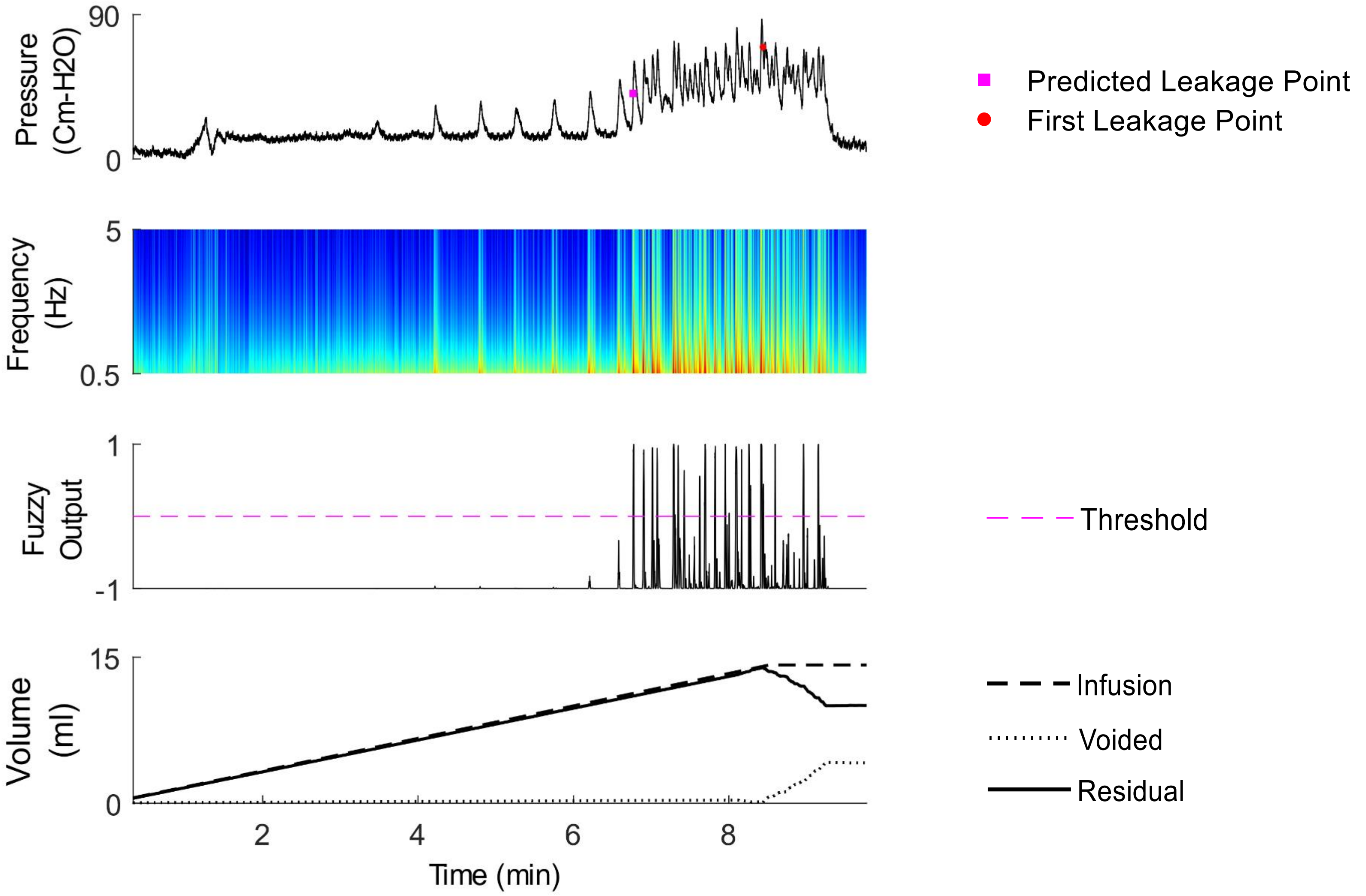

Gender = *male*, **Weight =** *4.8 kg*, **Infusion Rate =** *120 ml/h*, **Prediction Time =** *100.26 s*, **Delay Time =** *1.92 s*, **Pressure Increase =** *20.45 cmH2O*

Cat2_Trial2_Normal

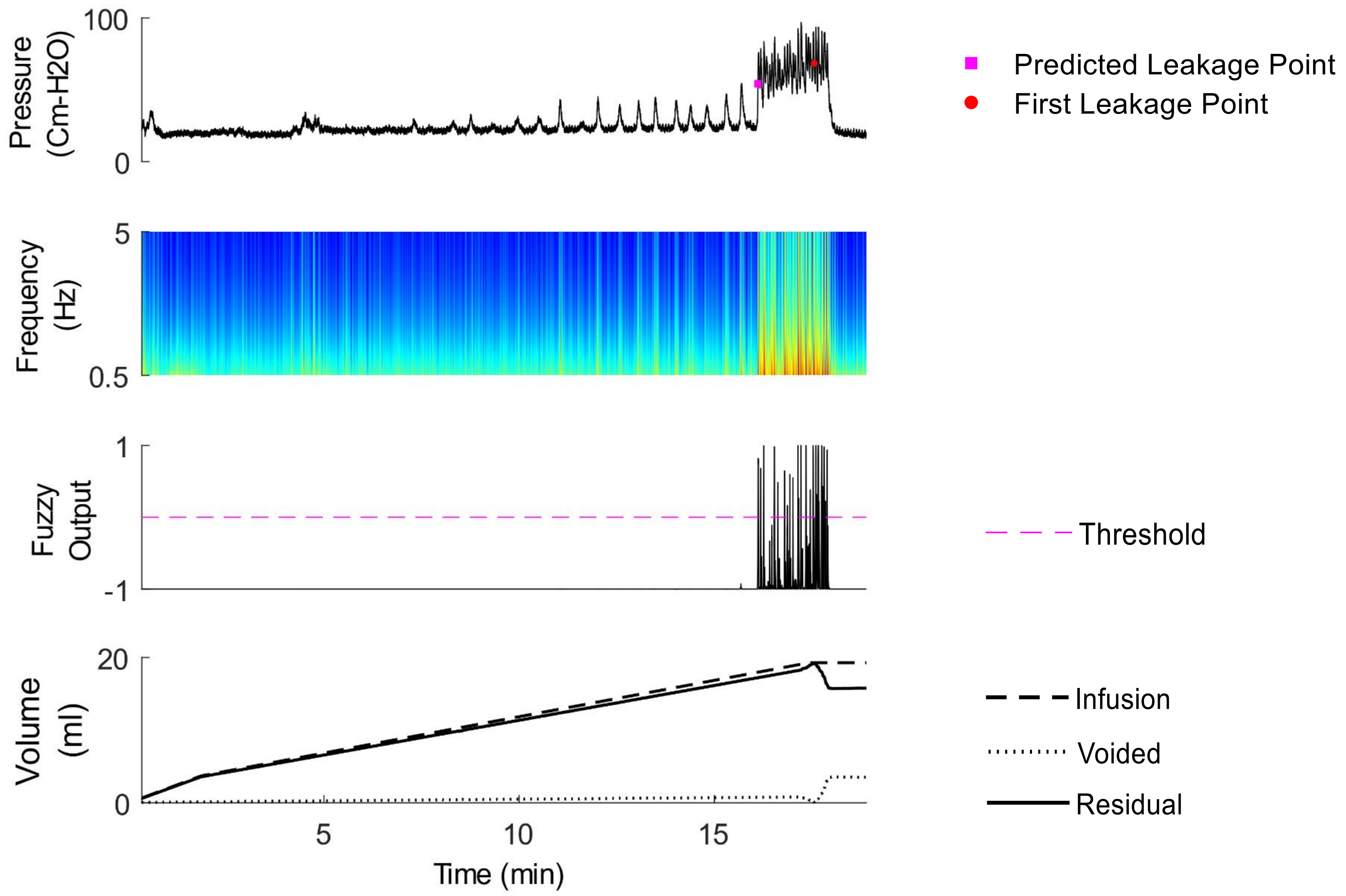

Gender = *male*, **Weight =** *4.8 kg*, **Infusion Rate =** *120 ml/h*, **Prediction Time =** *86.52 s*, **Delay Time =** *1.52 s*, **Pressure Increase =** *28.45 cmH2O*

Cat2_Trial3_Normal

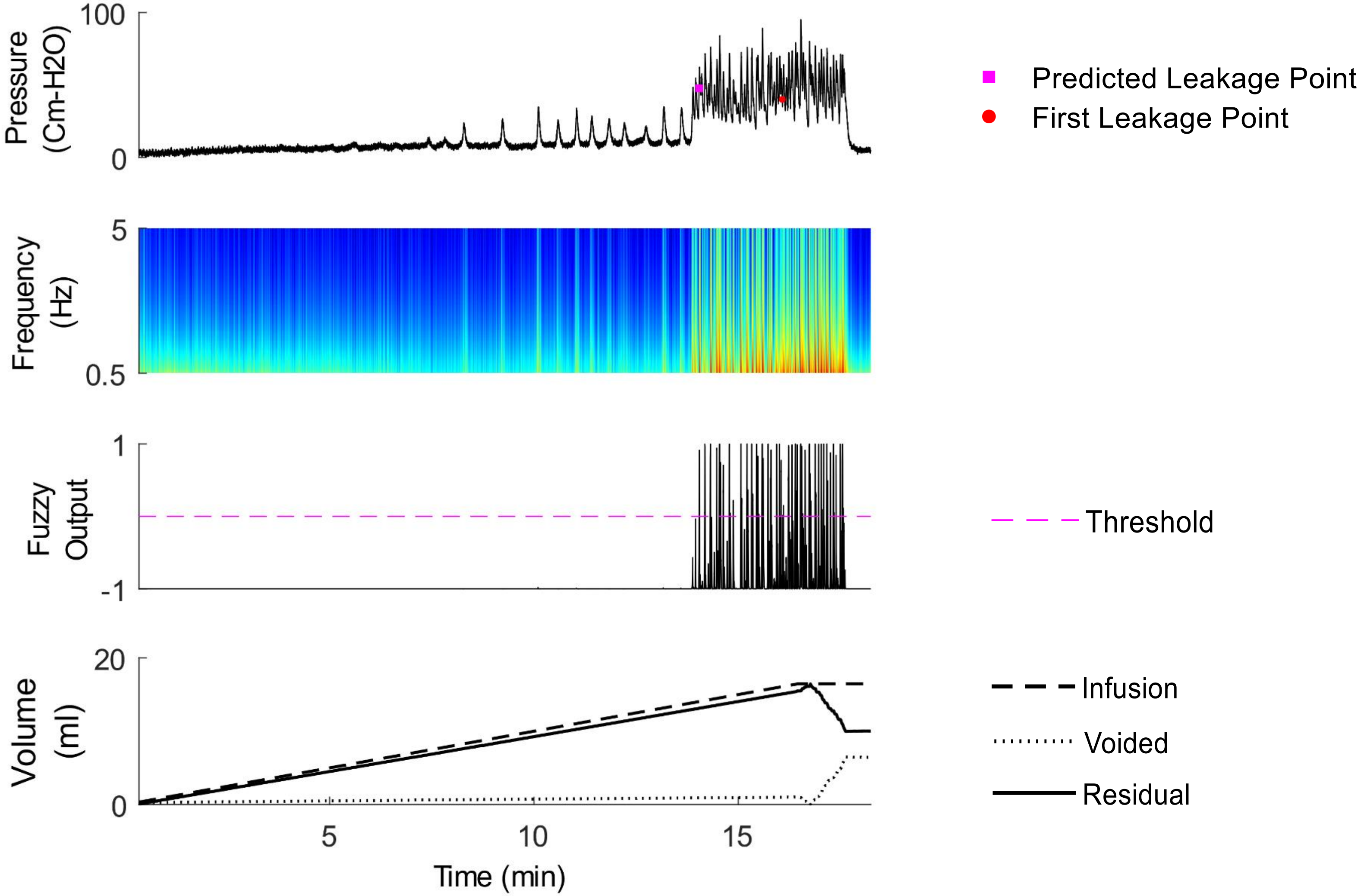

Gender = *male*, **Weight =** *4.8 kg*, **Infusion Rate =** *120 ml/h*, **Prediction Time =** *122.07 s*, **Delay Time =** *0.78 s*, **Pressure Increase =** *20.67 cmH2O*

Cat2_Trial4_Normal

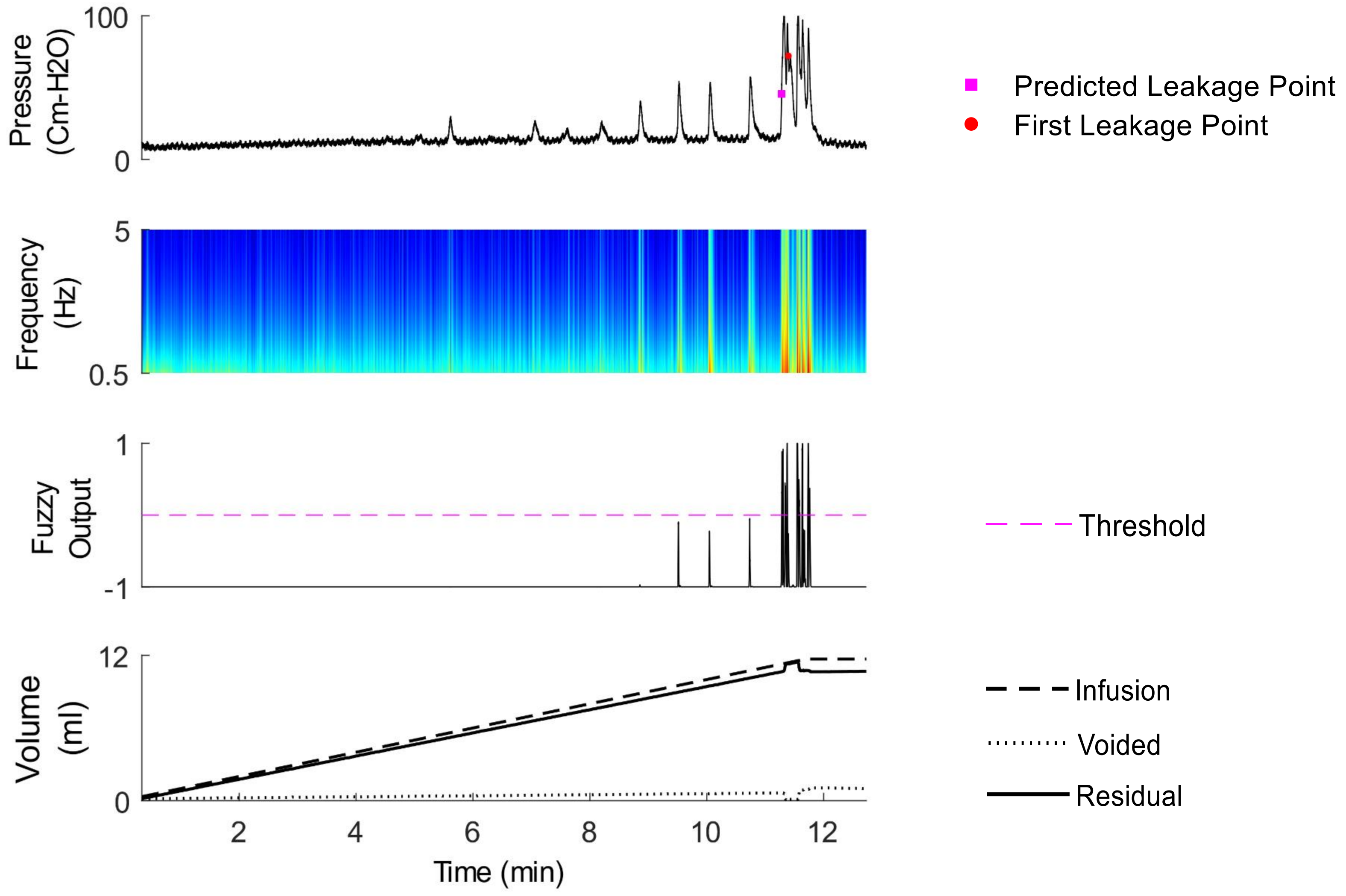

Gender = *male*, **Weight =** *4.8 kg*, **Infusion Rate =** *120 ml/h*, **Prediction Time =** *4.92 s*, **Delay Time =** *1.64 s*, **Pressure Increase =** *28.89 cmH2O*

Cat2_Trial5_Normal

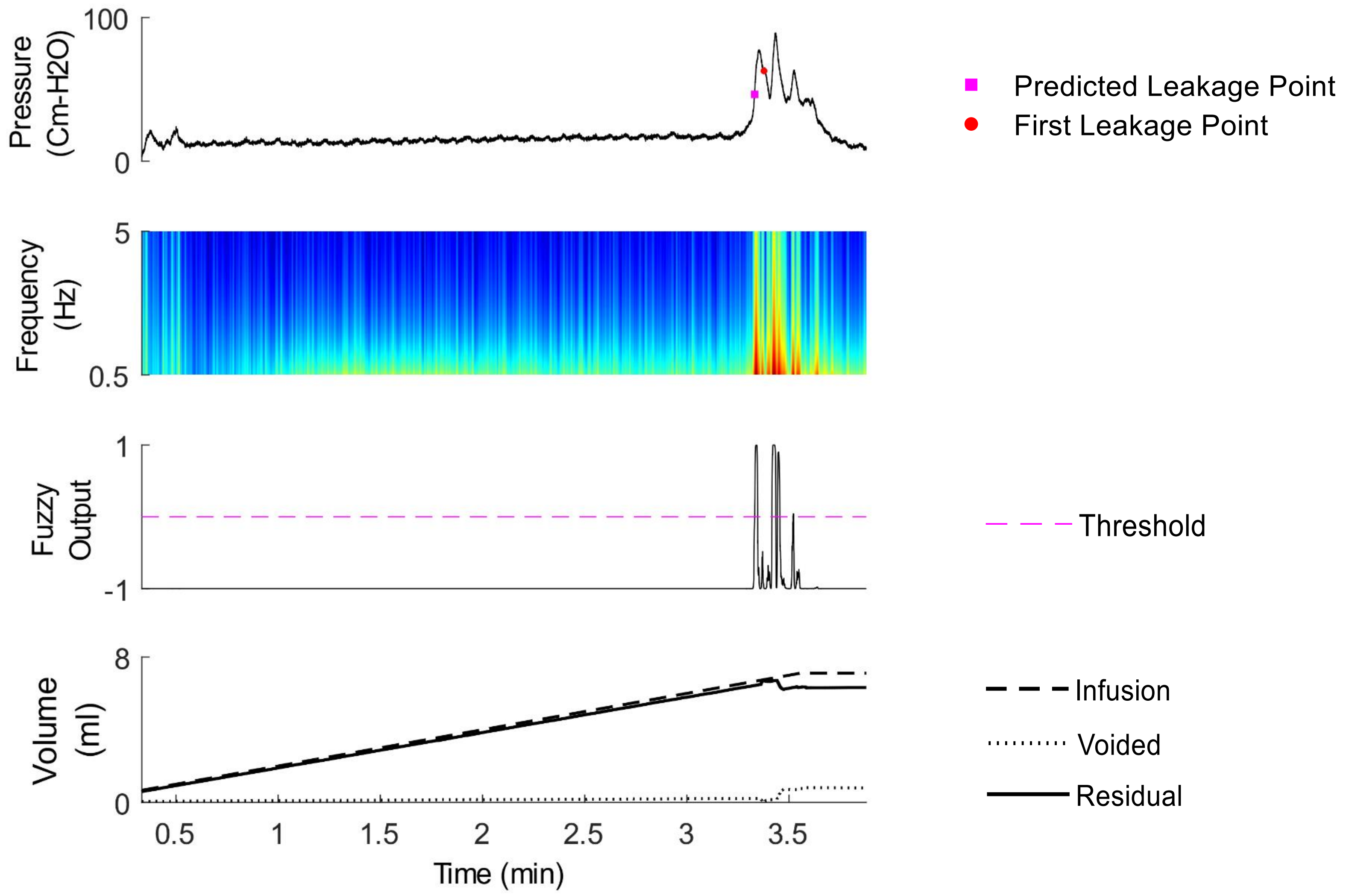

Gender = *male*, **Weight =** *4.8 kg*, , **Infusion Rate =** *120 ml/h*, **Prediction Time =** *2.7 s*, **Delay Time =** *1.34 s*, **Pressure Increase =** *23.05 cmH2O*

Cat3_Trial1_Normal

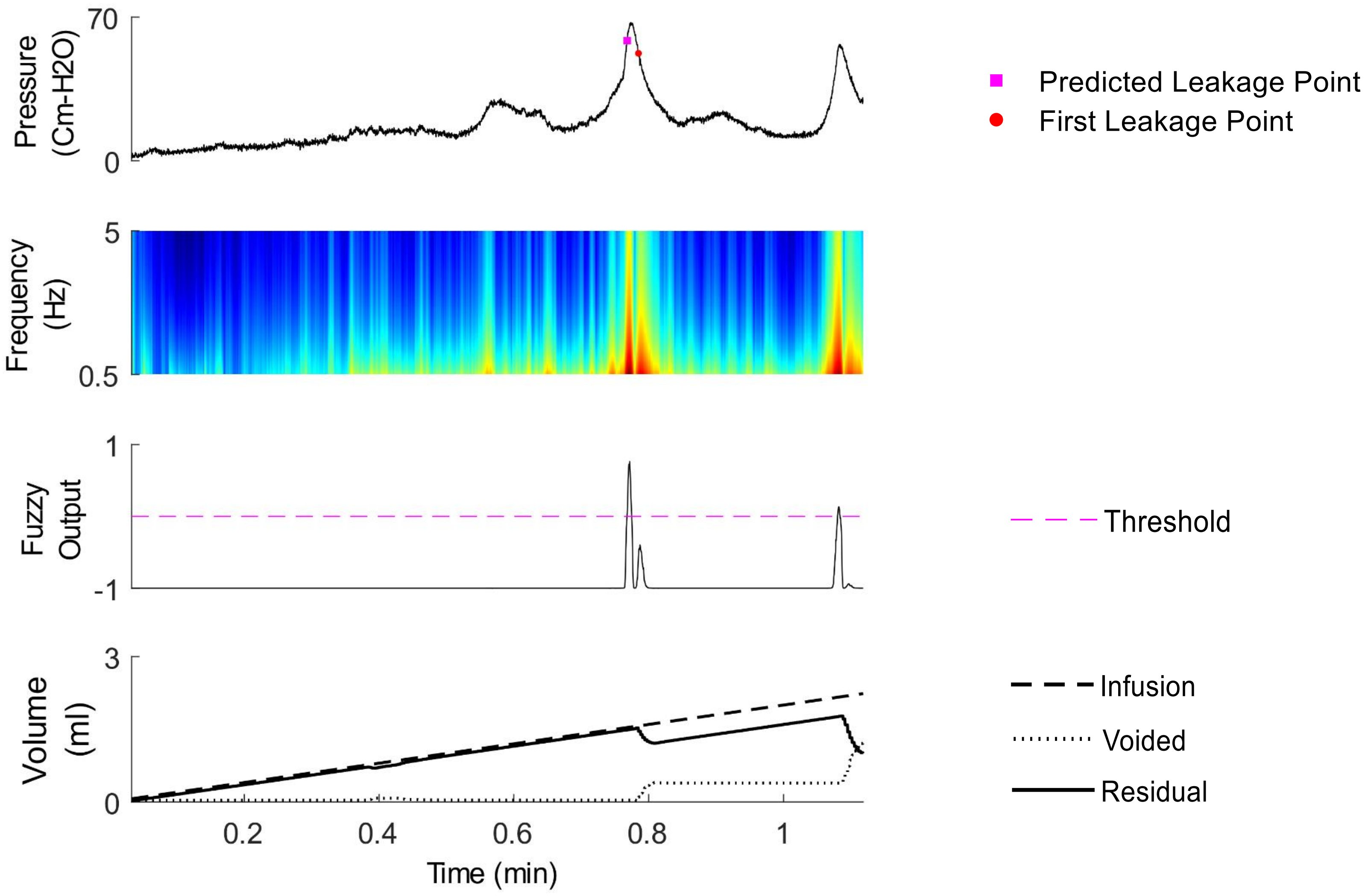

Gender = *male*, **Weight =** *4.8 kg*, , **Infusion Rate =** *120 ml/h*, **Prediction Time =** *1.34 s*, **Delay Time =** *2.12 s*, **Pressure Increase =** *25.4 cmH2O*

Cat3_Trial2_Normal

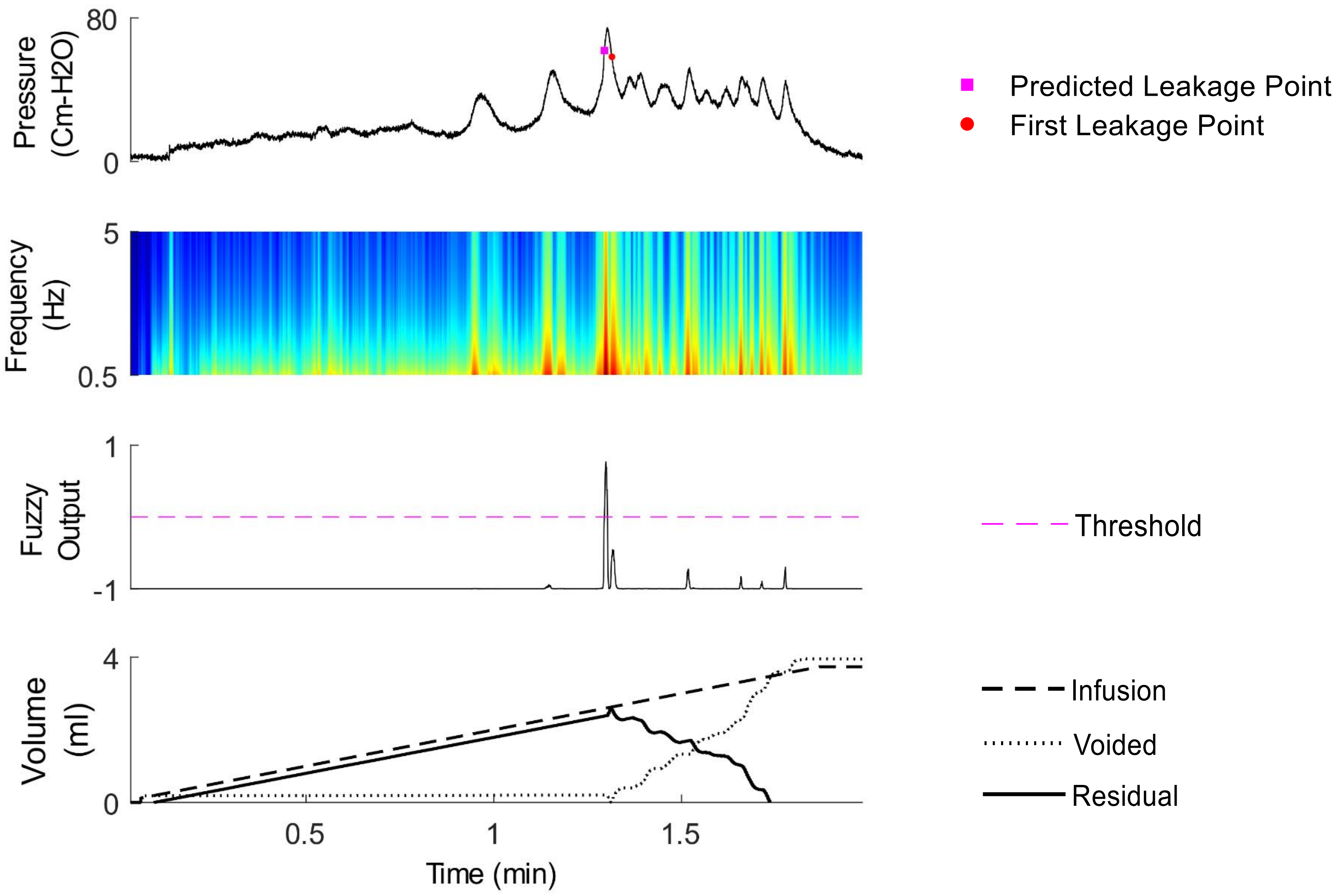

Gender = *male*, **Weight =** *4.8 kg*, , **Infusion Rate =** *120 ml/h*, **Prediction Time =** *1.04 s*, **Delay Time =** *1.53 s*, **Pressure Increase =** *15.18 cmH2O*

Cat3_Trial3_Normal

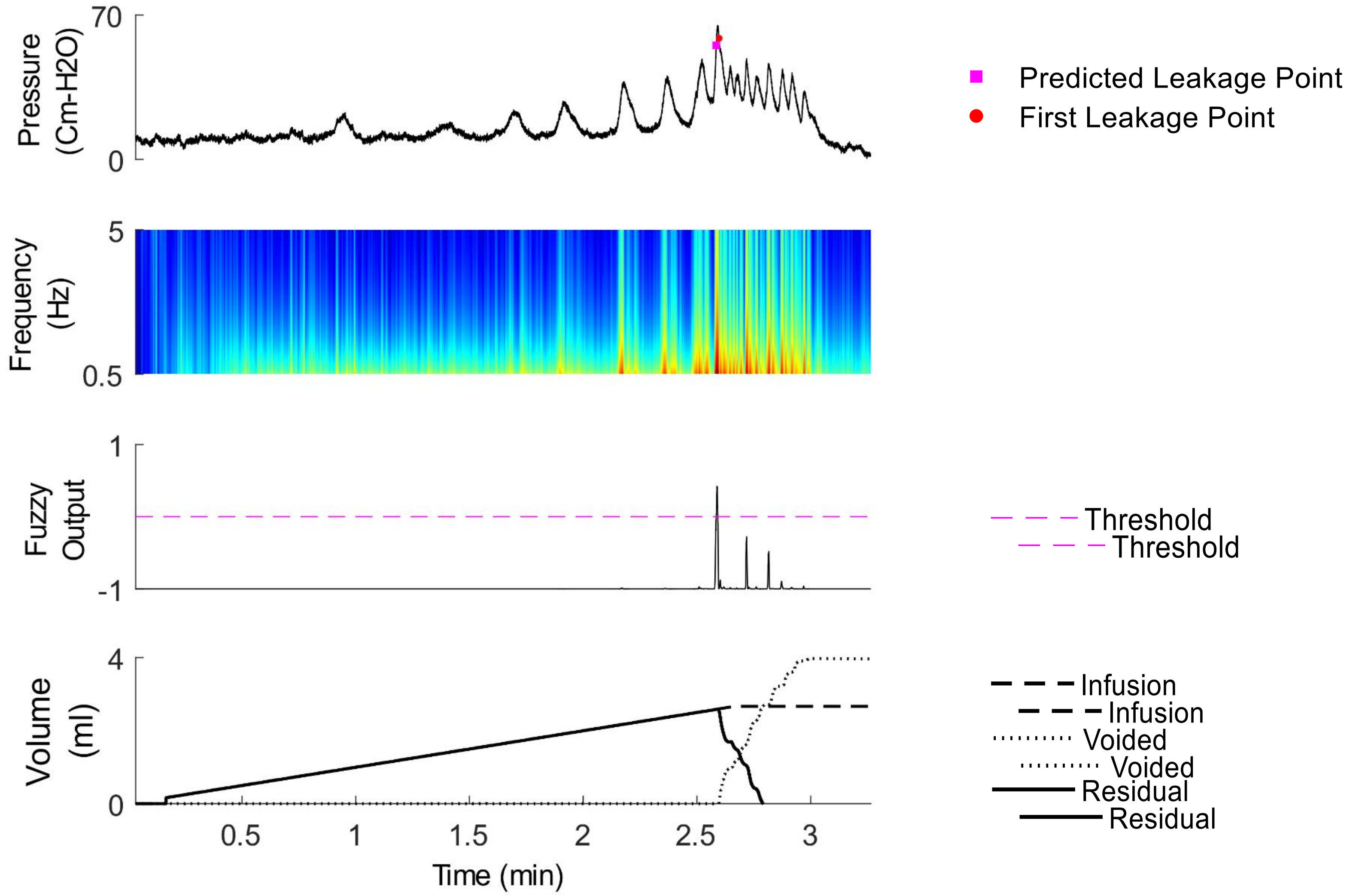

Gender = *male*, **Weight** *= 4.8 kg*, , **Infusion Rate =** *120 ml/h*, **Prediction Time =** *3.06 s*, **Delay Time =** *1.62 s*, **Pressure Increase =** *20.45 cmH2O*

Cat3_Trial4_Normal

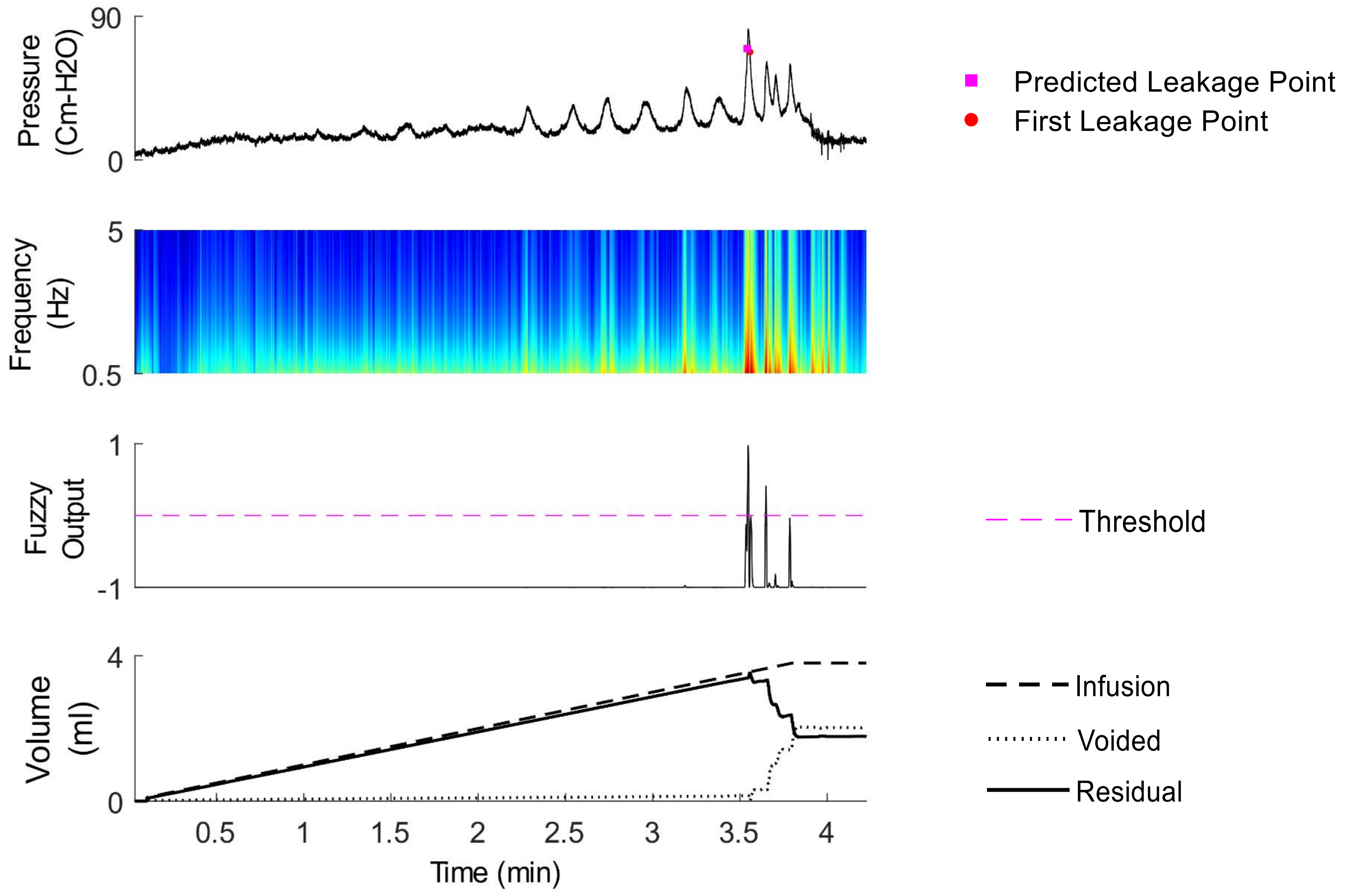

Gender = *male*, **Weight** *= 4.8 kg*, , **Infusion Rate =** *120 ml/h*, **Prediction Time =** *1.55 s*, **Delay Time =** *3.9 s*, **Pressure Increase =** *33.56 cmH2O*

Cat3_Trial5_Normal

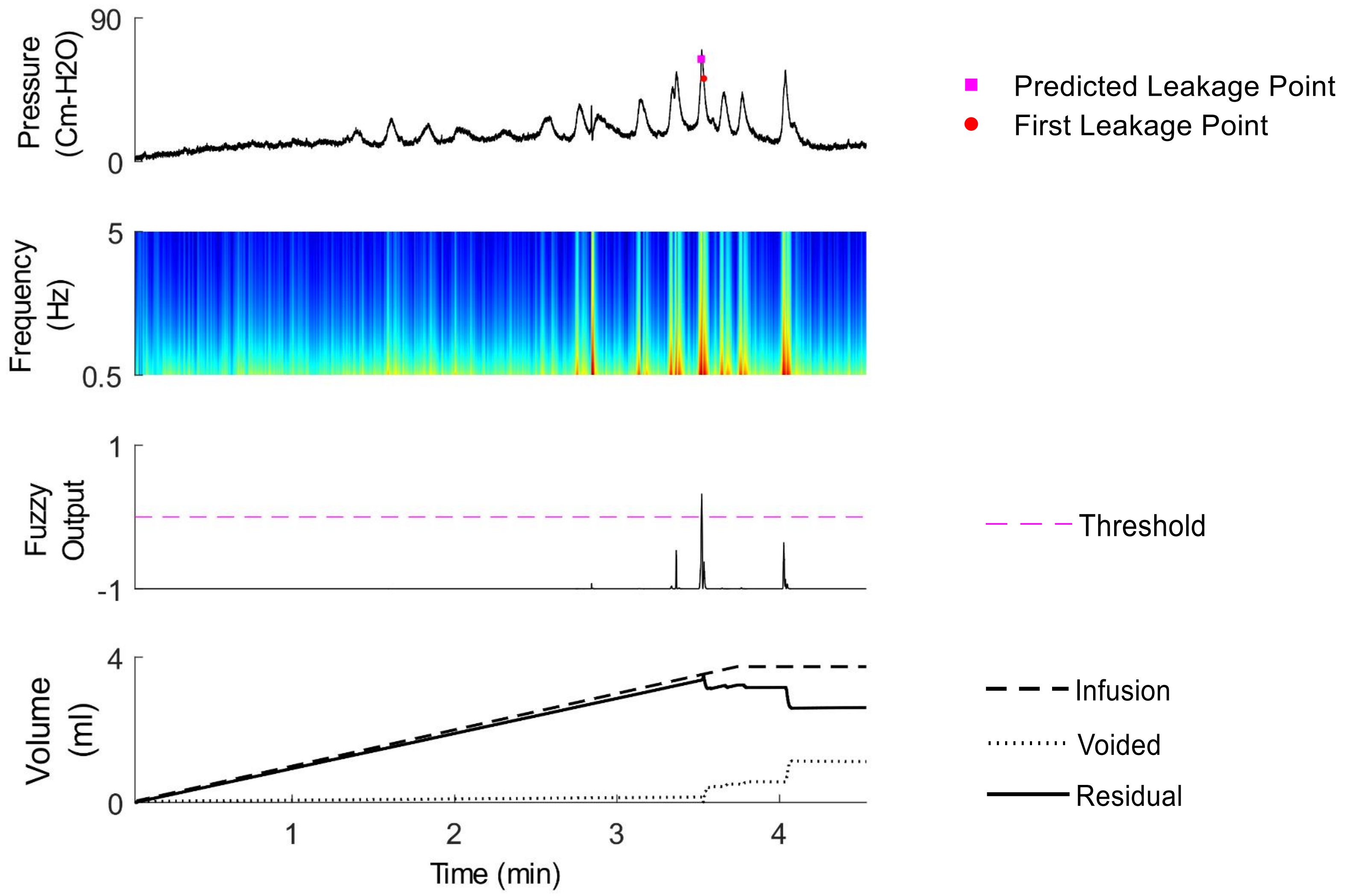

Gender = *male*, **Weight** *= 4.8 kg*, , **Infusion Rate =** *120 ml/h*, **Prediction Time =** *1.32 s*, **Delay Time =** *1.62 s*, **Pressure Increase =** *30 cmH2O*

Cat4_Trial1_Normal

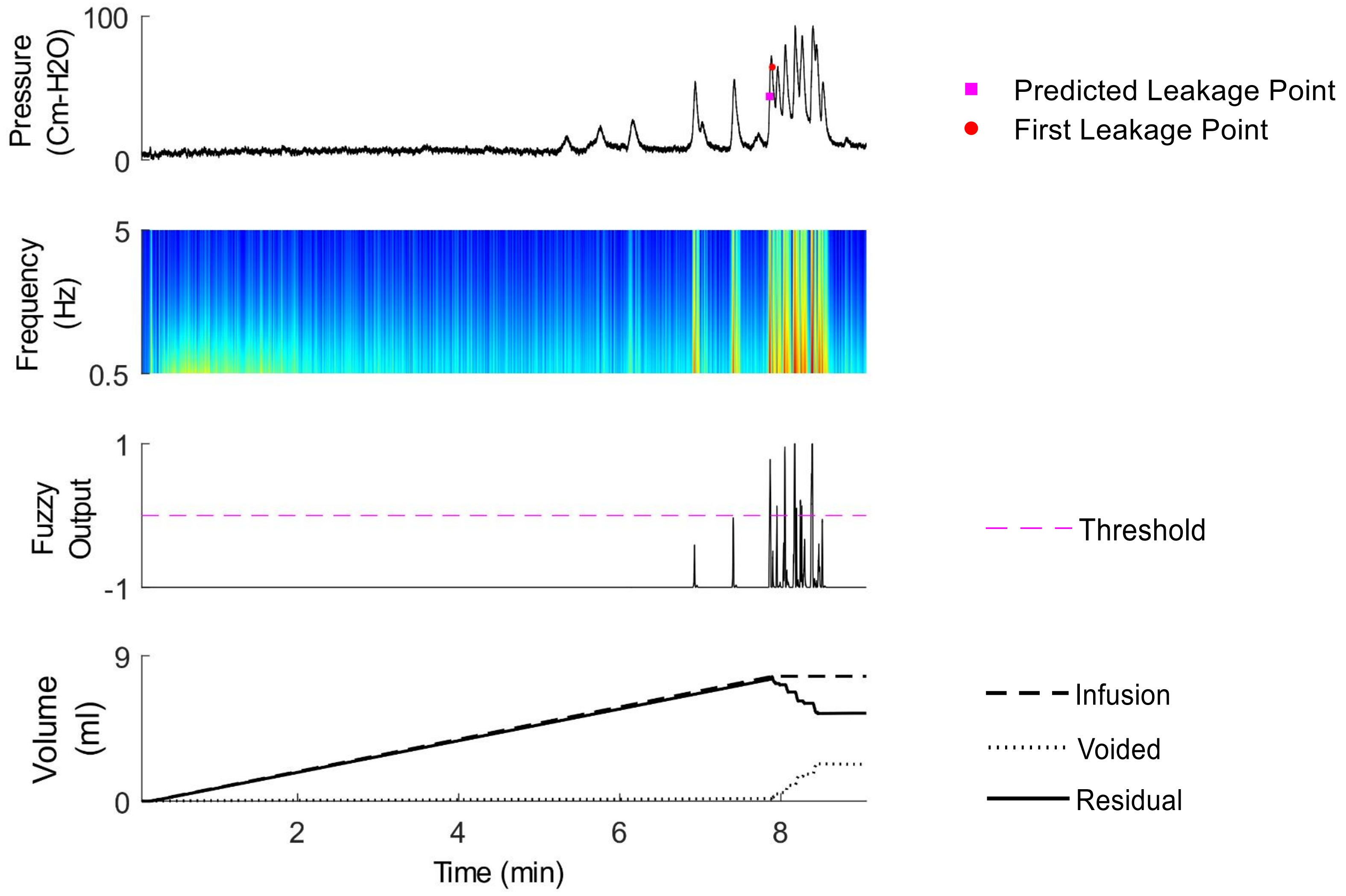

Gender = *male*, **Weight** *= 3.74 kg*, **Infusion Rate =** *60 ml/h*, **Prediction Time =** *2.06 s*, **Delay Time =** *0.86 s*, **Pressure Increase =** *27.35 cmH2O*

Cat4_Trial2_Normal

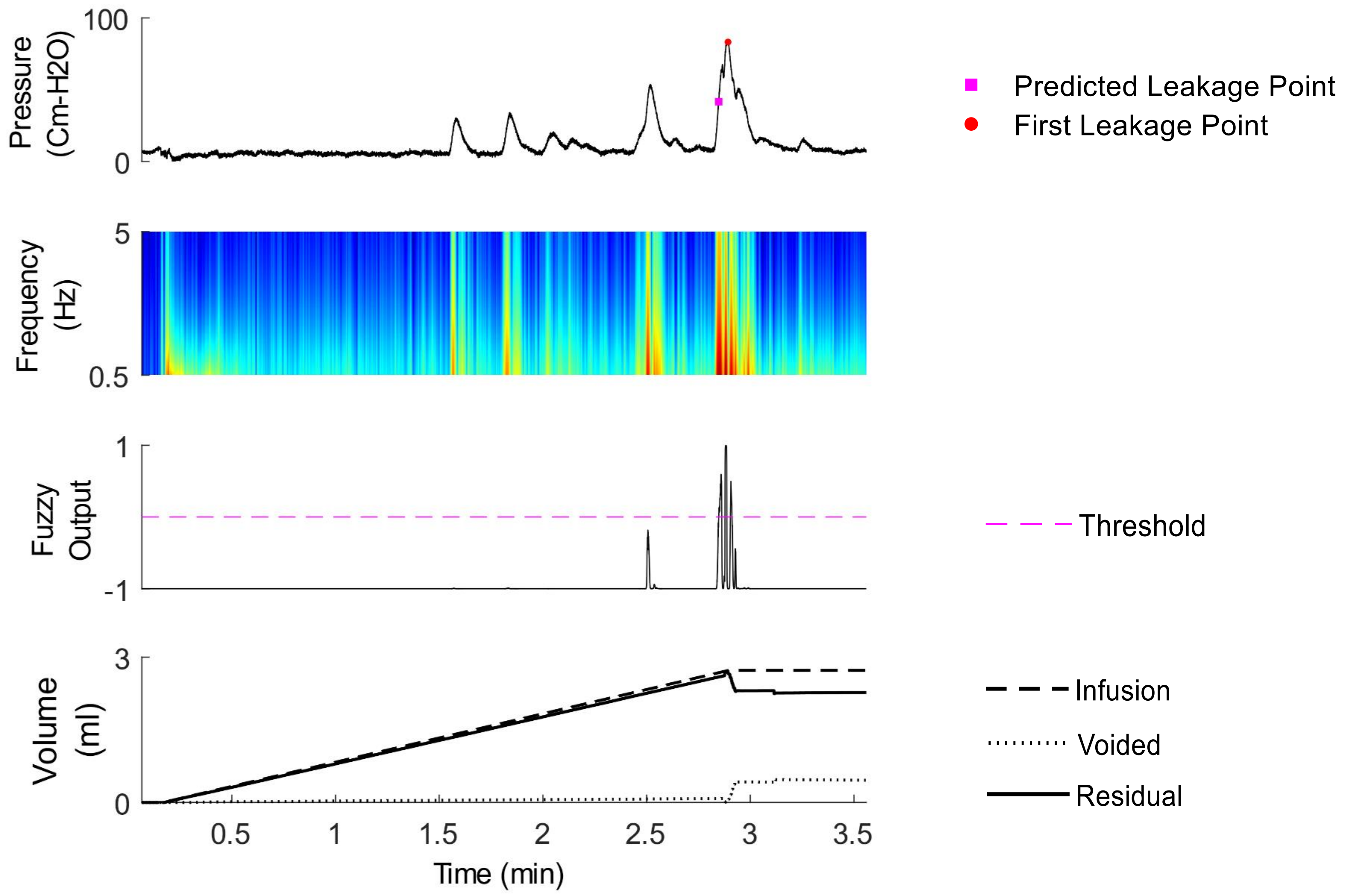

Gender = *male*, **Weight =** *3.74 kg*, **Infusion Rate =** *60 ml/h*, **Prediction Time =** *2.7 s*, **Delay Time =** *1.18 s*, **Pressure Increase =** *31.41 cmH2O*

Cat4_Trial3_Normal

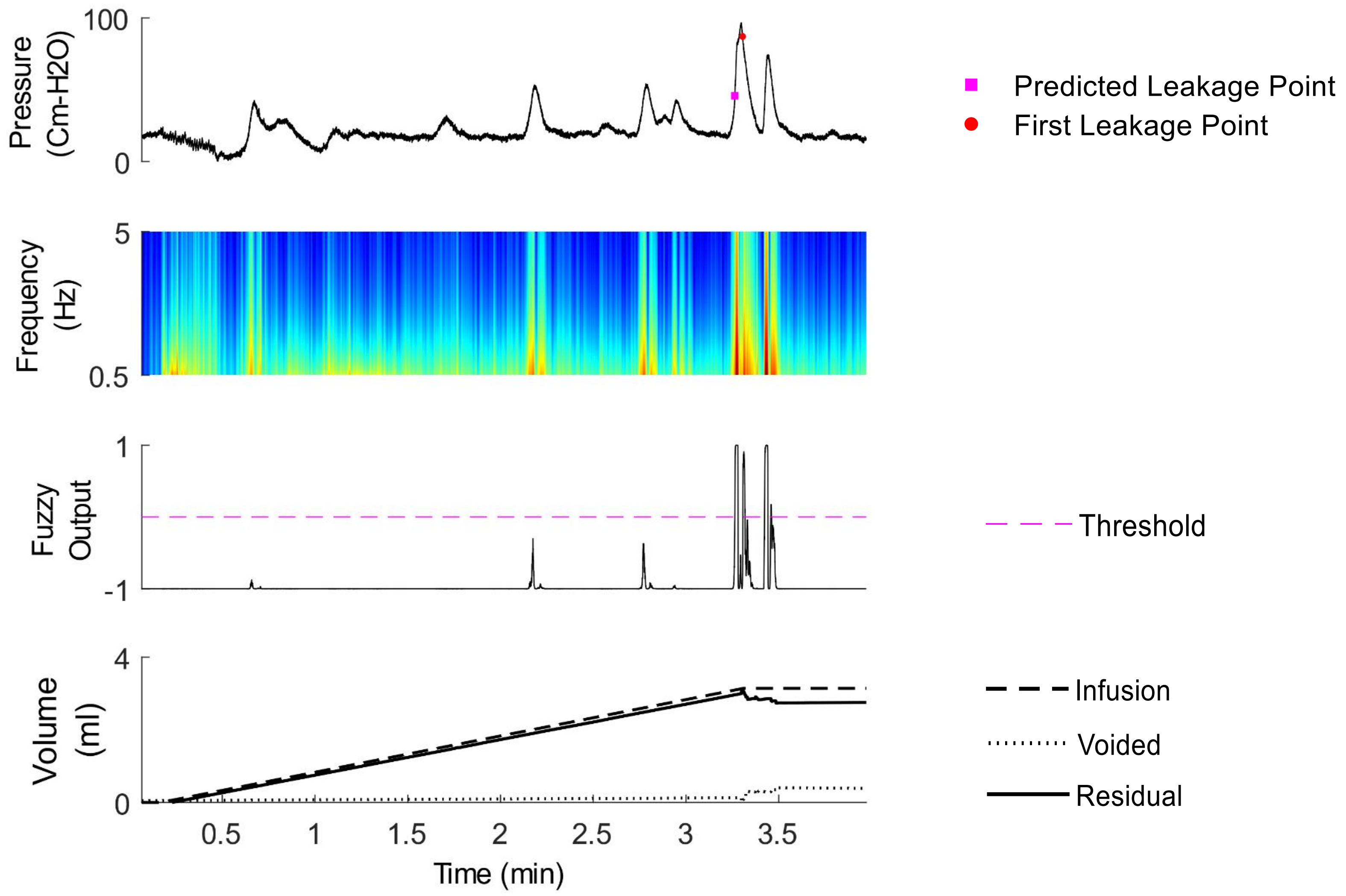

Gender = *male*, **Weight** *= 3.74 kg*, **Infusion Rate =** ***60 ml/h***, **Prediction Time =** ***2.52 s***, **Delay Time =** ***1.68 s***, **Pressure Increase =** ***27.26 cmH2O***

Cat4_Trial4_Normal

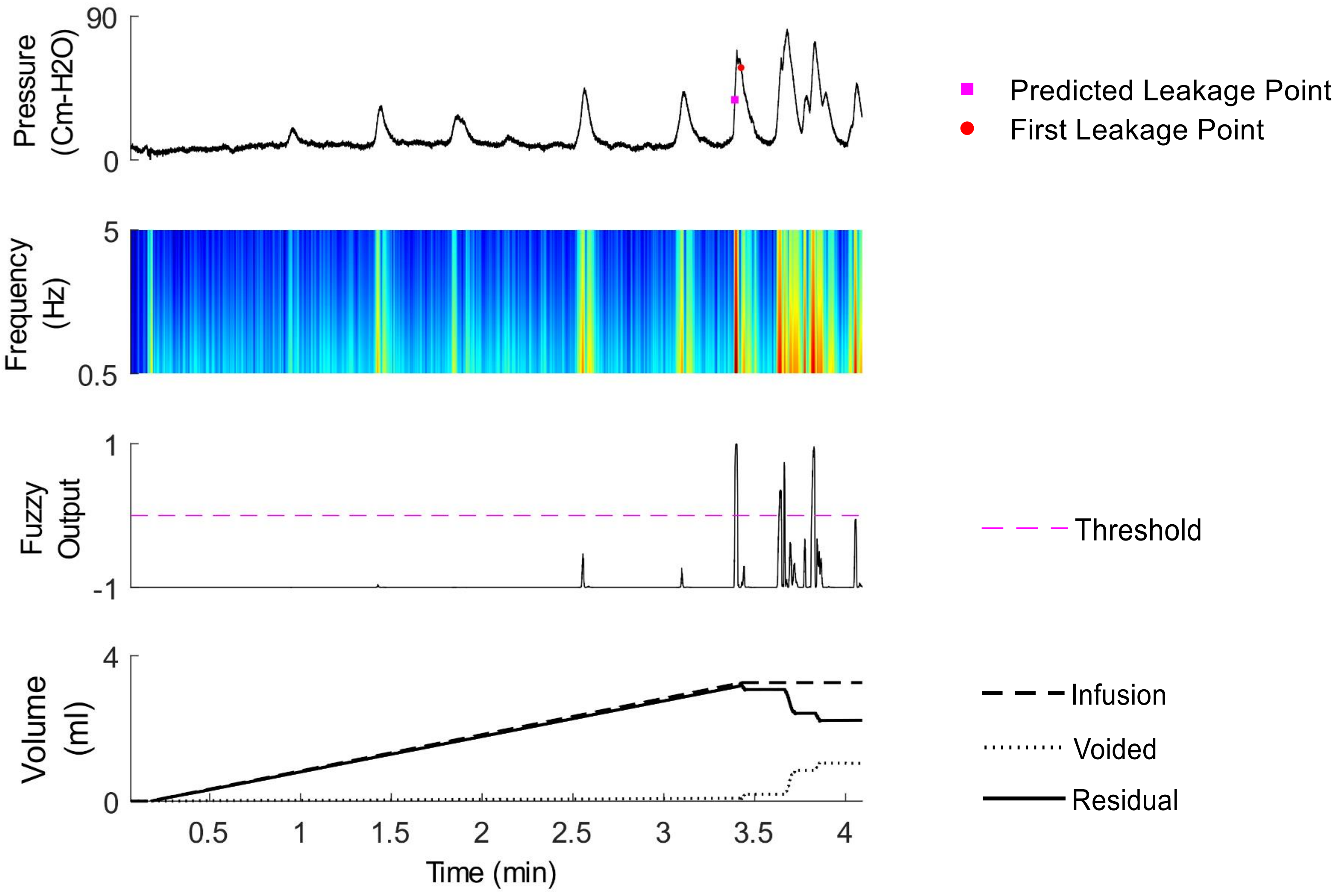

Gender = *male*, **Weight =** *3.74 kg*, **Infusion Rate =** *60 ml/h*, **Prediction Time =** *2.08 s*, **Delay Time =** *0.96 s*, **Pressure Increase =** *24.31 cmH2O*

Cat4_Trial5_Normal

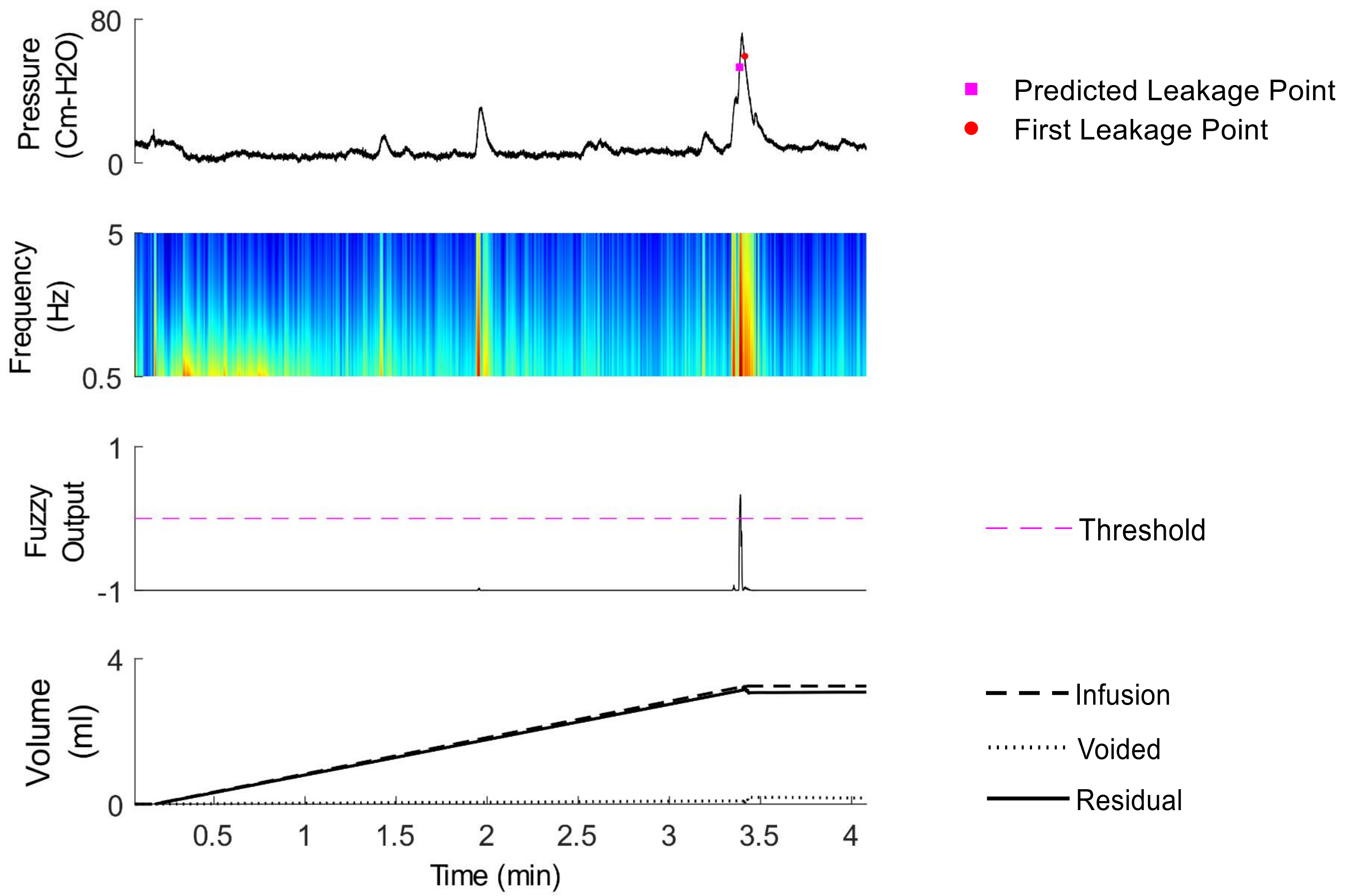

Gender = *male*, **Weight =** *3.74 kg*, **Infusion Rate =** *60 ml/h*, **Prediction Time =** *1.74 s*, **Delay Time =** *3 s*, **Pressure Increase =** *44.42 cmH2O*

Cat5_Trial1_Normal

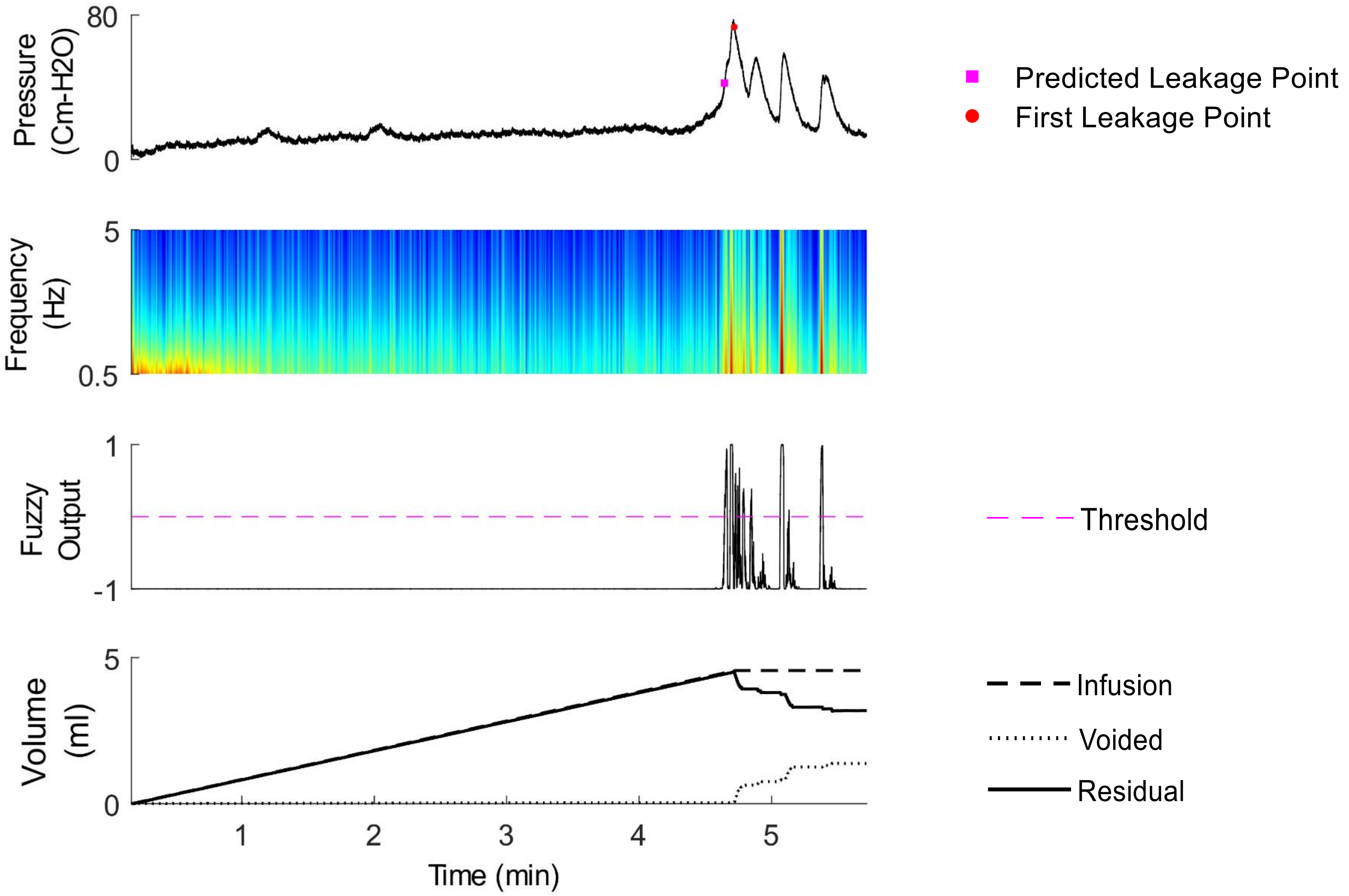

Gender = *male*, **Weight =** *2.8 kg*, **Infusion Rate =** *120 ml/h*, **Prediction Time =** *4.36 s*, **Delay Time =** *2.22 s*, **Pressure Increase =** *13.41 cmH2O*

Cat5_Trial2_Normal

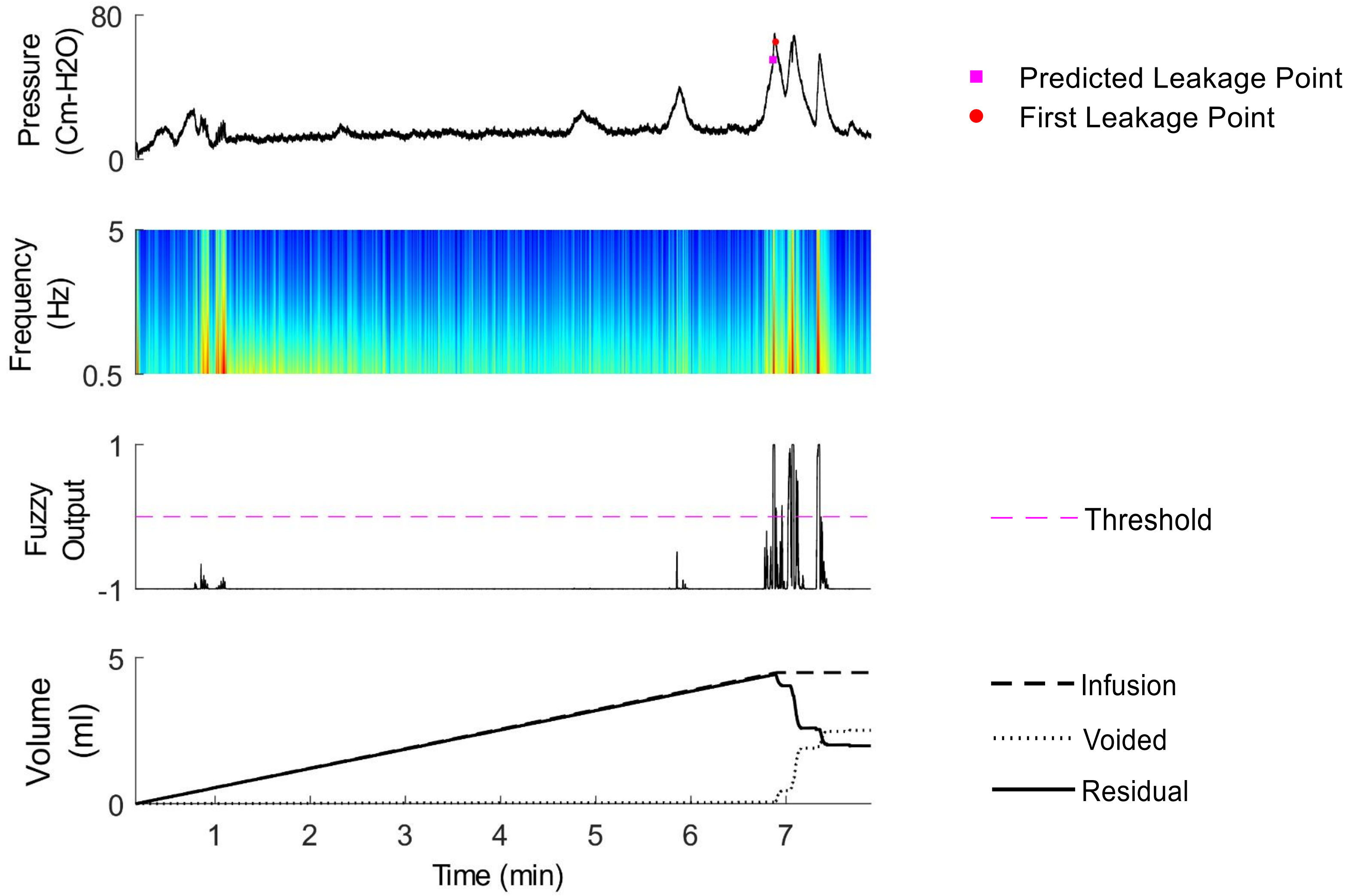

Gender = *male*, **Weight =** *2.8 kg*, **Infusion Rate =** *120 ml/h*, **Prediction Time =** *1.7 s*, **Delay Time =** *6.6 s*, **Pressure Increase =** *32.89 cmH2O*

Cat5_Trial3_Normal

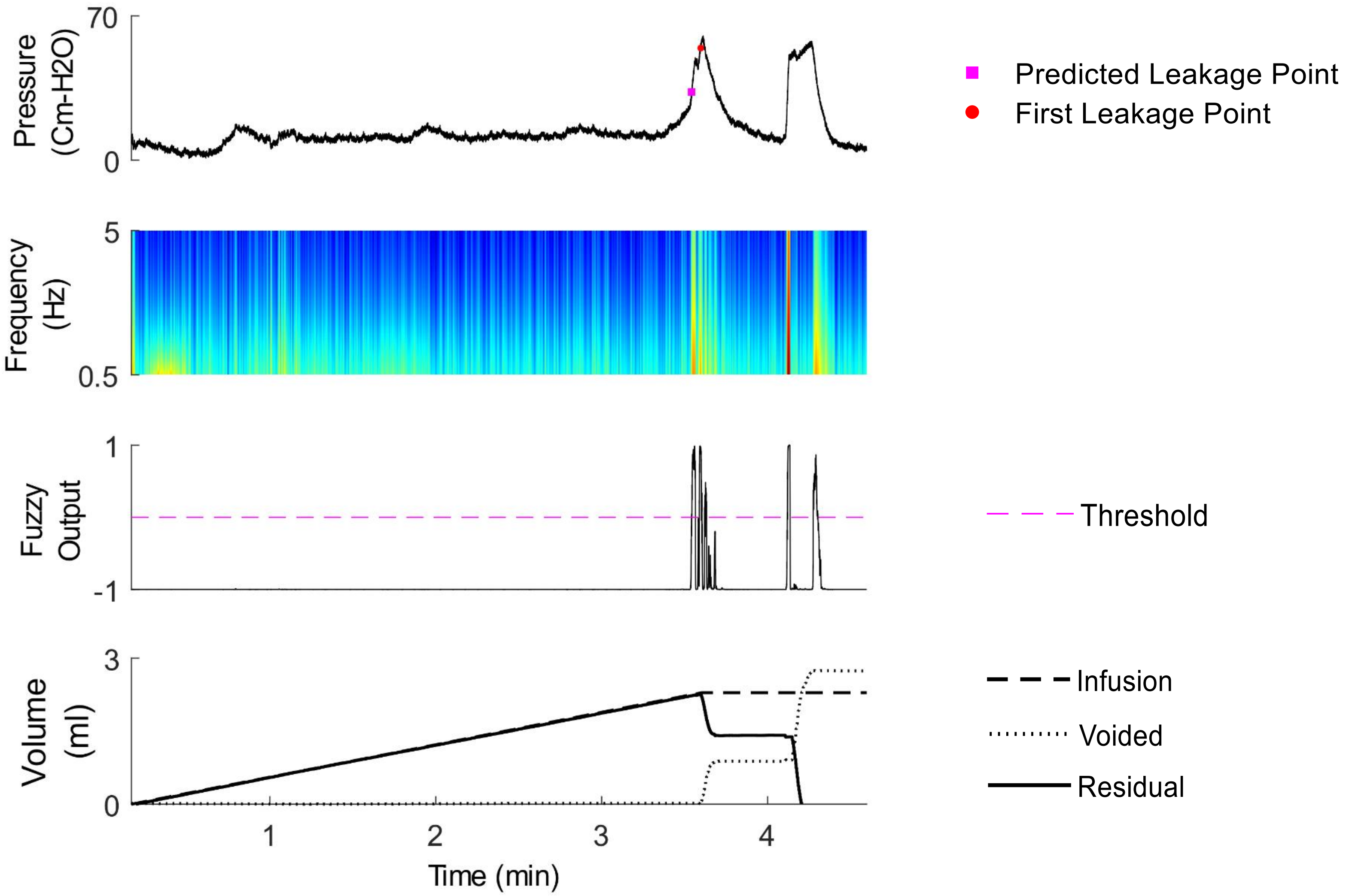

Gender = *male*, **Weight =** *2.8 kg*, **Infusion Rate =** *120 ml/h*, **Prediction Time =** *4.18 s*, **Delay Time =** *3.86 s*, **Pressure Increase =** *13.4 cmH2O*

Cat5_Trial4_Normal

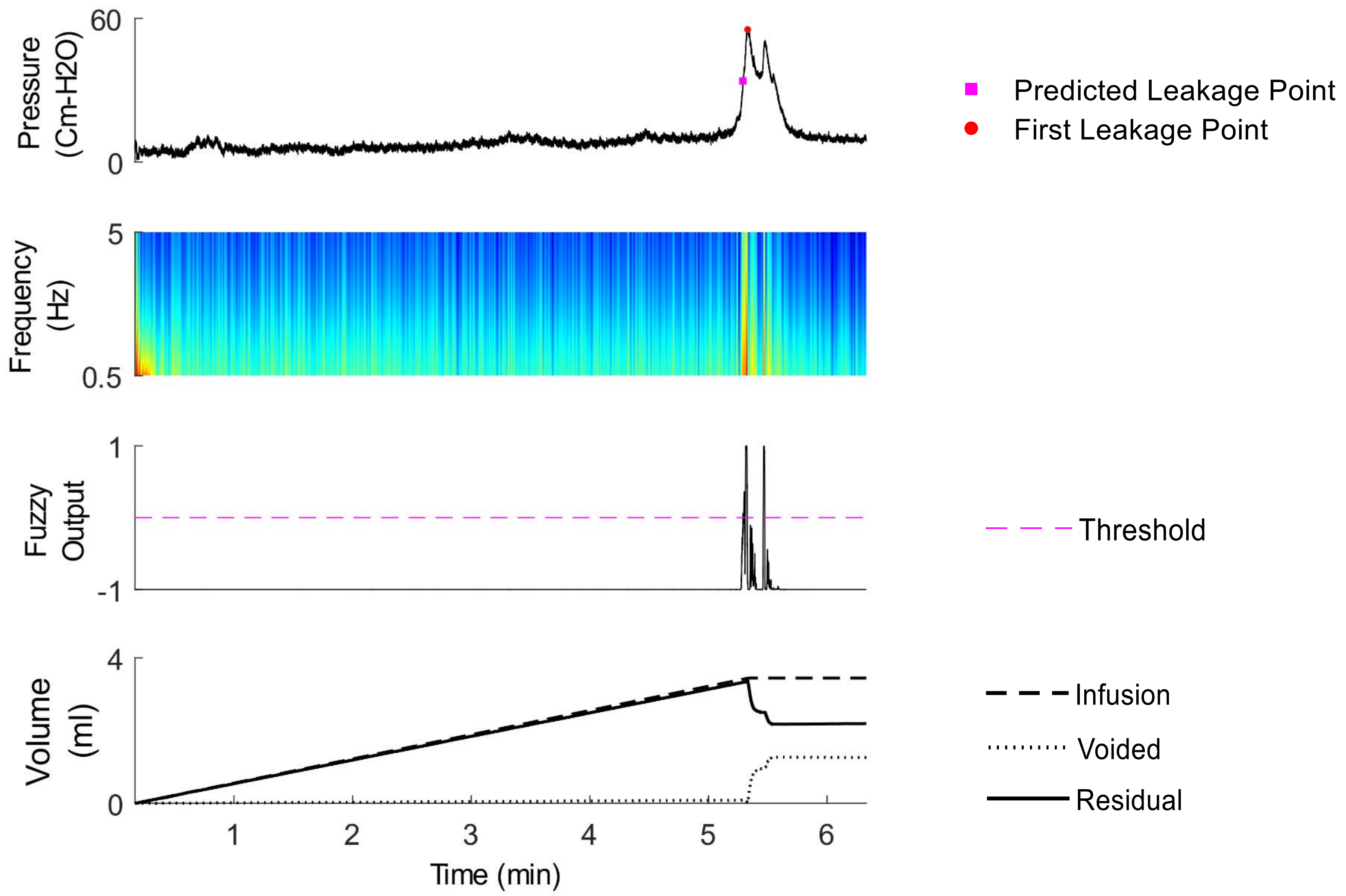

Gender = *male*, **Weight =** *2.8 kg*, **Infusion Rate =** *120 ml/h*, **Prediction Time =** *3.58 s*, **Delay Time =** *3.92 s*, **Pressure Increase =** *12.92 cmH2O*

Cat5_Trial5_Normal

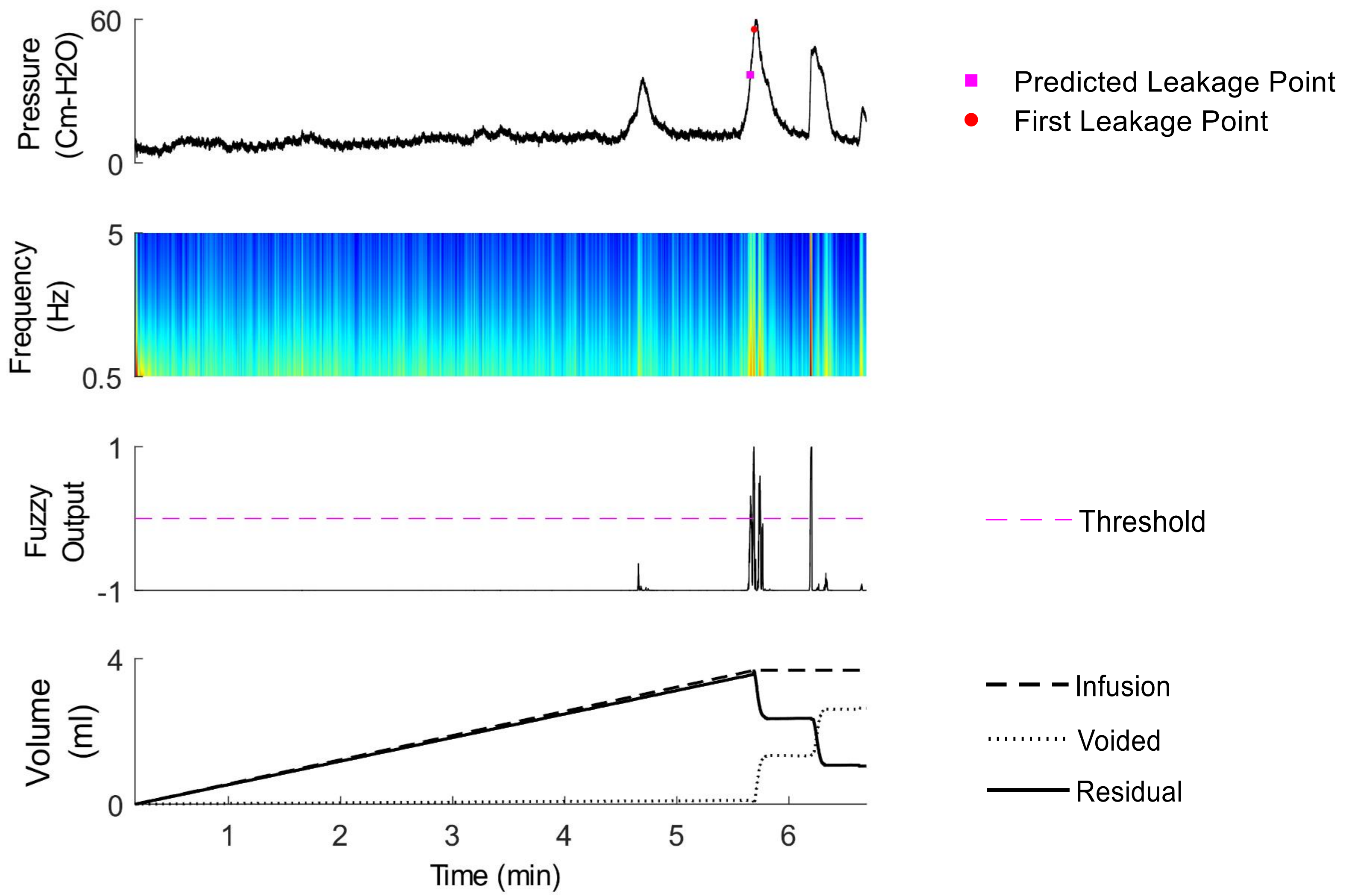

Gender = *male*, **Weight** *= 2.8 kg*, **Infusion Rate =** *120 ml/h*, **Prediction Time =** *1.96 s*, **Delay Time =** *4.24 s*, **Pressure Increase =** *22.44 cmH2O*

Cat5_Trial6_Normal

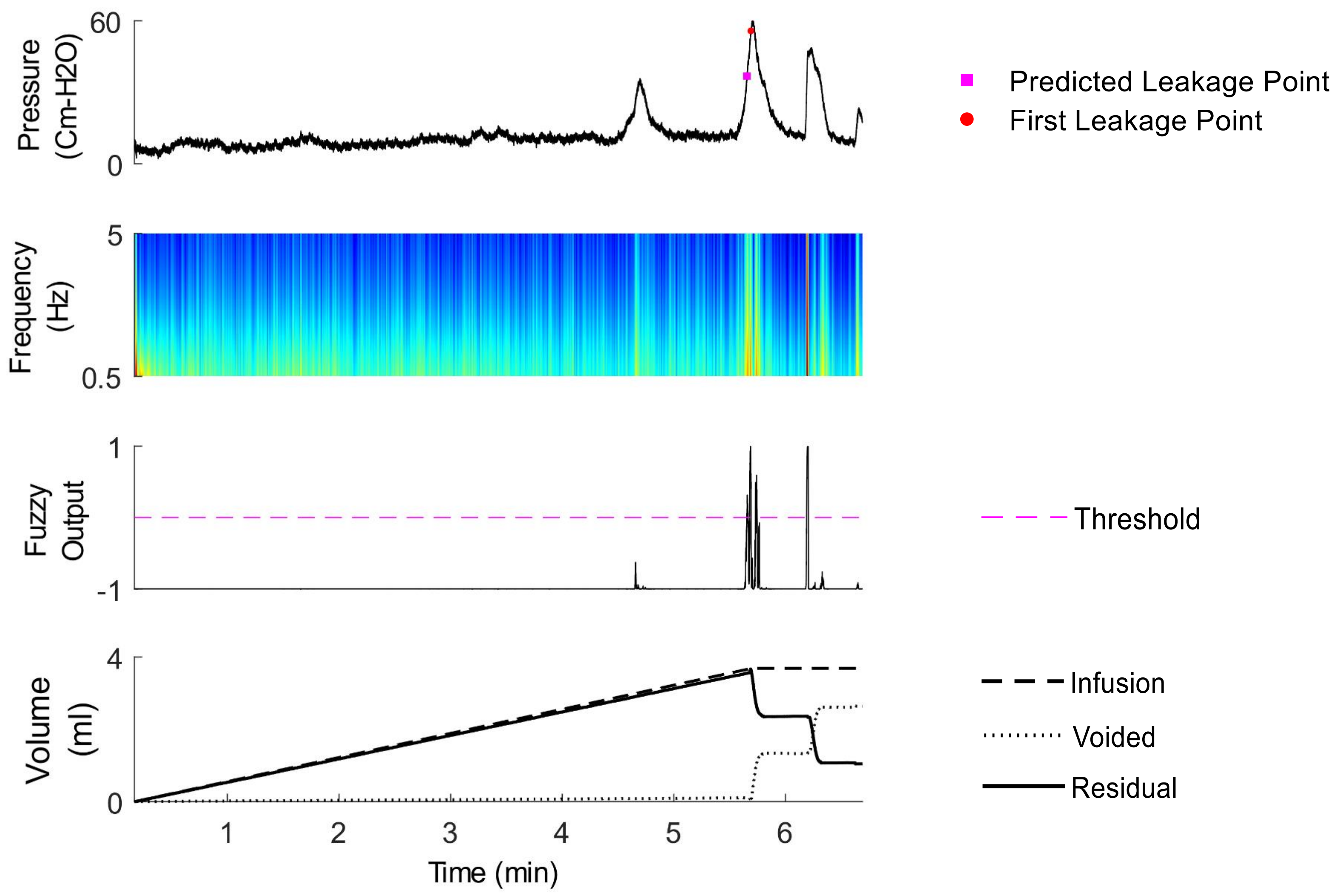

Gender = *male*, **Weight =** *2.8 kg*, **Infusion Rate =** *120 ml/h*, **Prediction Time =** *2 s*, **Delay Time =** *5.08 s*, **Pressure Increase =** *24.43 cmH2O*

Cat5_Trial7_Normal

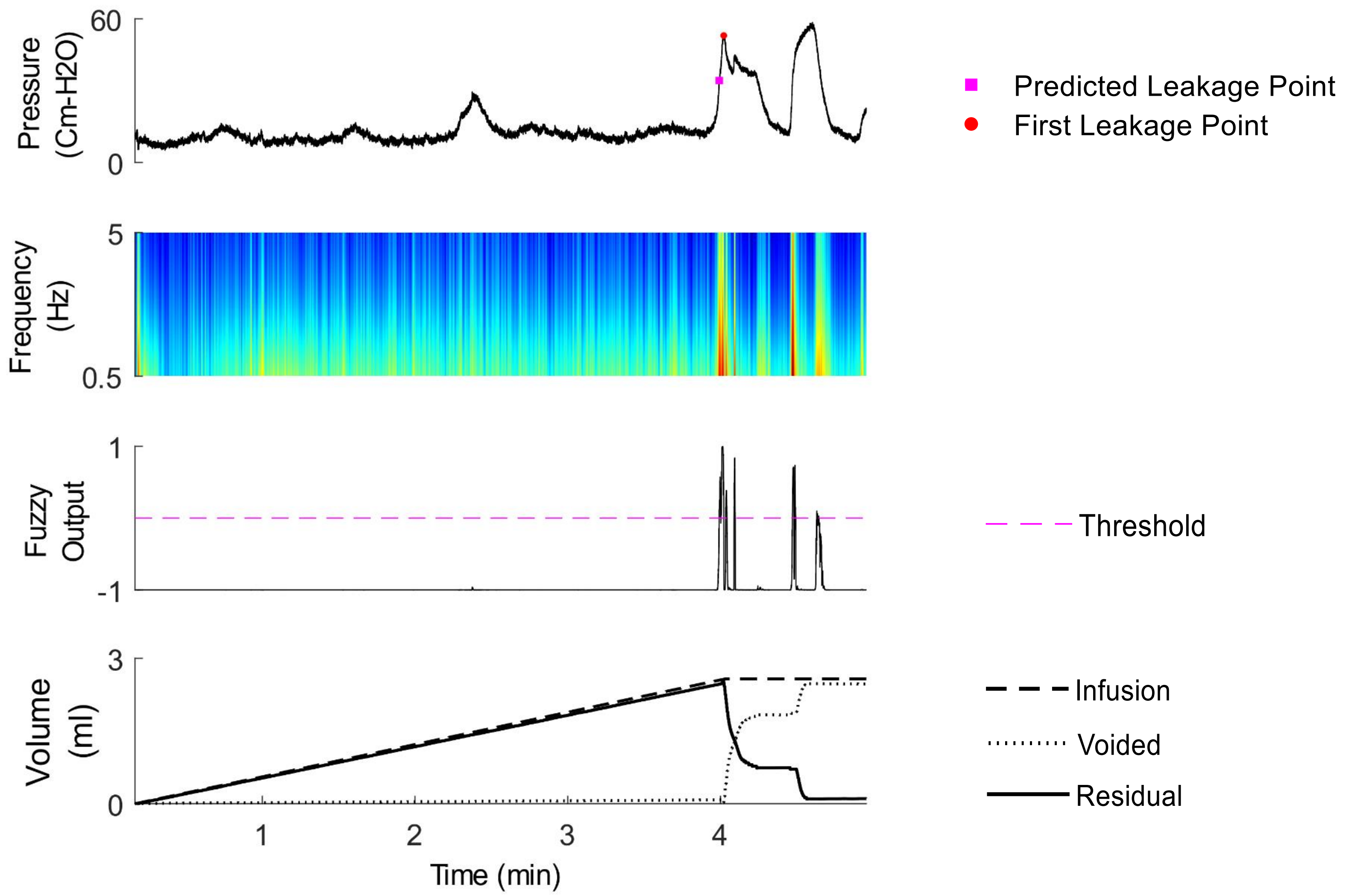

Gender = *male*, **Weight =** *2.8 kg*, **Infusion Rate =** *120 ml/h*, **Prediction Time =** *1.76 s*, **Delay Time =** *2.36 s*, **Pressure Increase =** *20.29 cmH2O*

Cat6_Trial1_Normal

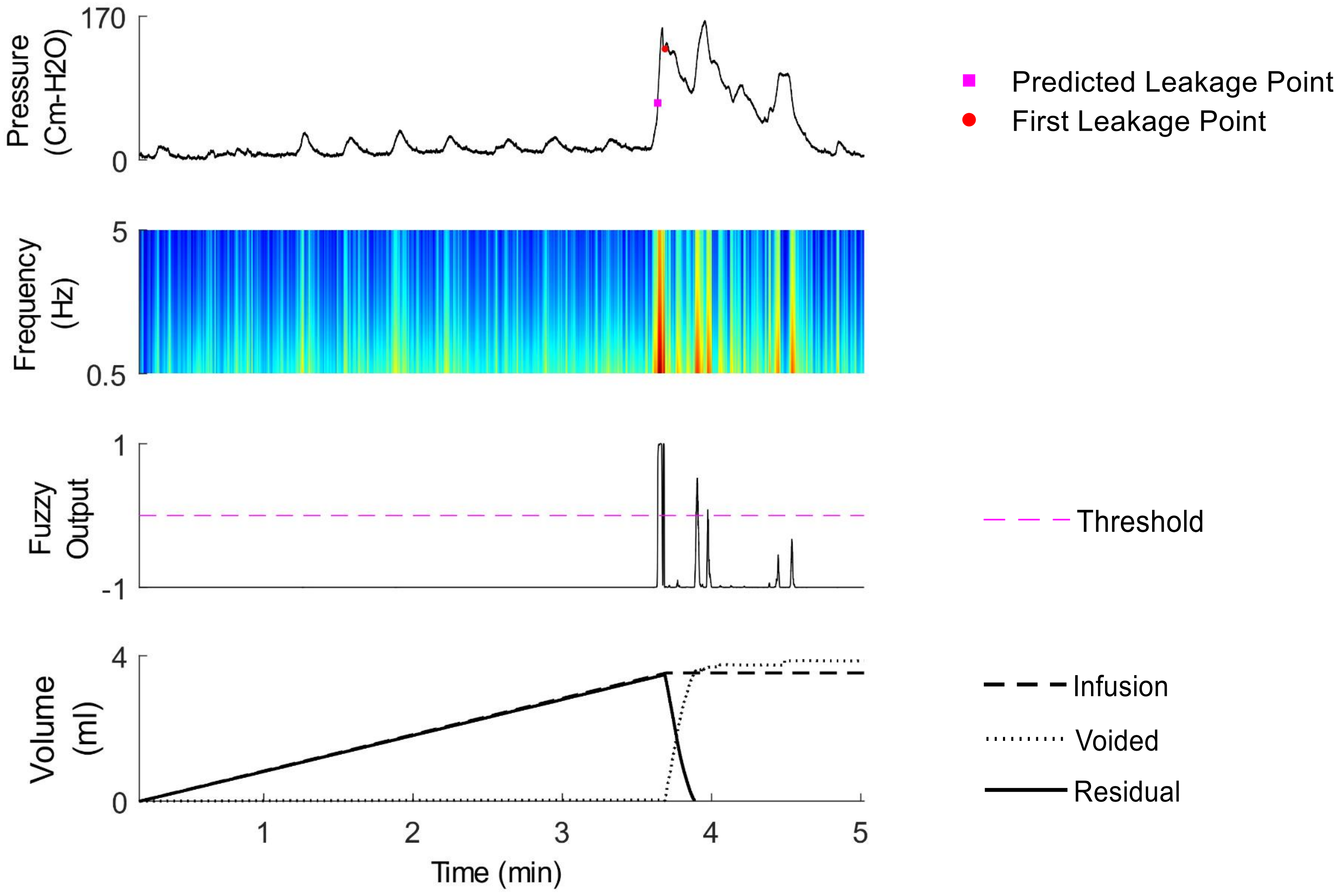

Gender = *male*, **Weight =** *2.35 kg*, **Infusion Rate =** *60 ml/h*, **Prediction Time =** *2.96 s*, **Delay Time =** *2.48 s*, **Pressure Increase =** *53.66 cmH2O*

Cat6_Trial2_Normal

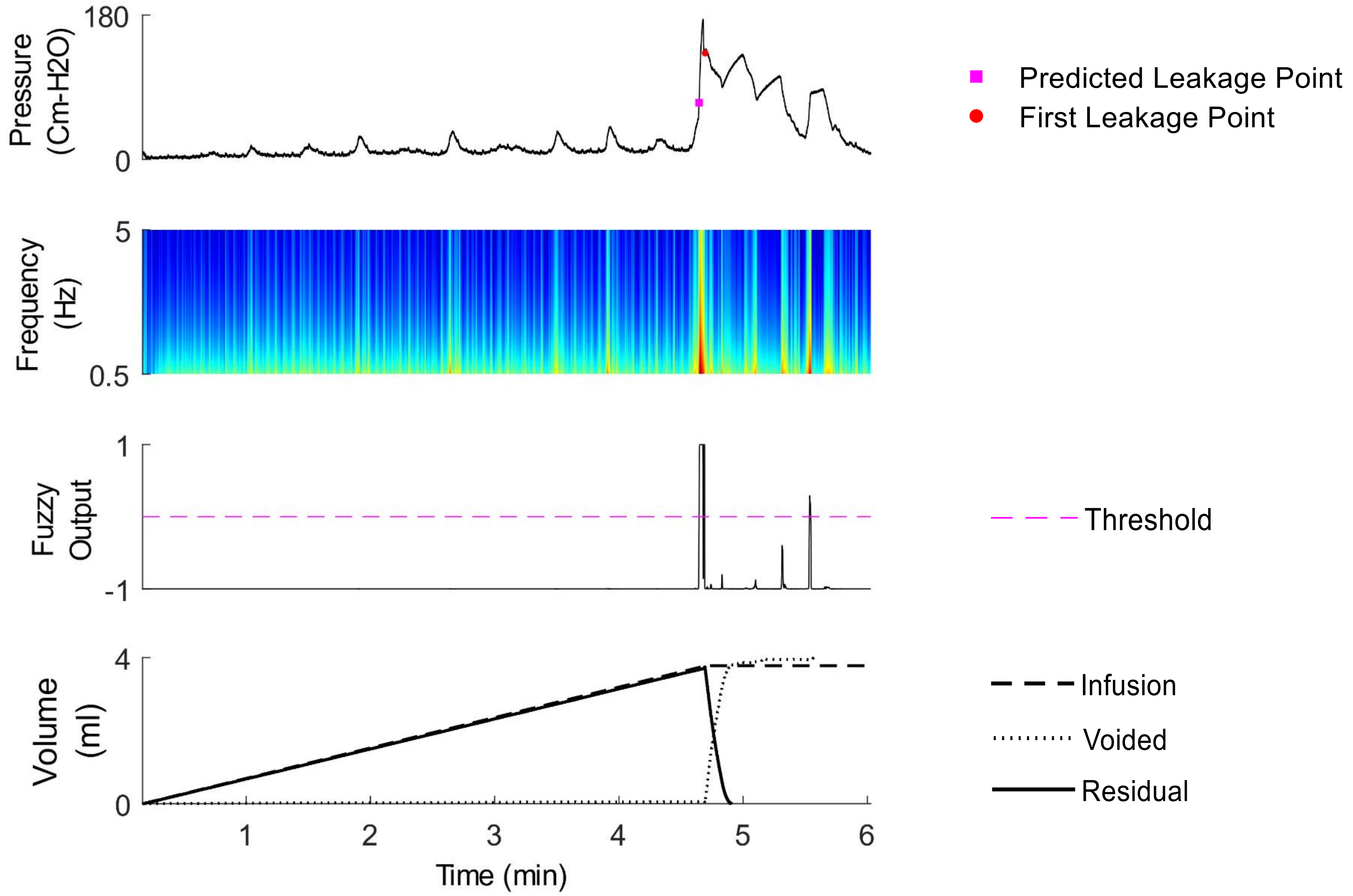

Gender = *male*, **Weight =** *2.35 kg*, **Infusion Rate =** *50 ml/h*, **Prediction Time =** *2.9 s*, **Delay Time =** *3.8 s*, **Pressure Increase =** *57.61 cmH2O*

Cat6_Trial3_Normal

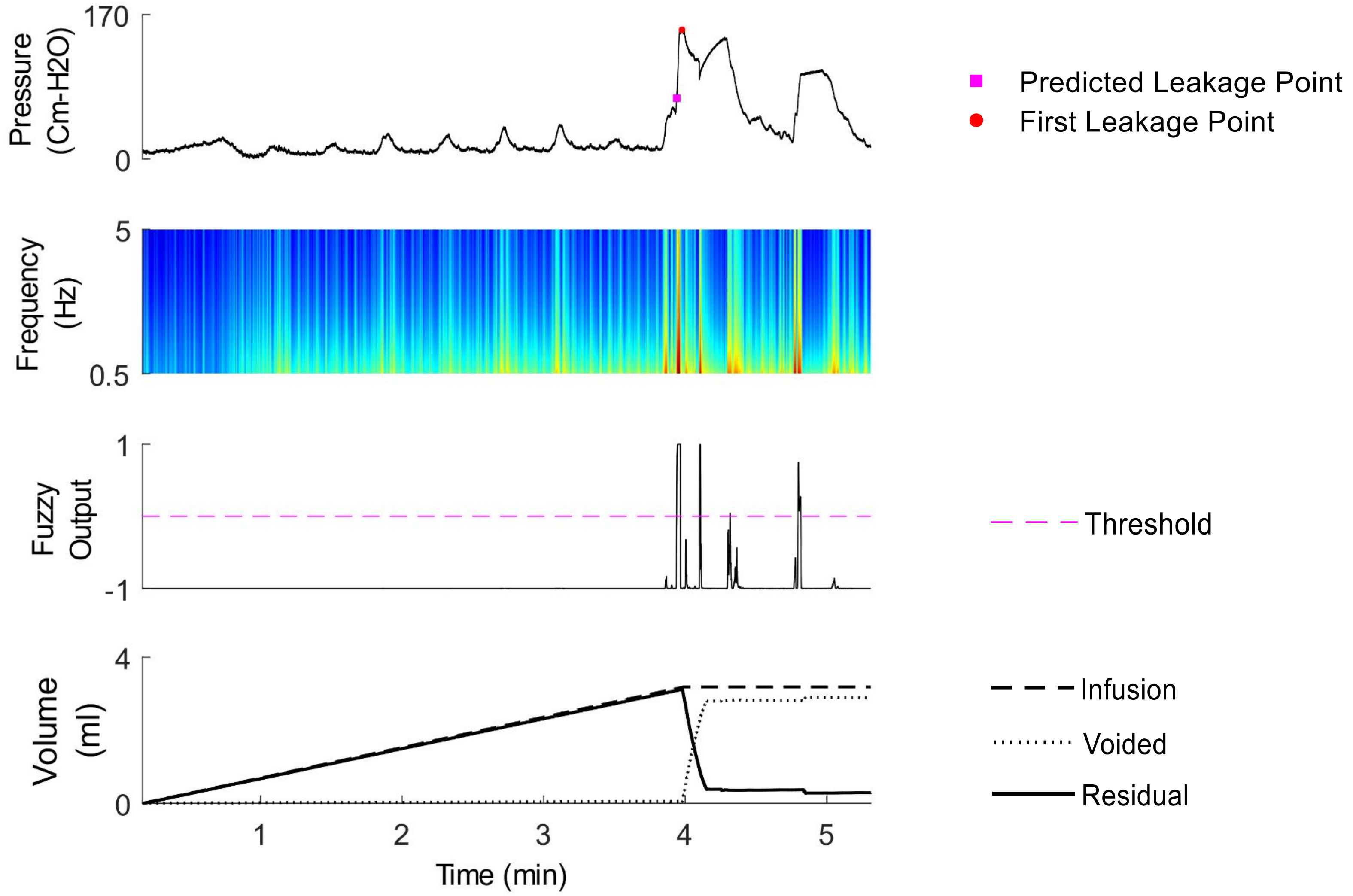

Gender = *male*, **Weight =** *2.35 kg*, **Infusion Fluid = ,** **Infusion Rate =** *50 ml/h*, **Prediction Time =** *2.24 s*, **Delay Time =** *5.82 s*, **Pressure Increase =** *52.65 cmH2O*

Cat6_Trial4_Normal

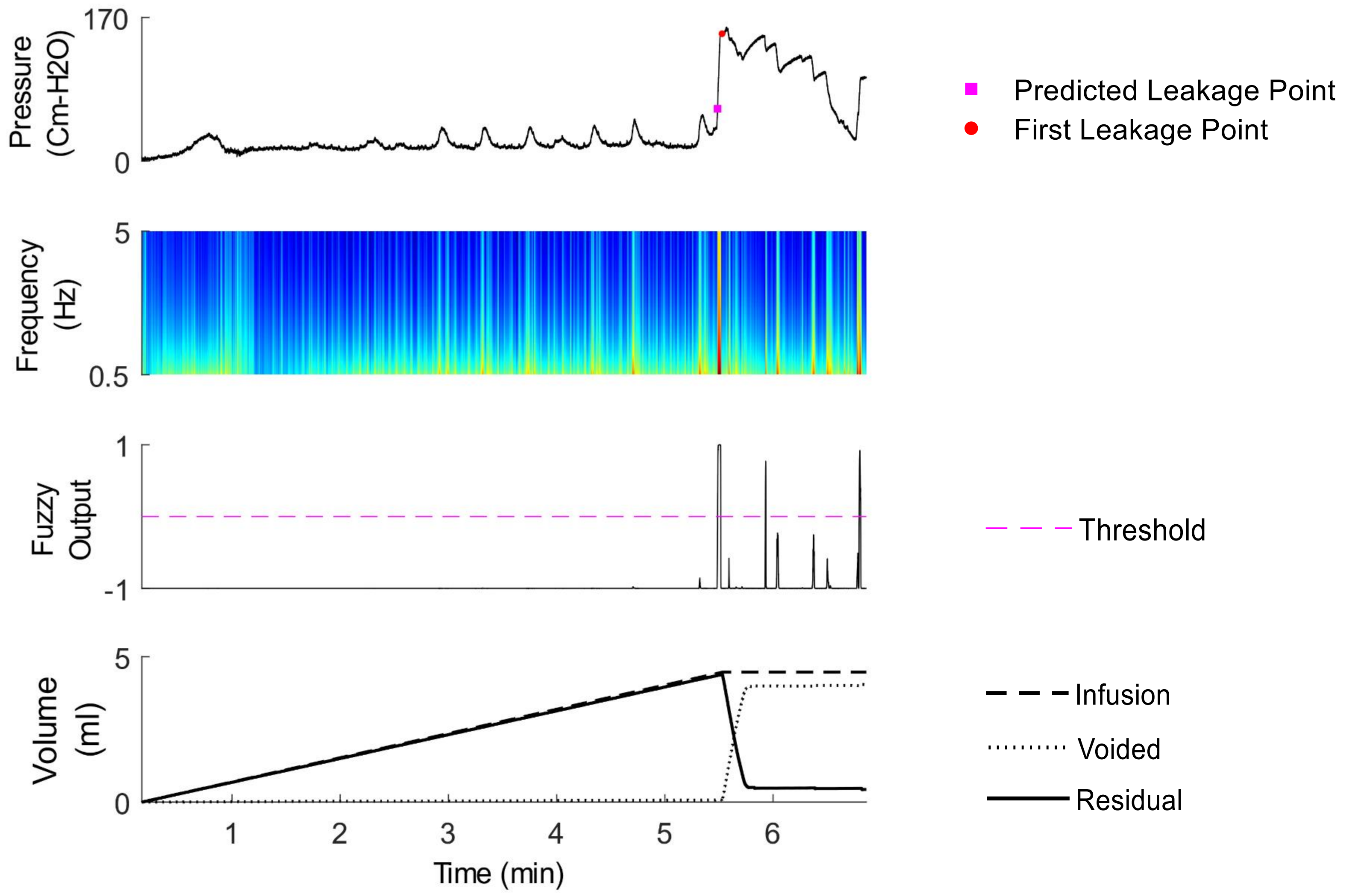

Gender = *male*, **Weight =** *2.35 kg*, **Infusion Rate =** *50 ml/h*, **Prediction Time =** *2.38 s*, **Delay Time =** *2.82 s*, **Pressure Increase =** *28.81 cmH2O*

Cat6_Trial5_Normal

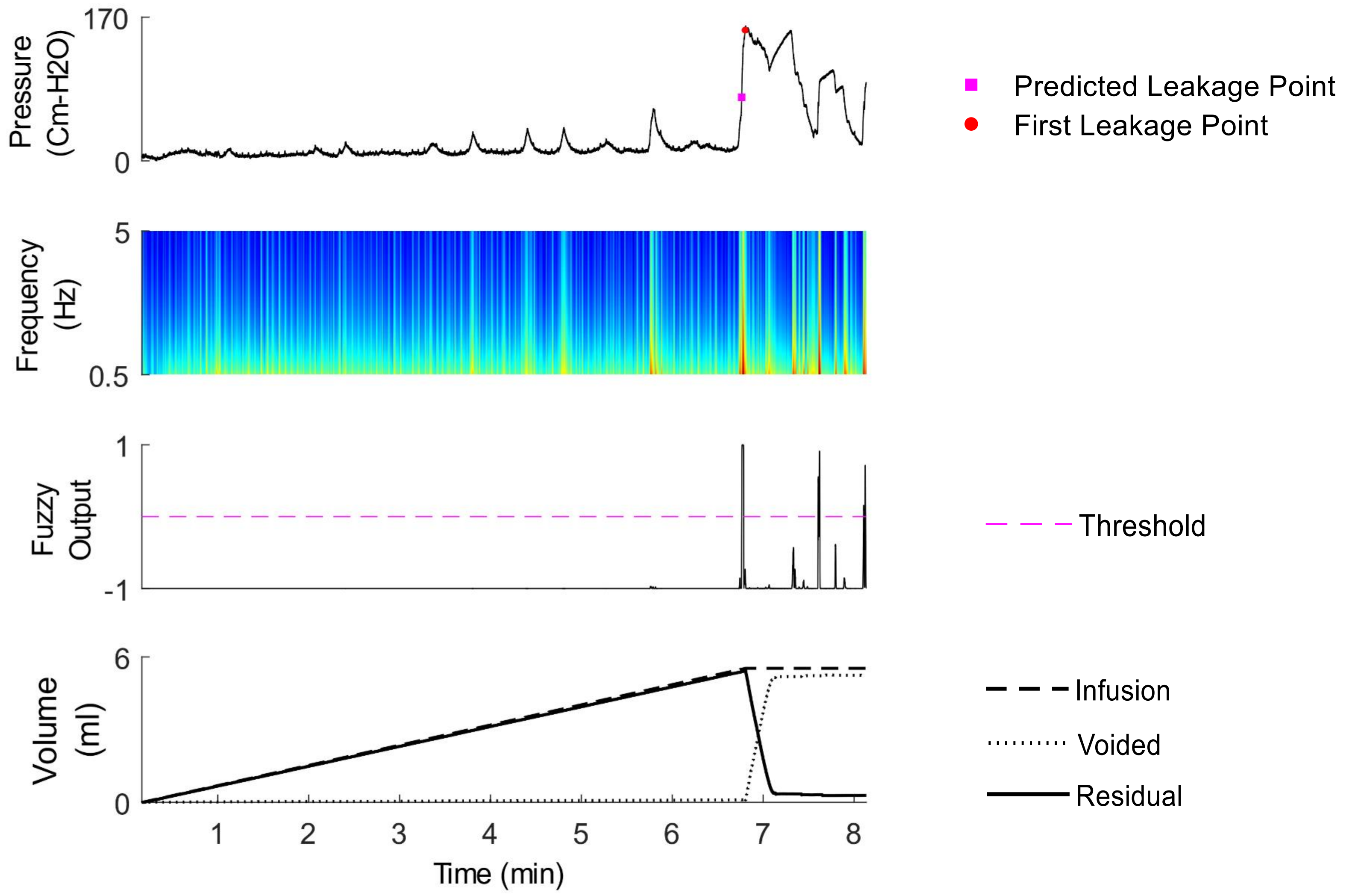

Gender = *male*, **Weight =** *2.35 kg*, **Infusion Rate =** *50 ml/h*, **Prediction Time =** *2.22 s*, **Delay Time =** *2.5 s*, **Pressure Increase =** *57.66 cmH2O*

Cat6_Trial6_Normal

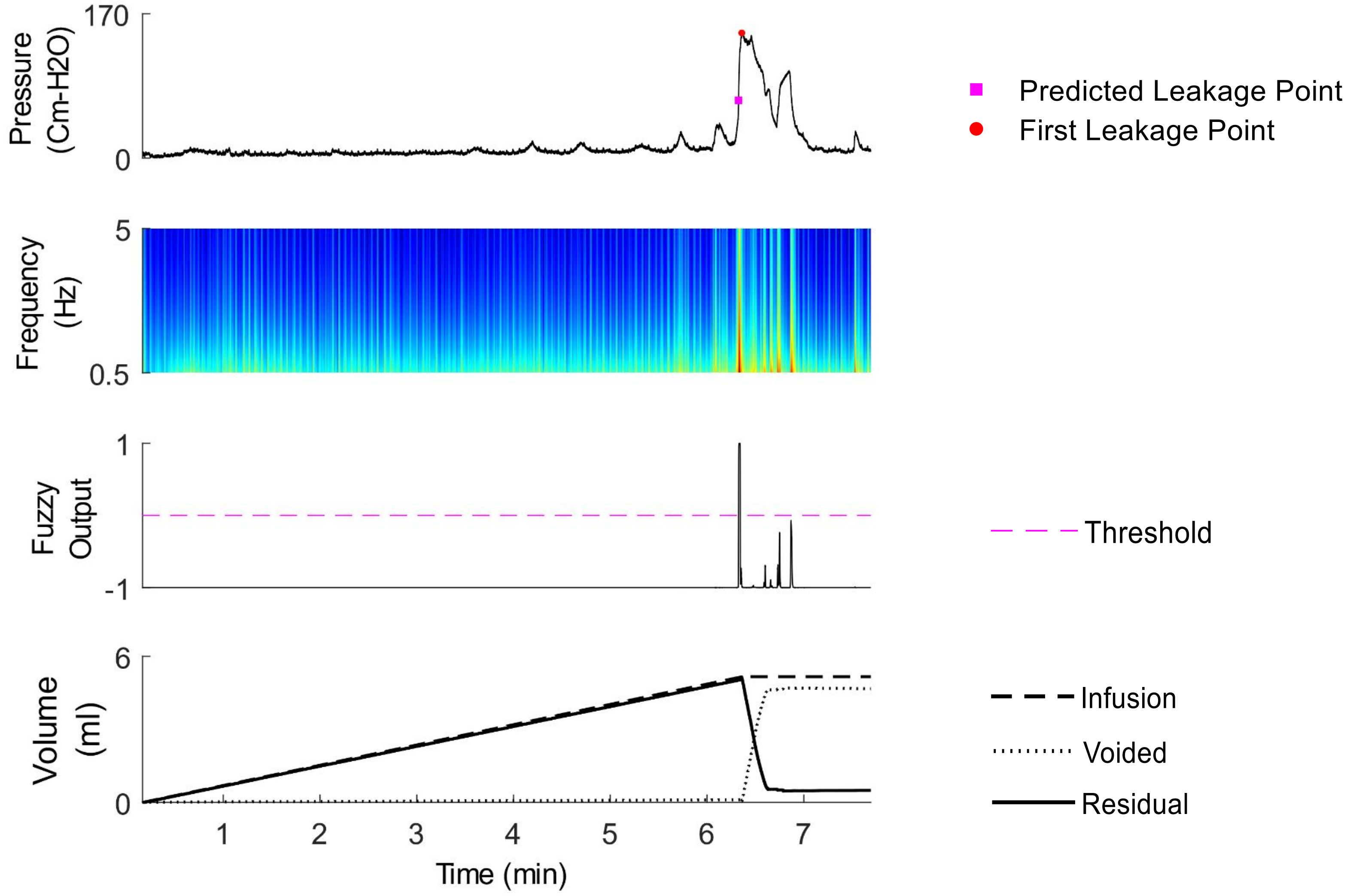

Gender = *male*, **Weight =** *2.35 kg*, **Infusion Rate =** *50 ml/h*, **Prediction Time =** *1.98 s*, **Delay Time =** *2.5 s*, **Pressure Increase =** *48.13 cmH2O*

Cat7_Trial1_Normal

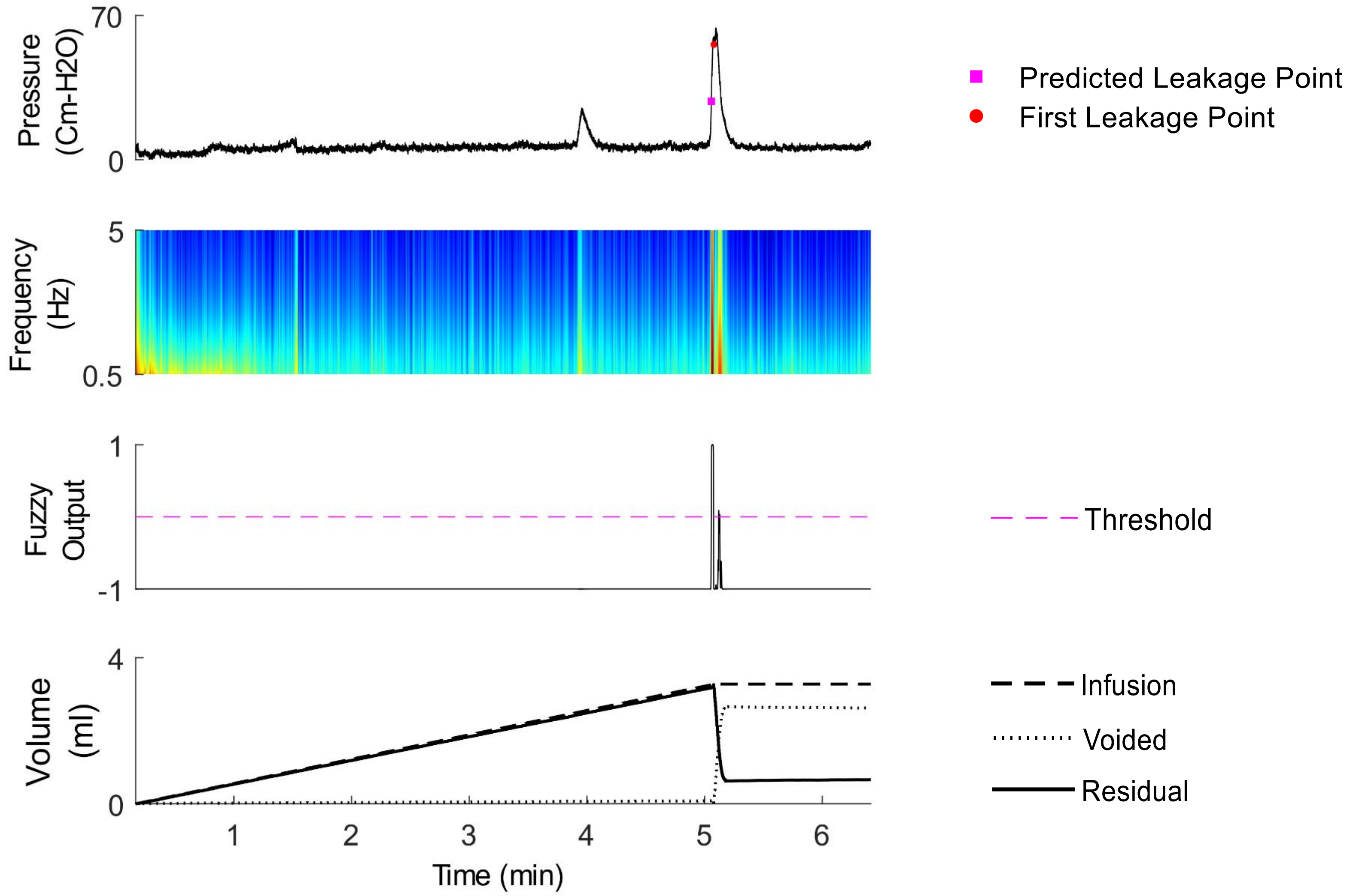

Gender = *male*, **Weight =** *2.93 kg*, **Infusion Rate =** *40 ml/h*, **Prediction Time =** *1.28 s*, **Delay Time =** *0.72 s*, **Pressure Increase =** *18.2 cmH2O*

Cat7_Trial2_Normal

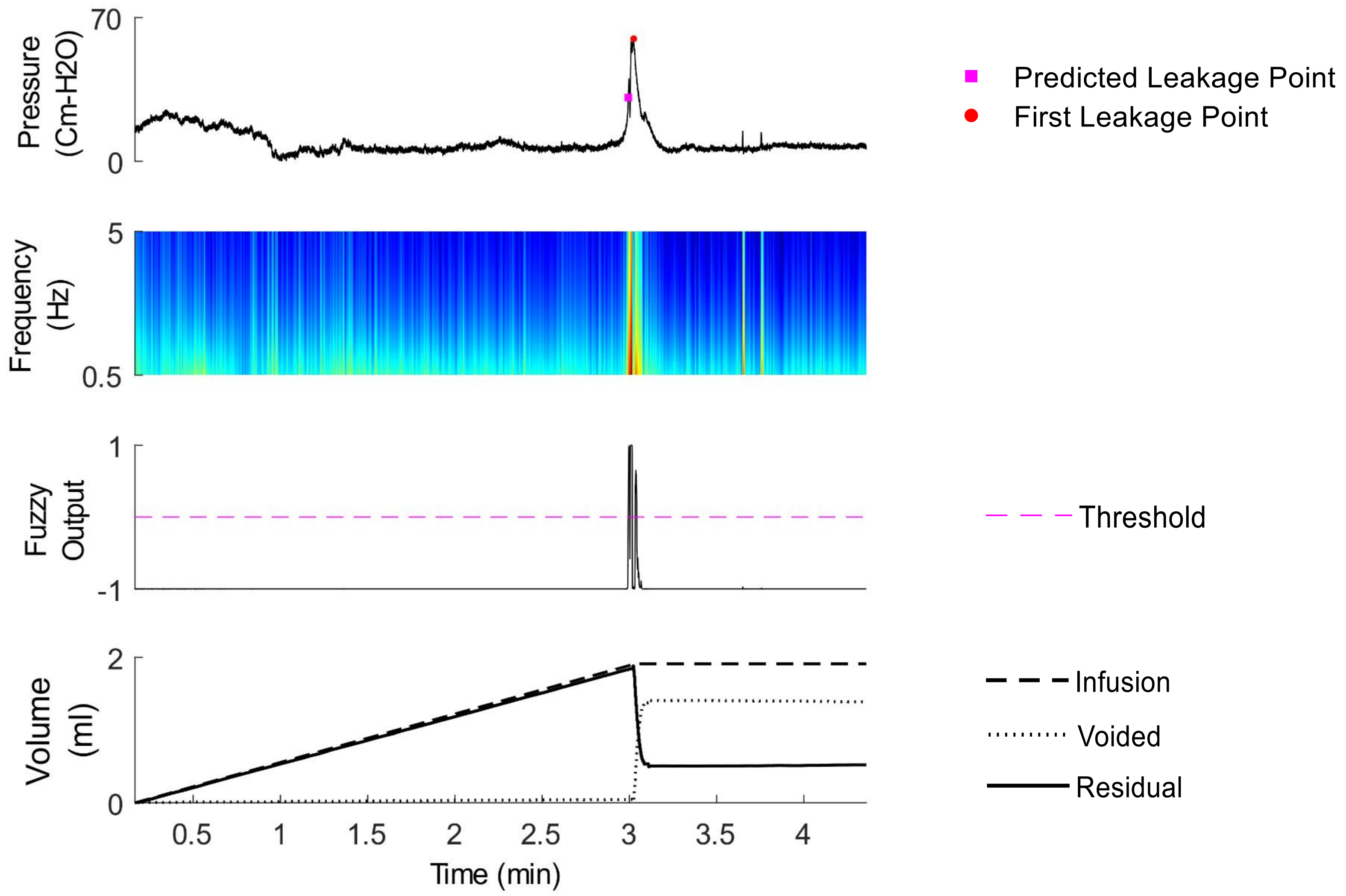

Gender = *male*, **Weight =** *2.93 kg*, **Infusion Rate =** *40 ml/h*, **Prediction Time =** *1.86 s*, **Delay Time =** *1.92 s*, **Pressure Increase =** *19.79 cmH2O*

Cat7_Trial3_Normal

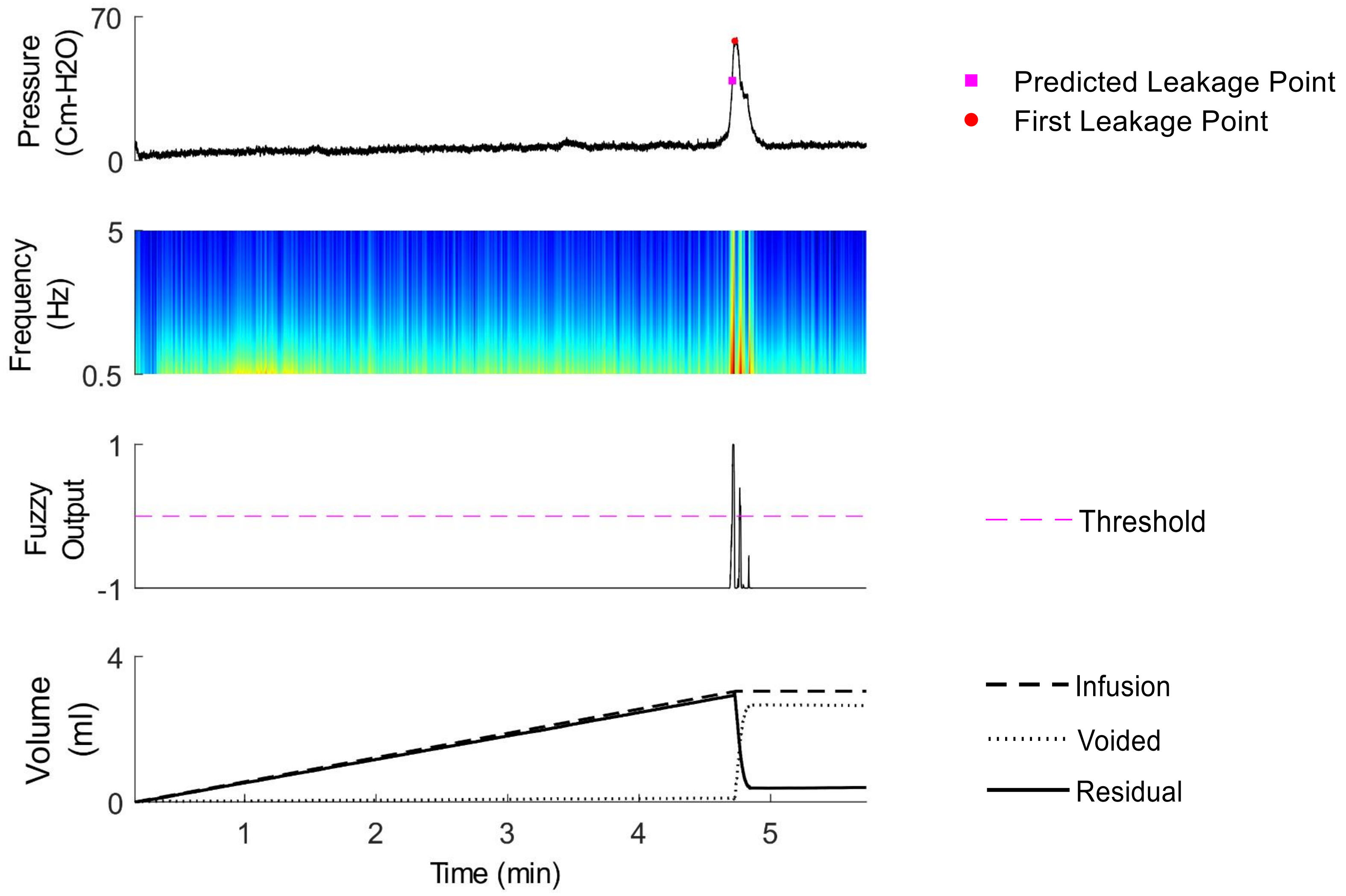

Gender = *male*, **Weight =** *2.93 kg*, **Infusion Rate =** *40 ml/h*, **Prediction Time =** *1.12 s*, **Delay Time =** *2.22 s*, **Pressure Increase =** *25.25 cmH2O*

Figure S37

Cat7_Trial4_Normal

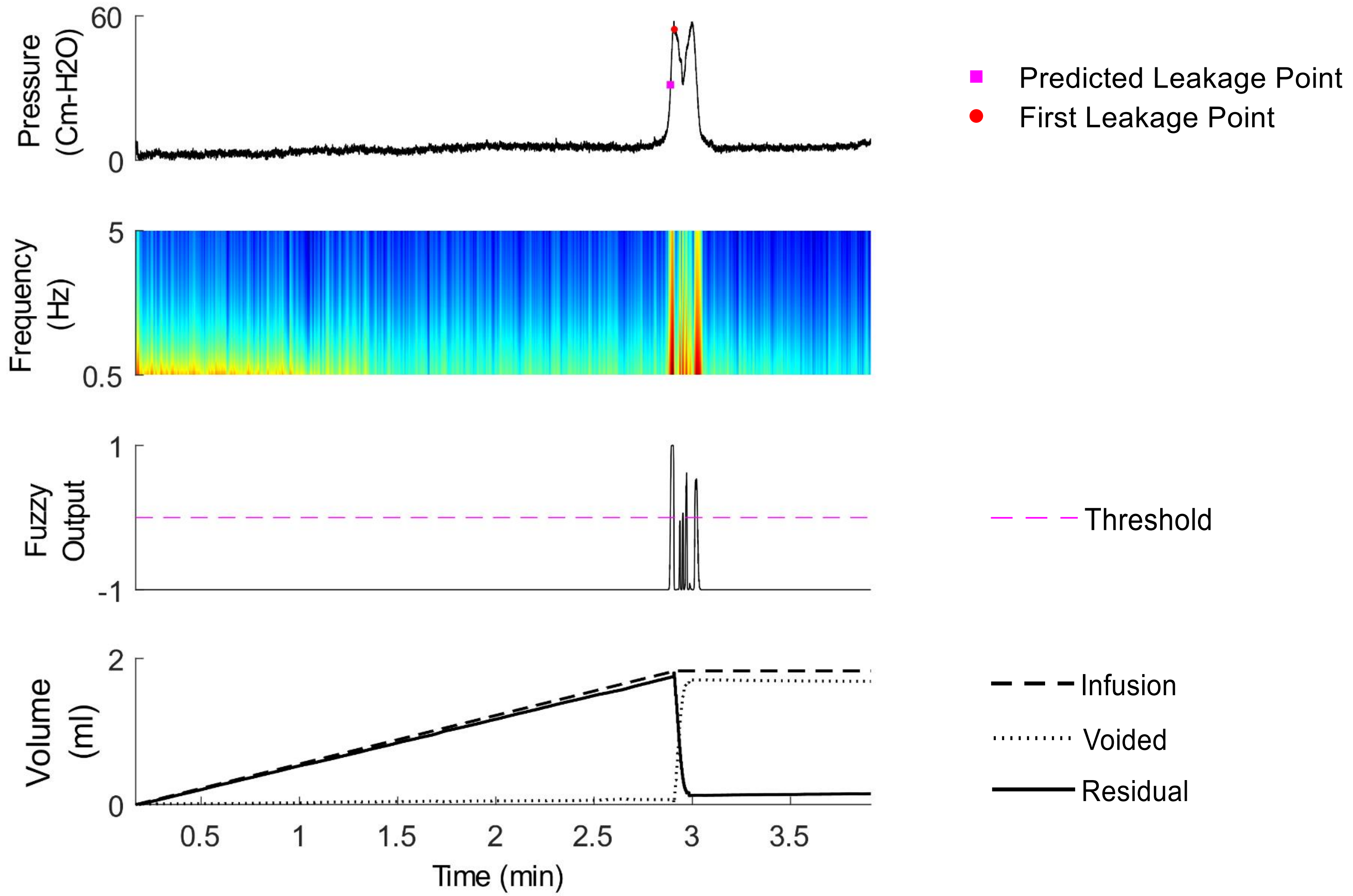

Gender = *male*, **Weight =** *2.93 kg*, **Infusion Rate =** *40 ml/h*, **Prediction Time =** *1.2 s*, **Delay Time =** *1.06 s*, **Pressure Increase =** *18.57 cmH2O*

Cat7_Trial5_Normal

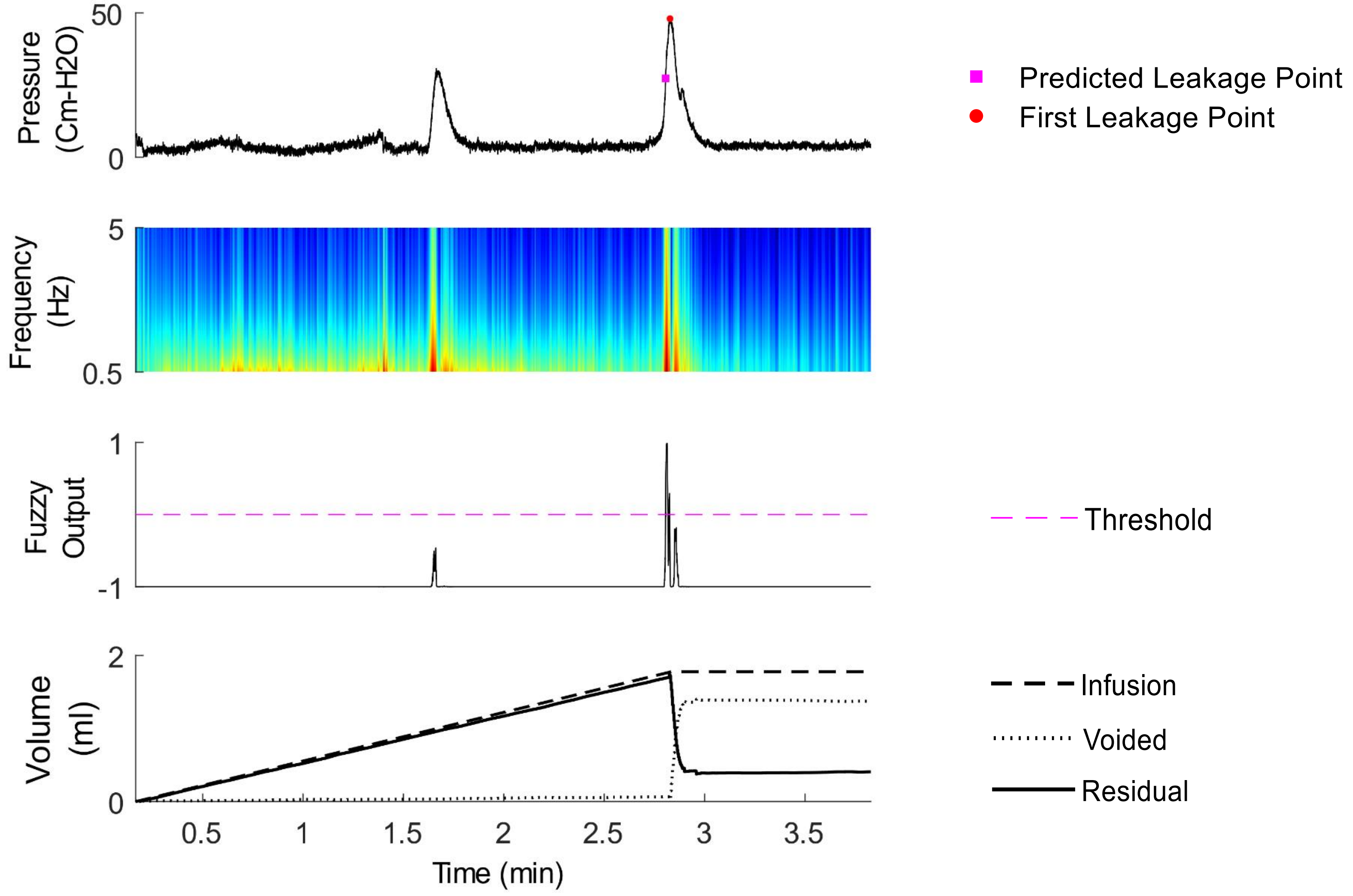

Gender = *male*, **Weight =** *2.93 kg*, **Infusion Rate =** *40 ml/h*, **Prediction Time =** *1.28 s*, **Delay Time =** *1.1 s*, **Pressure Increase =** *19.68 cmH2O*

Cat7_Trial6_Normal

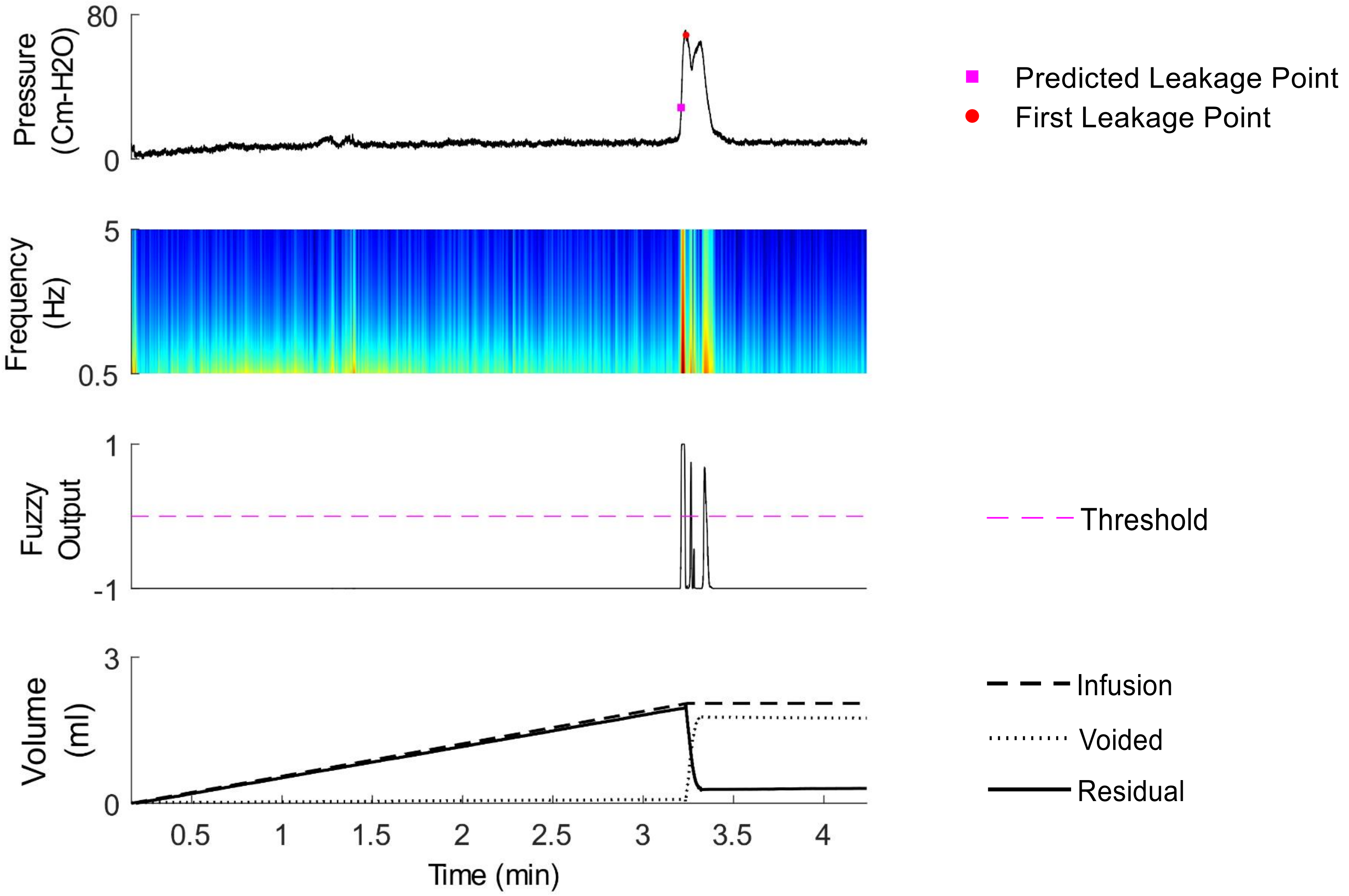

Gender = *male*, **Weight =** *2.93 kg*, **Infusion Rate =** *40 ml/h*, **Prediction Time =** *1.62 s*, **Delay Time =** *0.5 s*, **Pressure Increase =** *14.36 cmH2O*

Cat7_Trial7_Normal

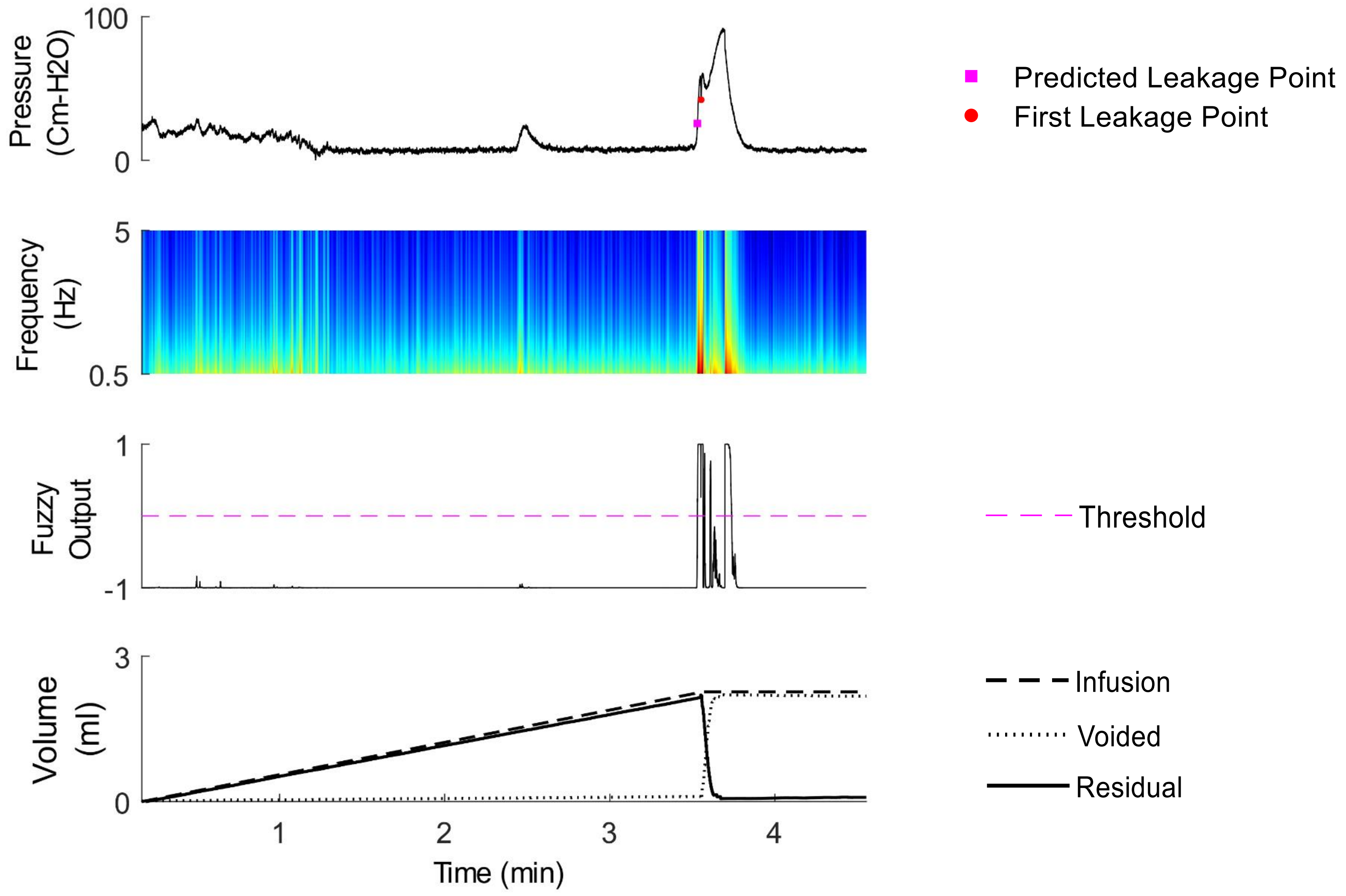

Gender = *male*, **Weight =** *2.93 kg*, **Infusion Rate =** *40 ml/h*, **Prediction Time =** *1.36 s*, **Delay Time =** *0.8 s*, **Pressure Increase =** *14.61 cmH2O*

Cat7_Trial8_Normal

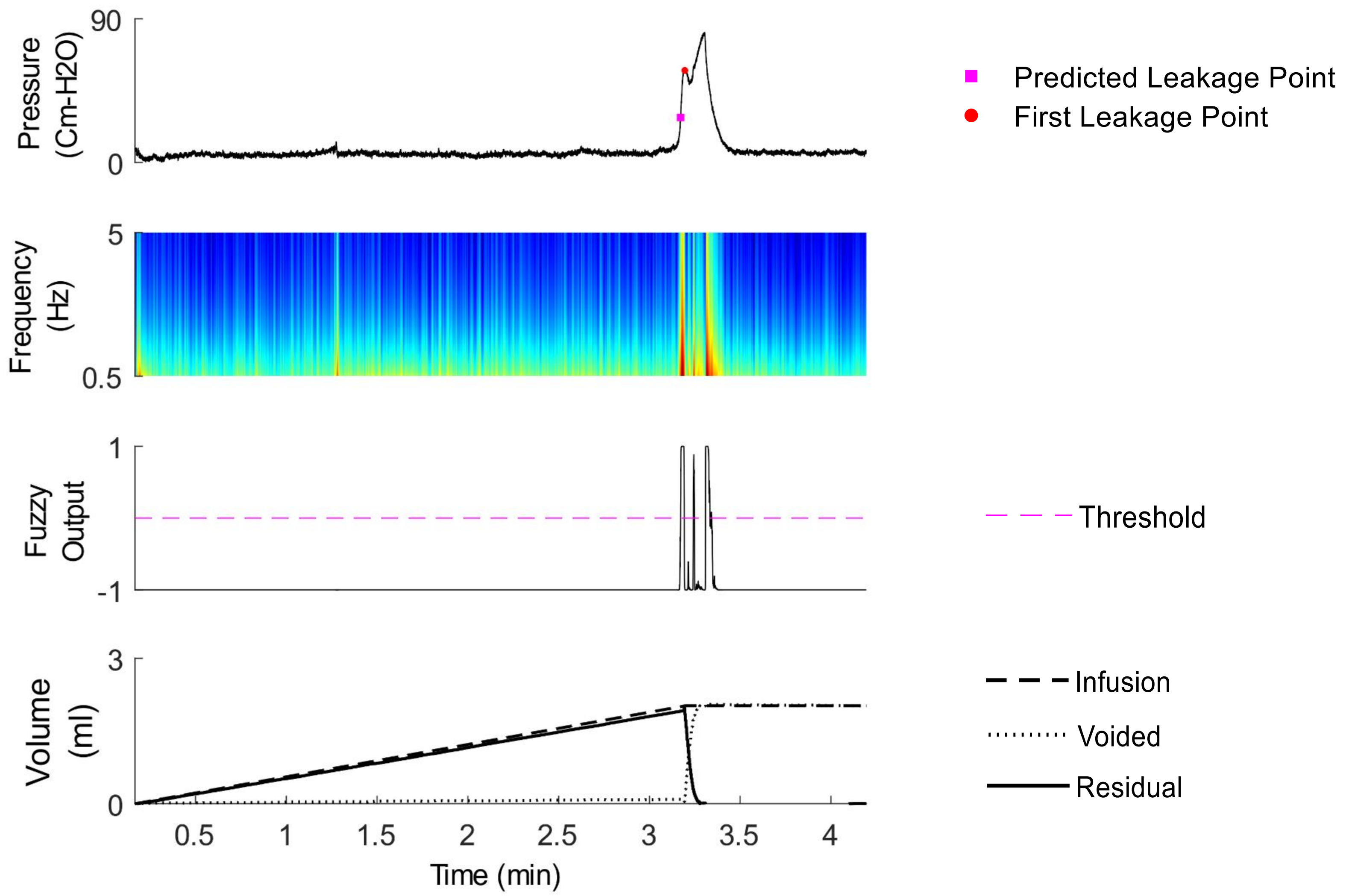

Gender = *male*, **Weight =** *2.93 kg*, **Infusion Rate =** *40 ml/h*, **Prediction Time =** *1.34 s*, **Delay Time =** *0.64 s*, **Pressure Increase =** *14.47 cmH2O*

Cat7_Trial9_Normal

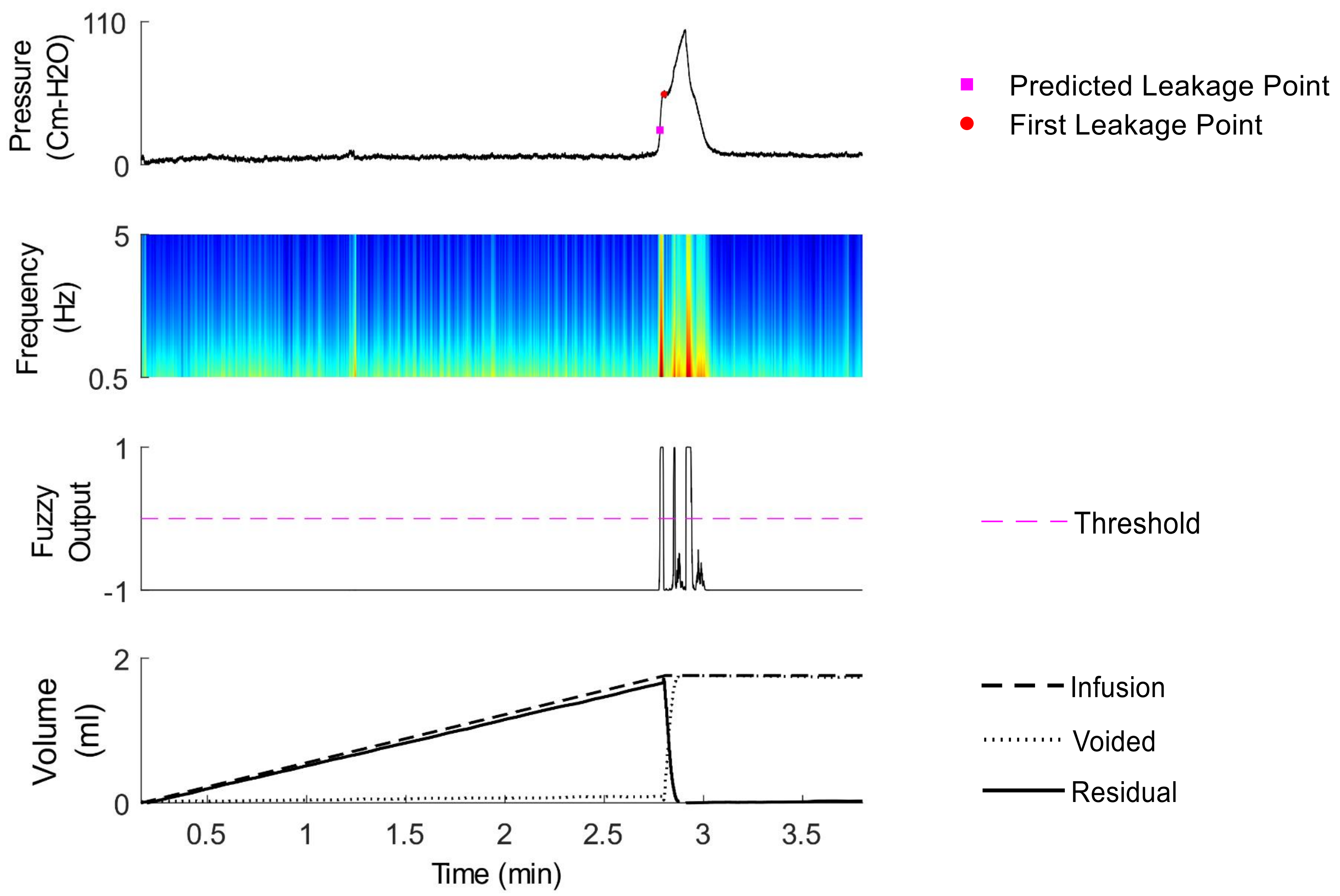

Gender = *male*, **Weight =** *2.93 kg*, **Infusion Rate =** *40 ml/h*, **Prediction Time =** *1.22 s*, **Delay Time =** *0.46 s*, **Pressure Increase =** *14.09 cmH2O*

Cat7_Trial10_Normal

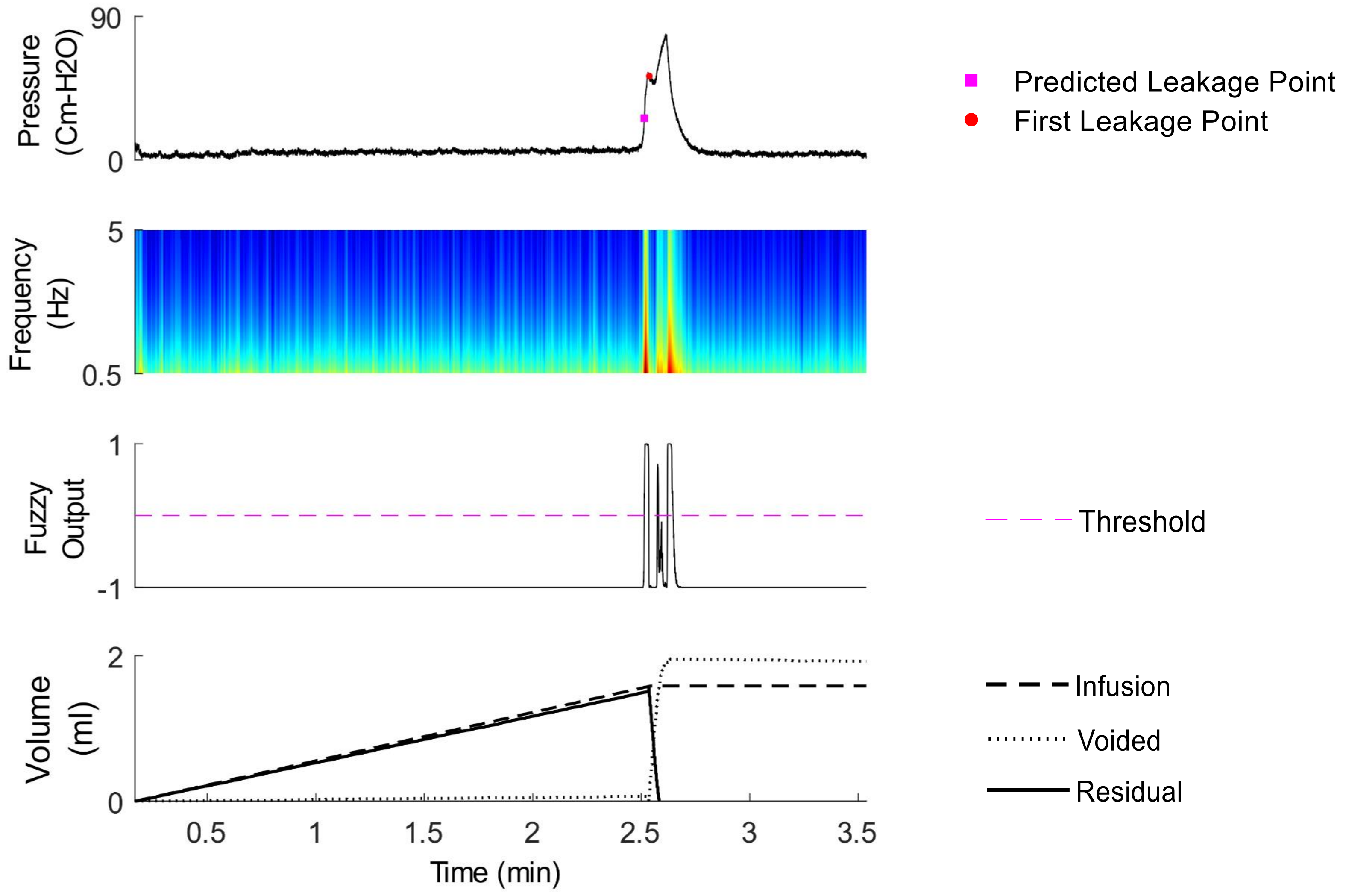

Gender = *male*, **Weight =** *2.93 kg*, **Infusion Rate =** *40 ml/h*, **Prediction Time =** *1.38 s*, **Delay Time =** *0.52 s*, **Pressure Increase =** *14.01 cmH2O*

Cat8_Trial1_Normal

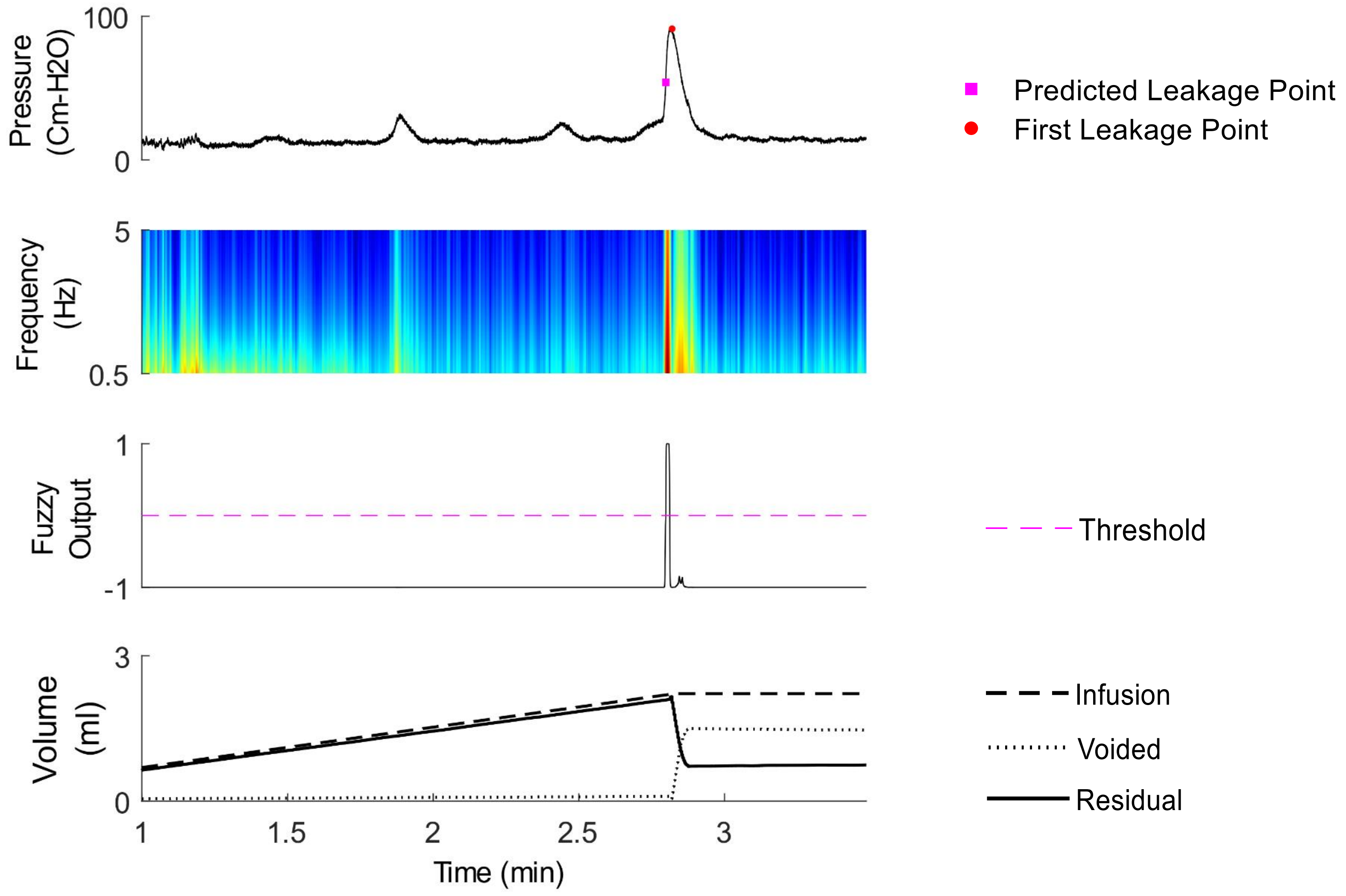

Gender = *male*, **Weight =** *1.90 kg*, **Infusion Rate =** *50 ml/h*, **Prediction Time =** *1.34 s*, **Delay Time =** *1.1 s*, **Pressure Increase =** *24 cmH2O*

Cat8_Trial2_Normal

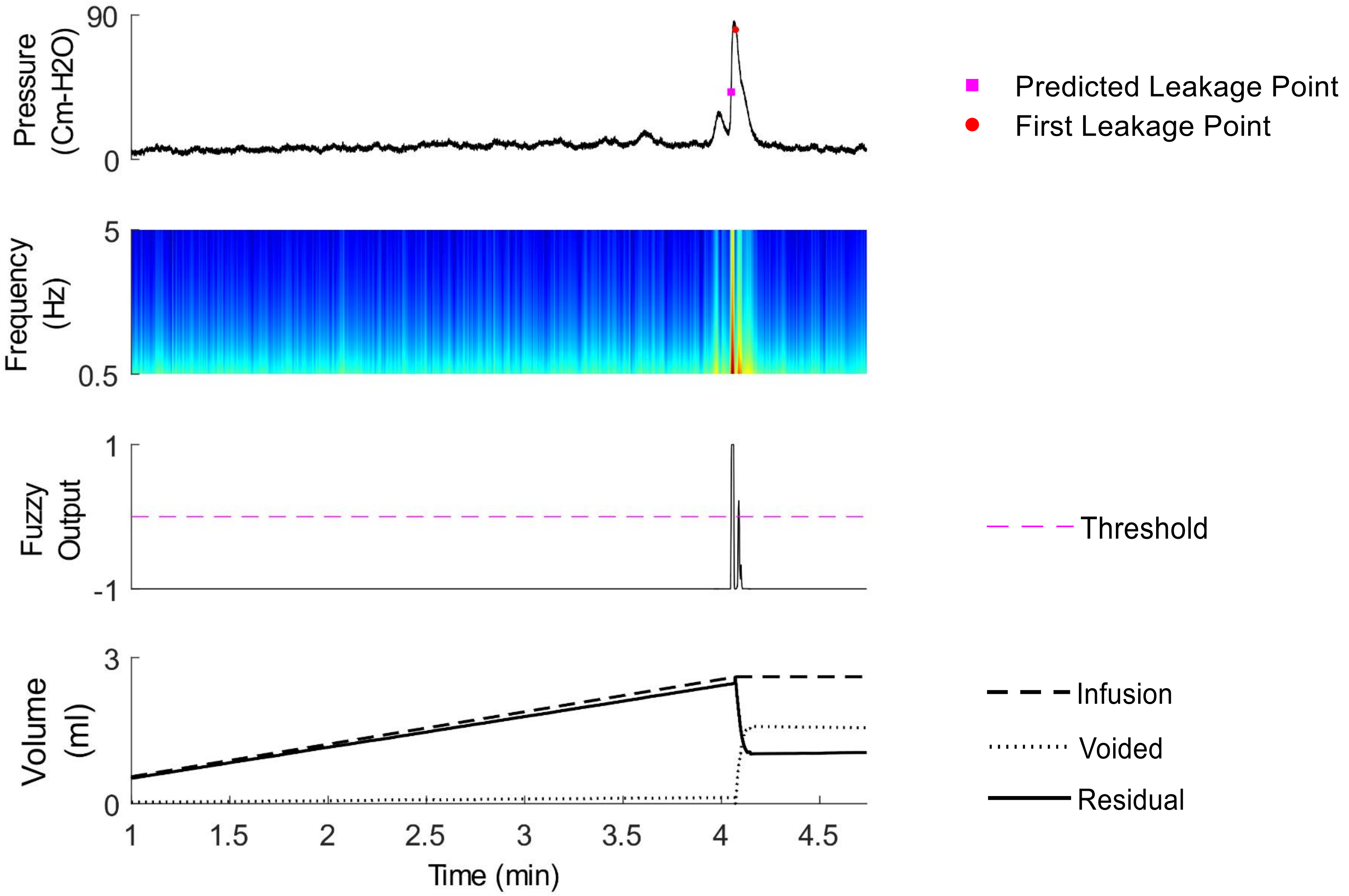

Gender = *male*, **Weight =** *1.90 kg*, **Infusion Rate =** *40 ml/h*, **Prediction Time =** *1.36 s*, **Delay Time =** *0.8 s*, **Pressure Increase =** *26.73 cmH2O*

Cat8_Trial3_Normal

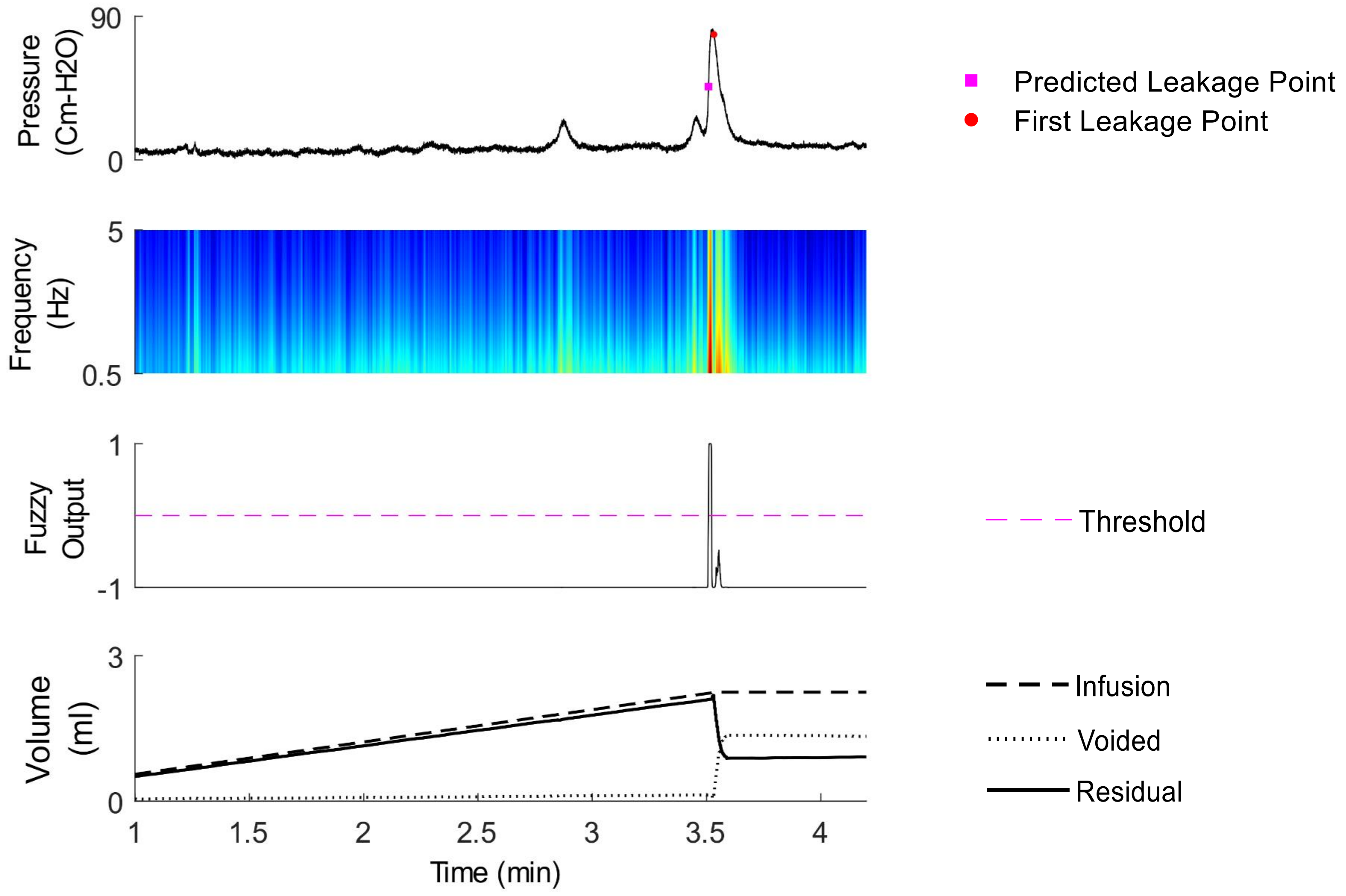

Gender = *male*, **Weight =** *1.90 kg*, **Infusion Rate =** *40 ml/h*, **Prediction Time =** *1.42 s*, **Delay Time =** *1.18 s*, **Pressure Increase =** *31.12 cmH2O*

Cat8_Trial4_Normal

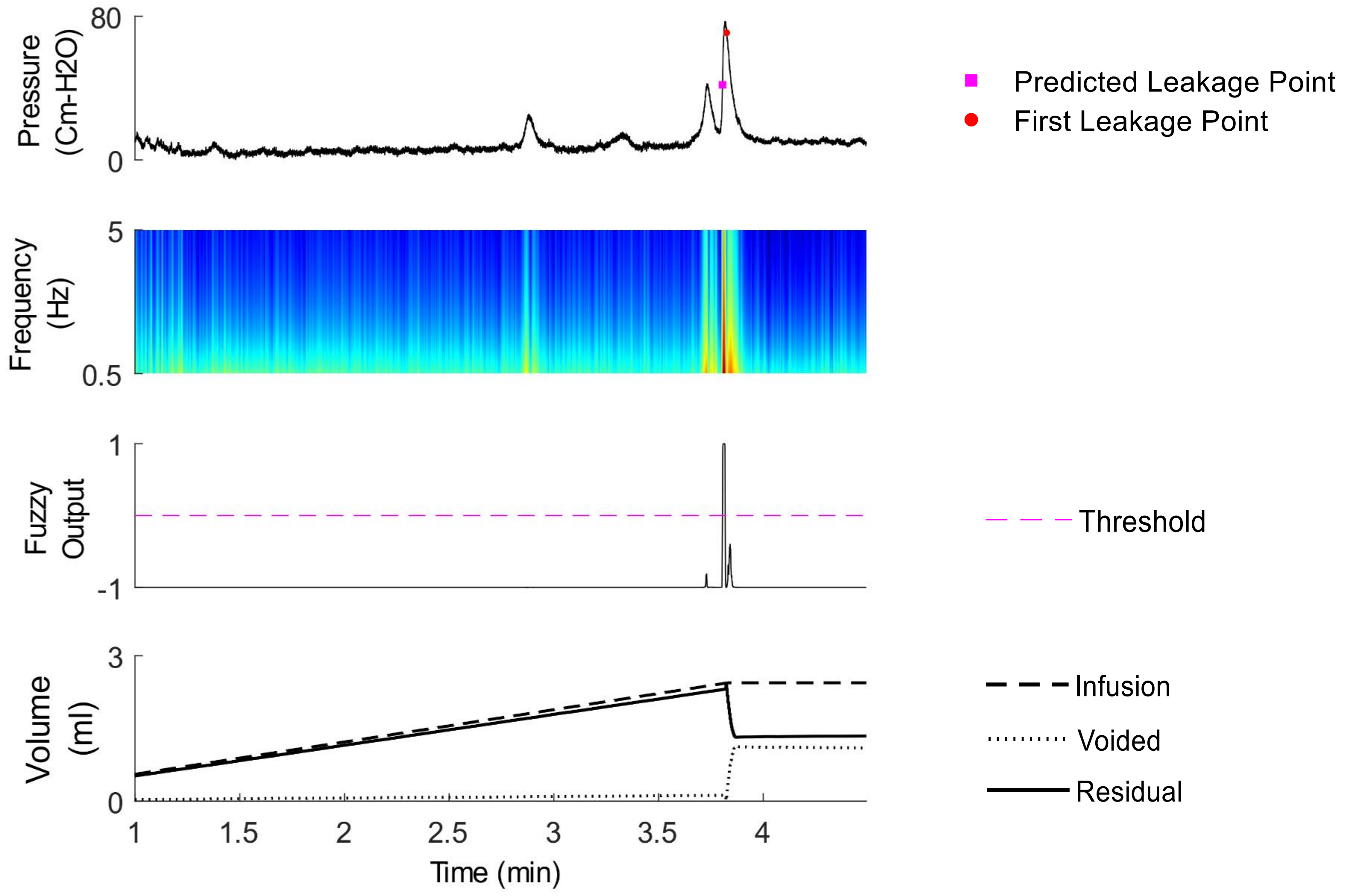

Gender = *male*, **Weight =** *1.90 kg*, **Infusion Rate =** *40 ml/h*, **Prediction Time =** *1.2 s*, **Delay Time =** *0.9 s*, **Pressure Increase =** *27.22 cmH2O*

Cat8_Trial5_Normal

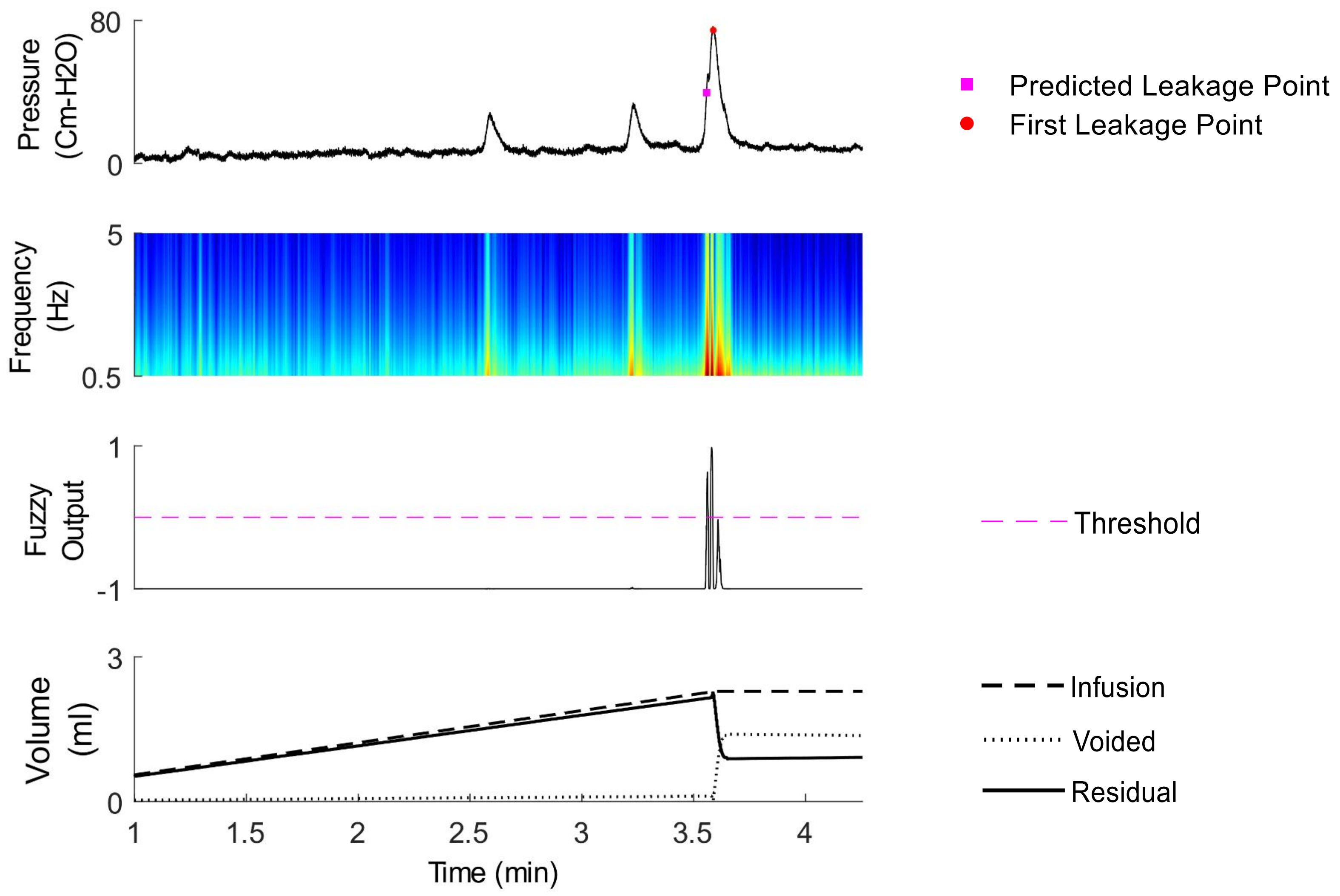

Gender = *male*, **Weight =** *1.90 kg*, **Infusion Rate =** *40 ml/h*, **Prediction Time =** *1.8 s*, **Delay Time =** *1.72 s*, **Pressure Increase =** *30.02 cmH2O*

Cat8_Trial6_Normal

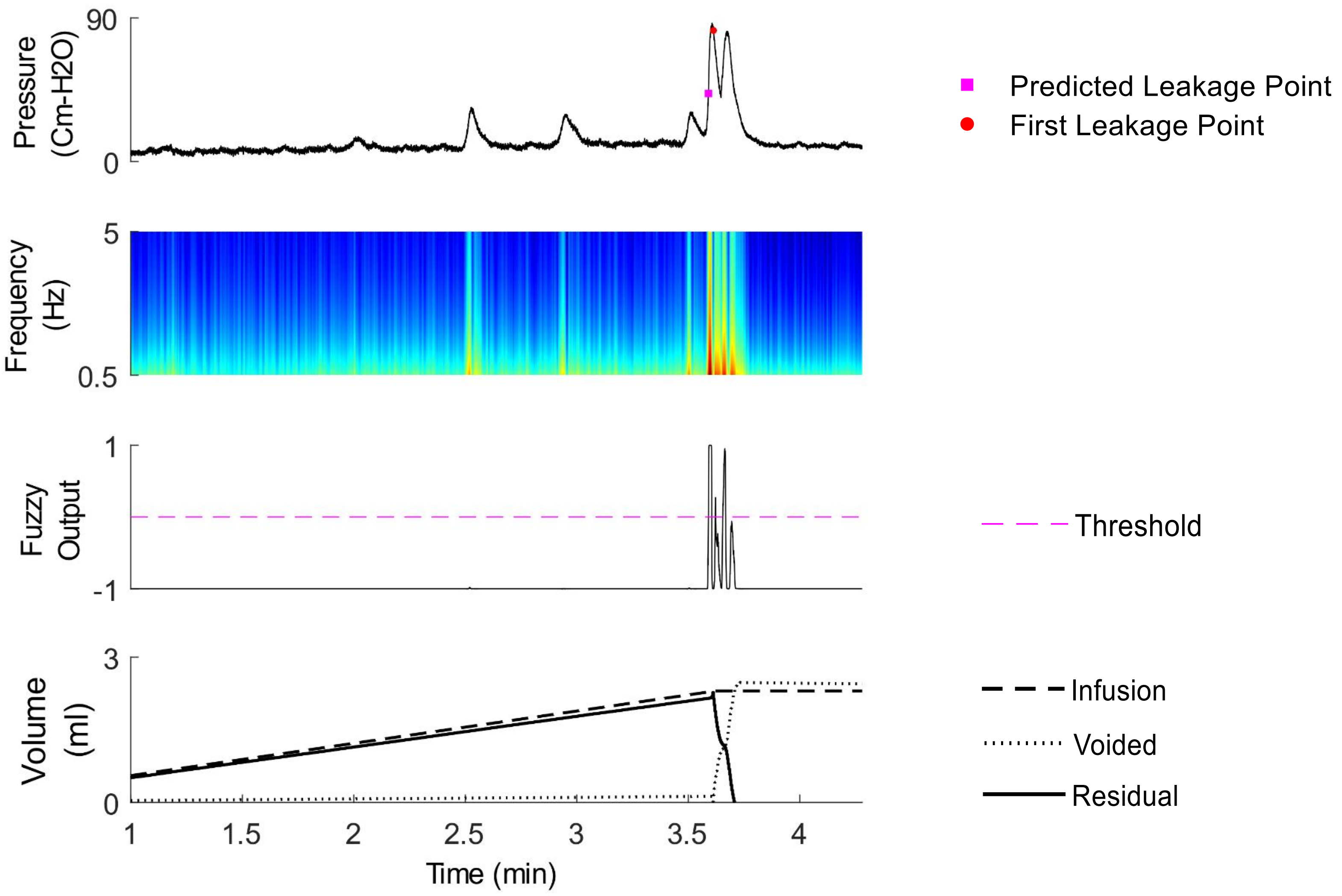

Gender = *male*, **Weight** *= 1.90 kg*, **Infusion Rate =** *40 ml/h*, **Prediction Time =** *1.38 s*, **Delay Time =** *1.18 s*, **Pressure Increase =** *25.88 cmH2O*

Cat8_Trial7_Normal

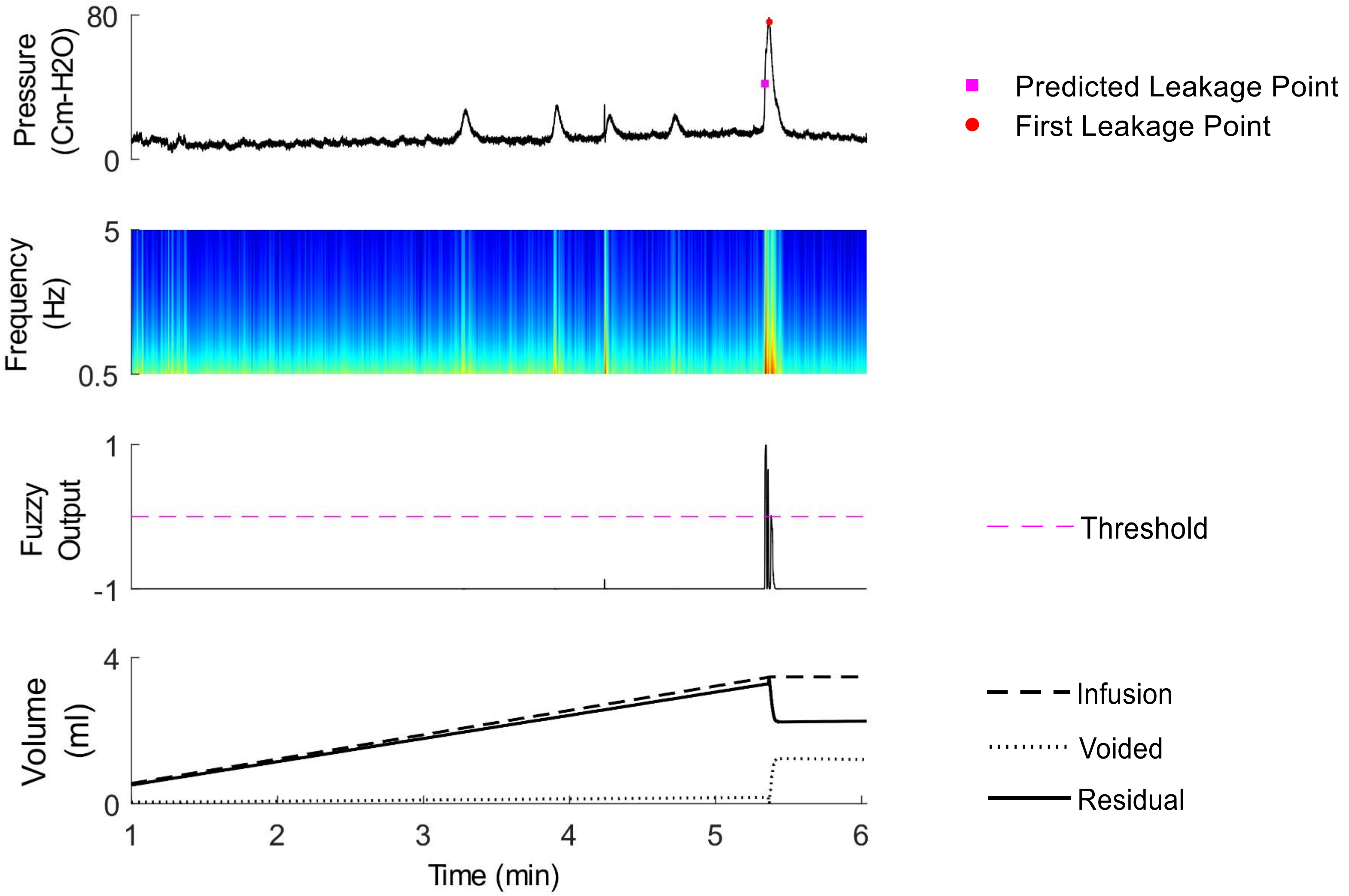

Gender = *male*, **Weight =** *1.90 kg*, **Infusion Rate =** *40 ml/h*, **Prediction Time =** *1.82 s*, **Delay Time =** *1.3 s*, **Pressure Increase =** *24.52 cmH2O*

Cat8_Trial8_Normal

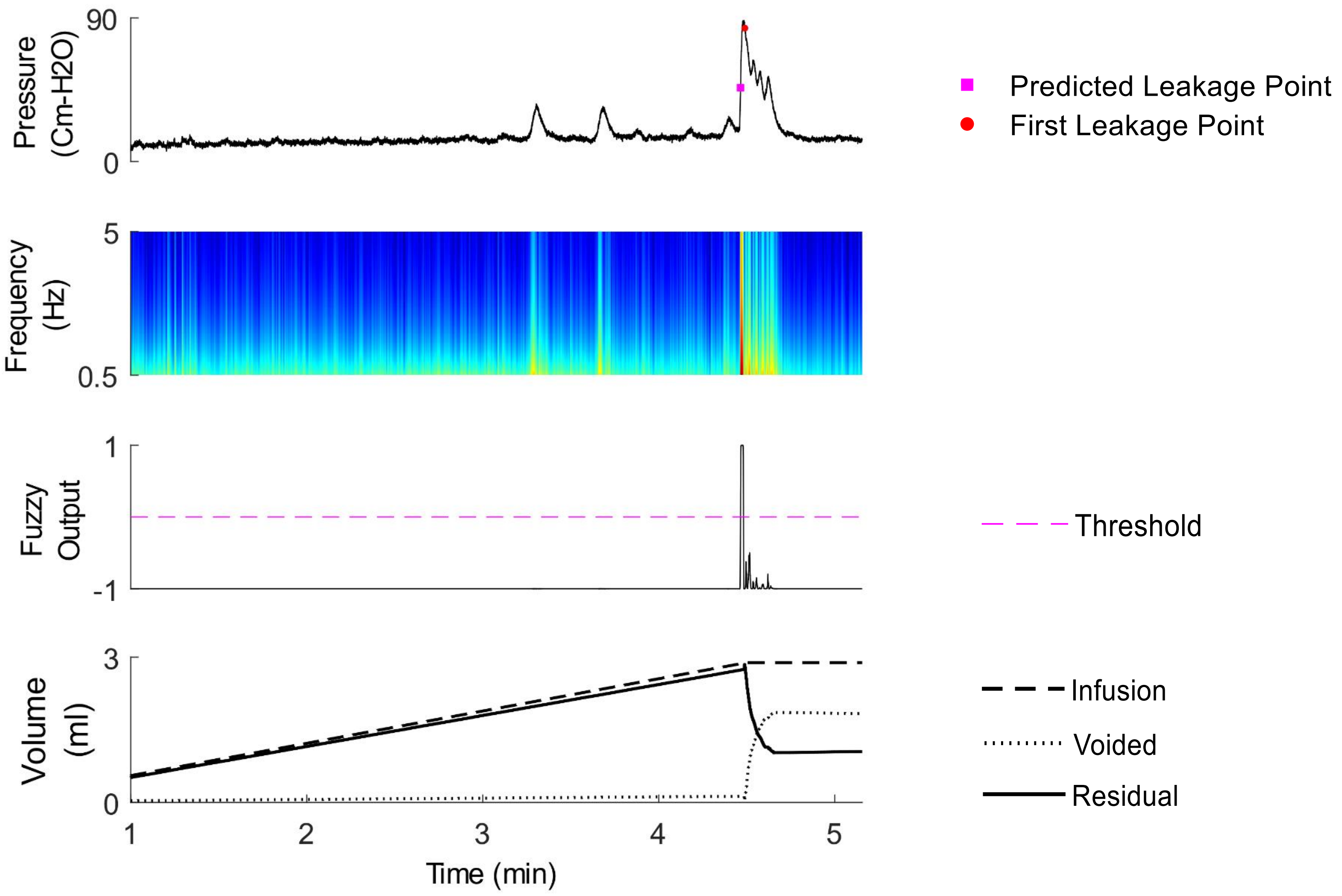

Gender = *male*, **Weight** *= 1.90 kg*, **Infusion Rate =** *40 ml/h*, **Prediction Time =** *1.52 s*, **Delay Time =** *0.46 s*, **Pressure Increase =** *22.38 cmH2O*

Cat8_Trial9_Normal

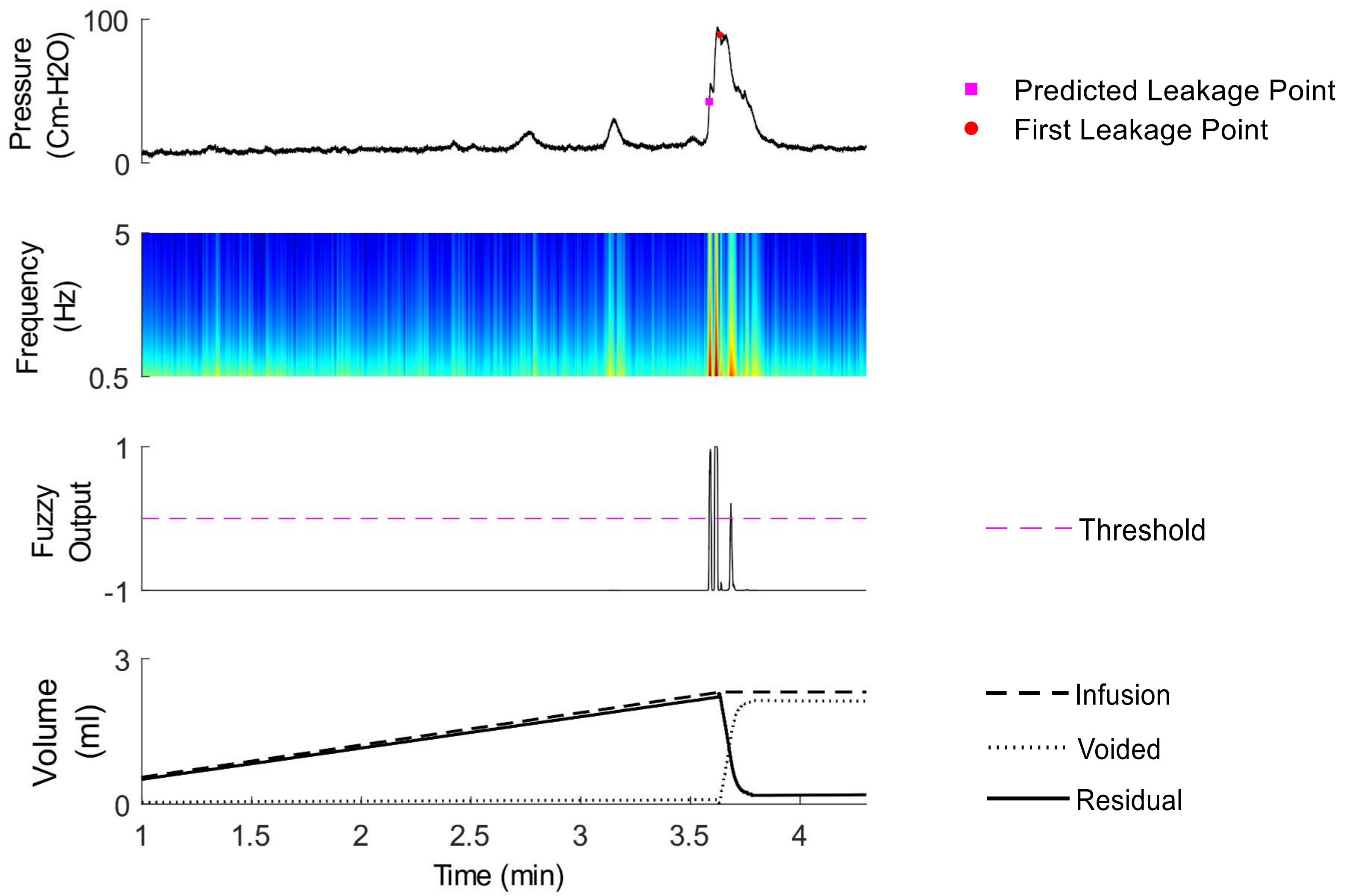

Gender = *male*, **Weight** *= 1.90 kg*, **Infusion Rate =** ***40 ml/h***, **Prediction Time =** ***2.98 s***, **Delay Time =** ***1.96 s***, **Pressure Increase =** ***26.47 cmH2O***
