# Supplementary material for: Real-time prediction of bladder urine leakage using fuzzy inference system and dual Kalman filtering in cats
Source: Sci Rep. 2024 Feb 16;14:3879. doi: 10.1038/s41598-024-53629-5 (PMC10873426; doi:10.1038/s41598-024-53629-5)
Supplement: Supplementary file 2 — Supplementary Figure S2. [file 41598_2024_53629_MOESM2_ESM.pdf]

Real-time prediction of bladder urine leakage using fuzzy inference system and dual Kalman filtering in cats

(Results of the CMG test and leakage Prediction during bladder infusion with dilute acetic acid)

Amirhossein Qasemi[1], Alireza Aminian[1], and Abbas Erfanian[1]

[1]Department of Biomedical Engineering, School of electrical engineering, Iran Neural Technology Research Center, Iran University of Science and Technology (IUST). Correspondence and requests for materials should be addressed to A.E. (email: erfanian@iust.ac.ir)

Figure S2. The results of the CMG test including measure bladder pressure, time-frequency analysis of the bladder pressure using dual Kalman filtering, infused bladder volume, voided volume, and residual bladder volume during bladder infusion with saline, as well as leakage Prediction using fuzzy logic inference system during bladder infusion with dilute acetic acid on 3 cats (20 trials in cats 6-8).

Cat6_Trial1_OAB

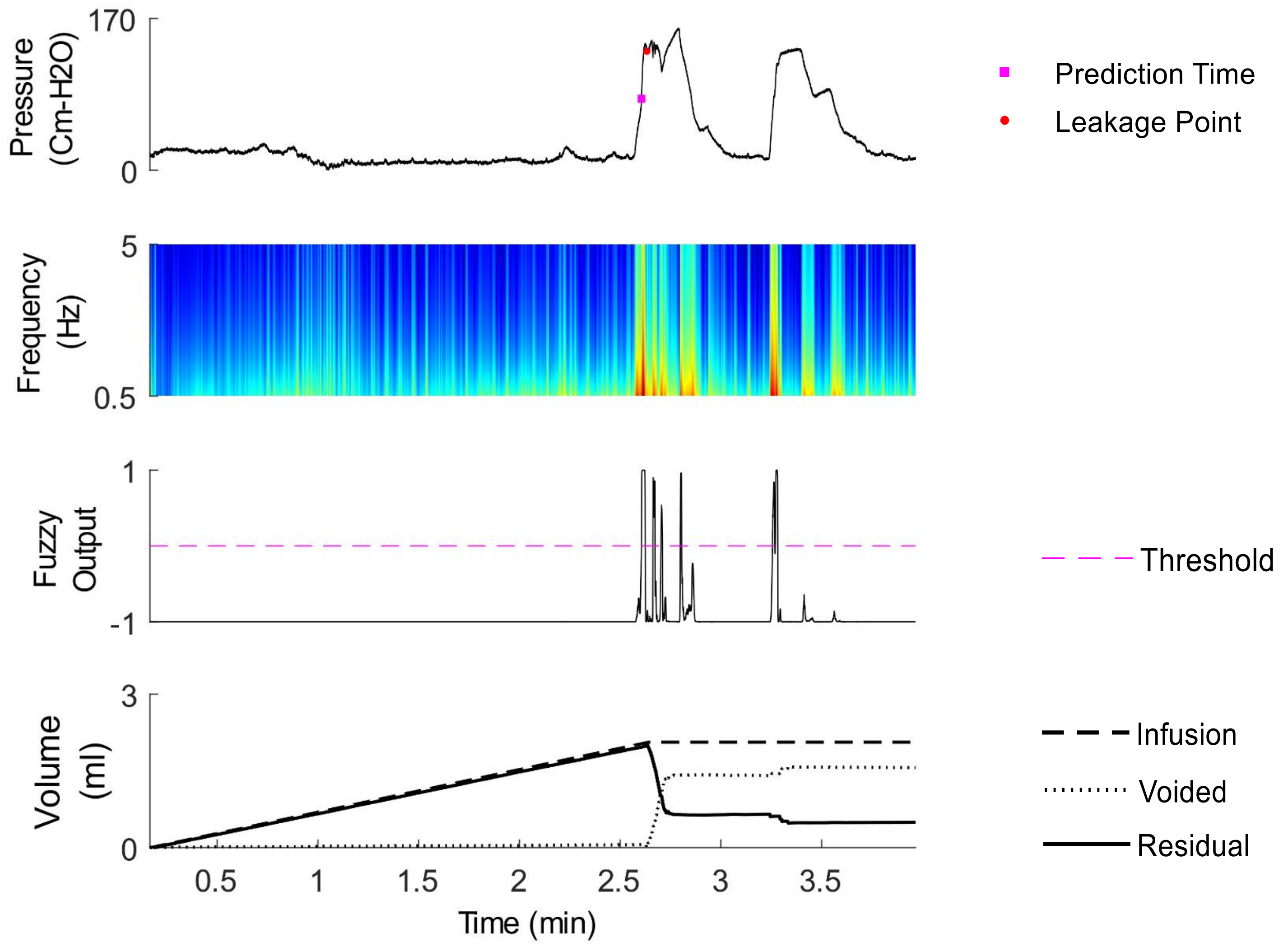

Gender = *male*, **Weight** *= 2.35 kg*, **Infusion Rate =** ***50 ml/h***, **Prediction Time =** ***1.62 s***, **Delay Time =** ***1.98 s***, **Pressure Increase =** ***62.06 cmH2O***

Cat6_Trial2_OAB

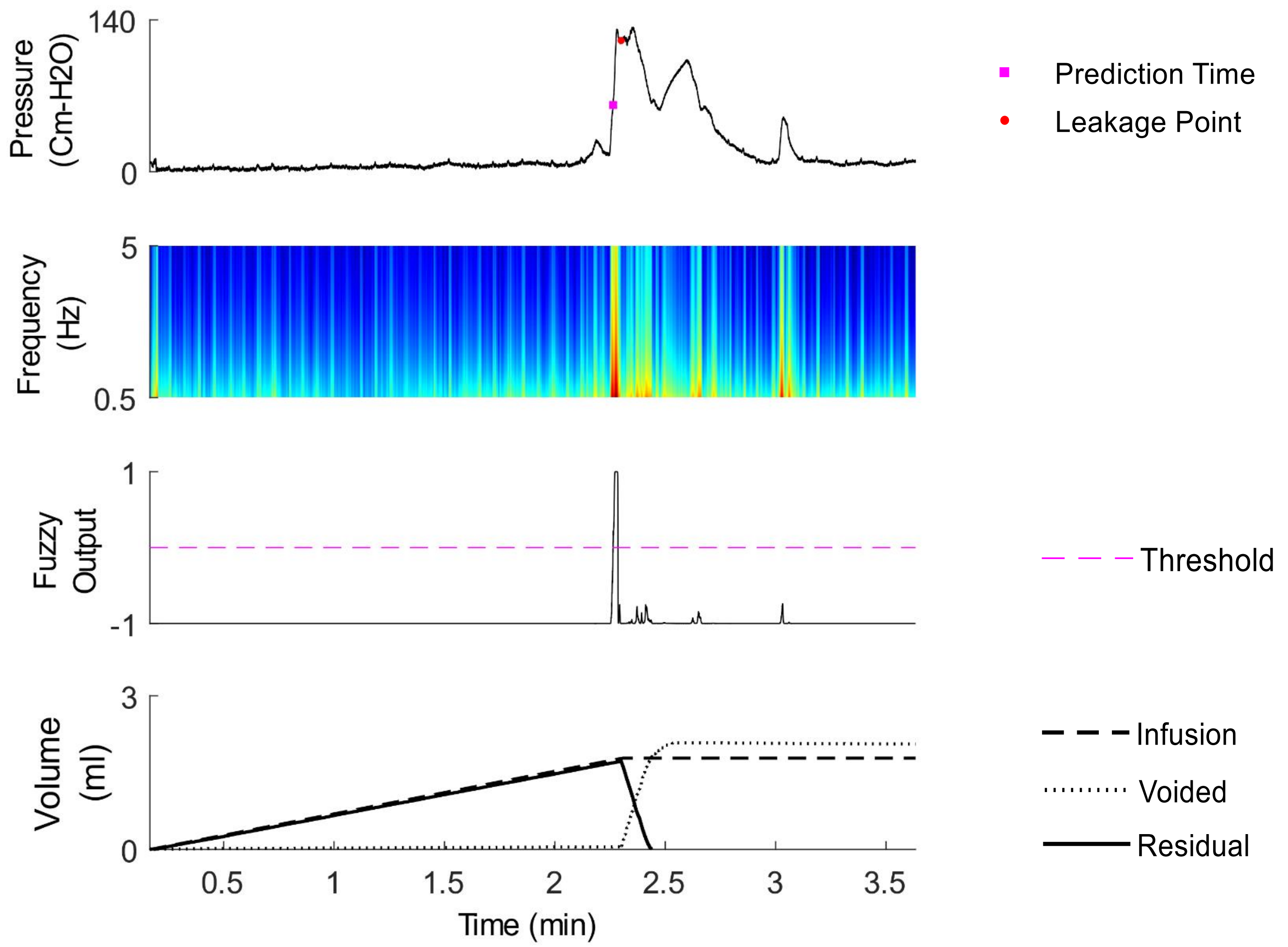

Gender = *male*, **Weight** *= 2.35 kg*, **Infusion Rate =** *50 ml/h*, **Prediction Time =** *1.62 s*, **Delay Time =** *1.88 s*, **Pressure Increase =** *64.35 cmH2O*

Cat6_Trial3_OAB

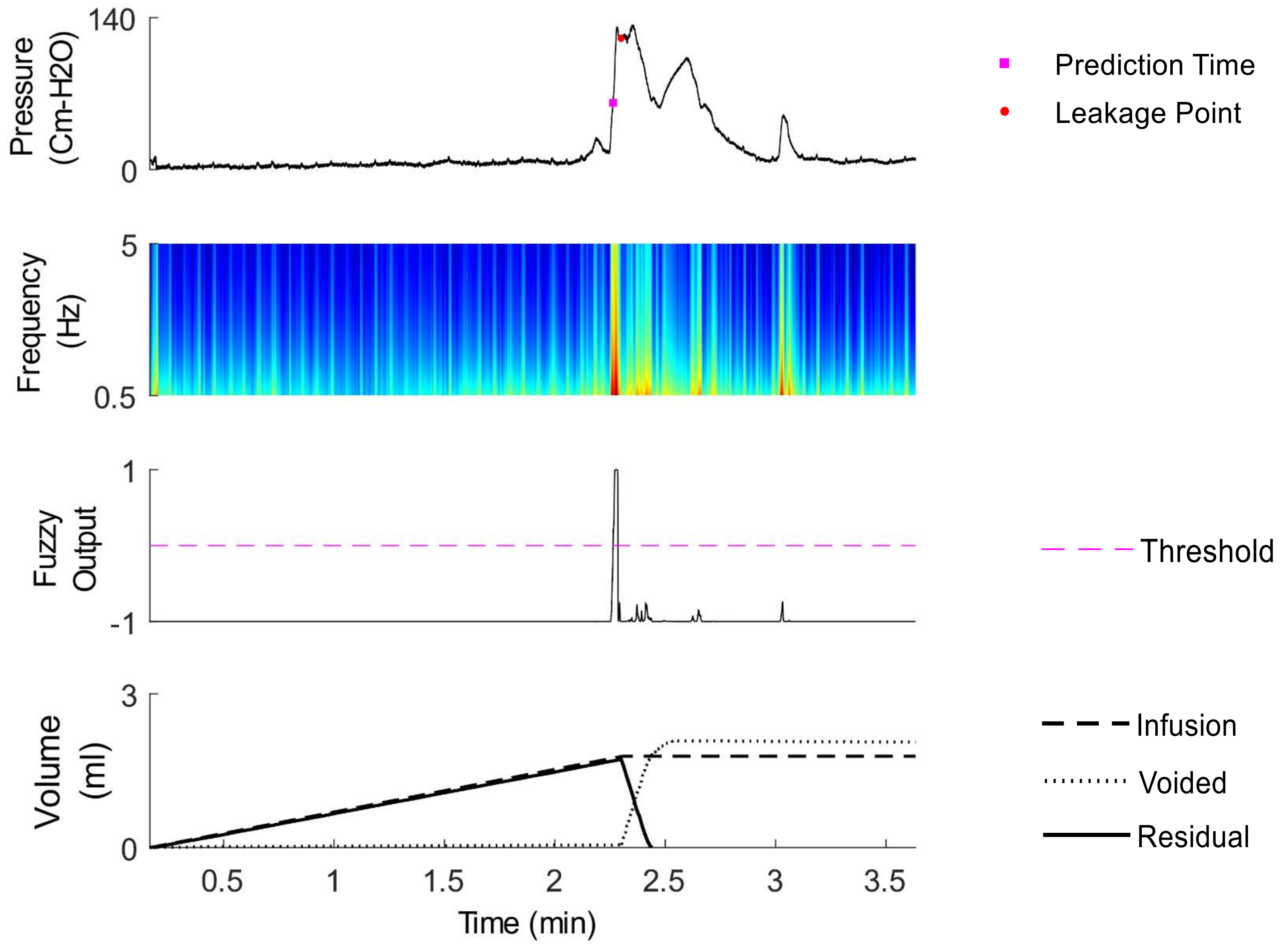

Gender = *male*, **Weight =** *2.35 kg*, **Infusion Rate =** *50 ml/h*, **Prediction Time =** *2.16 s*, **Delay Time =** *0.88 s*, **Pressure Increase =** *45.66 cmH2O*

Cat6_Trial4_OAB

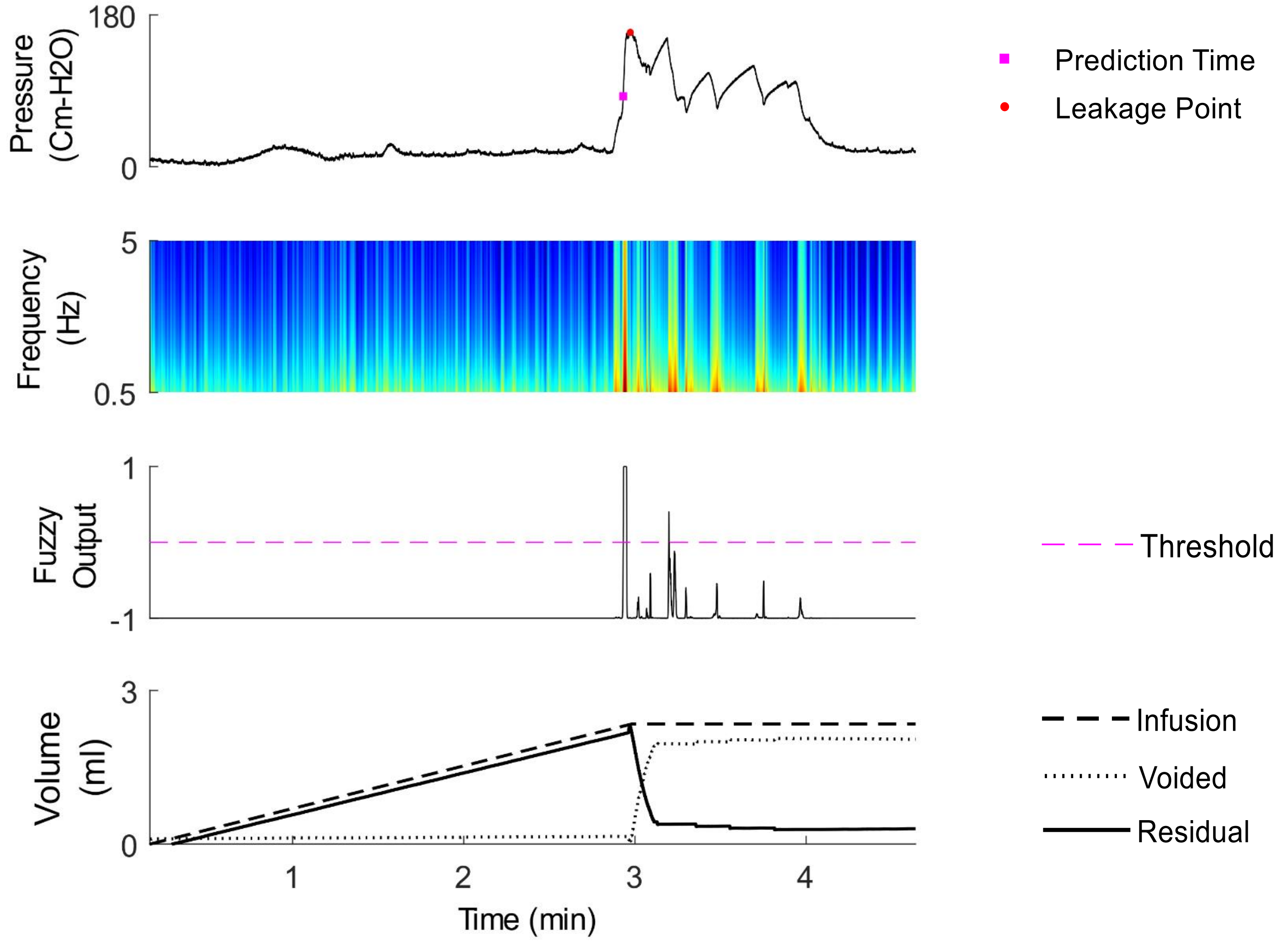

Gender = *male*, **Weight =** *2.35 kg*, **Infusion Rate =** *50 ml/h*, **Prediction Time =** *2.5 s*, **Delay Time =** *3.5 s*, **Pressure Increase =** *61.91 cmH2O*

Cat6_Trial5_OAB

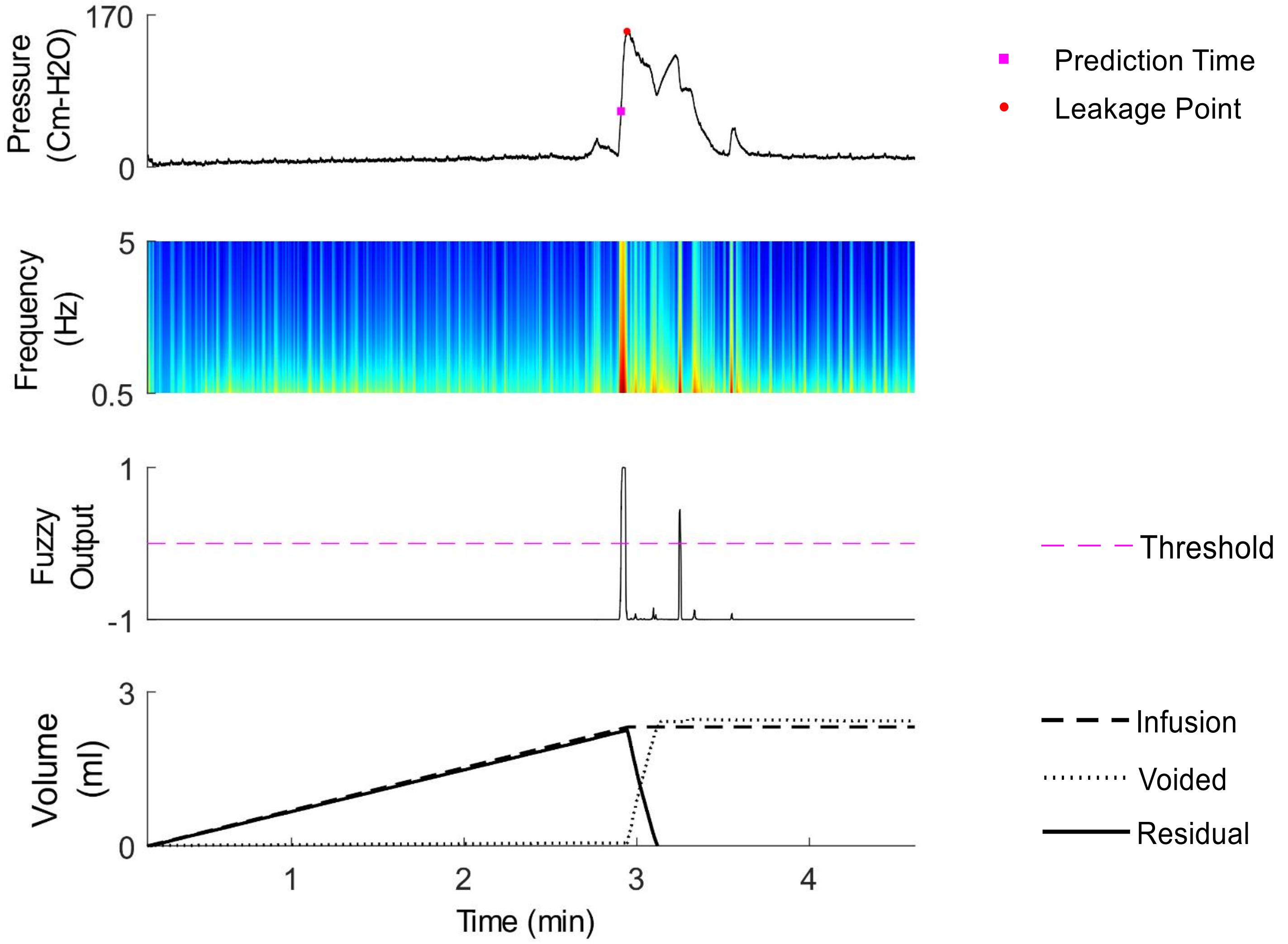

Gender = *male*, **Weight =** *2.35 kg*, **Infusion Rate =** *50 ml/h*, **Prediction Time =** *2.1 s*, **Delay Time =** *1.04 s*, **Pressure Increase =** *51.85 cmH2O*

Cat6_Trial6_OAB

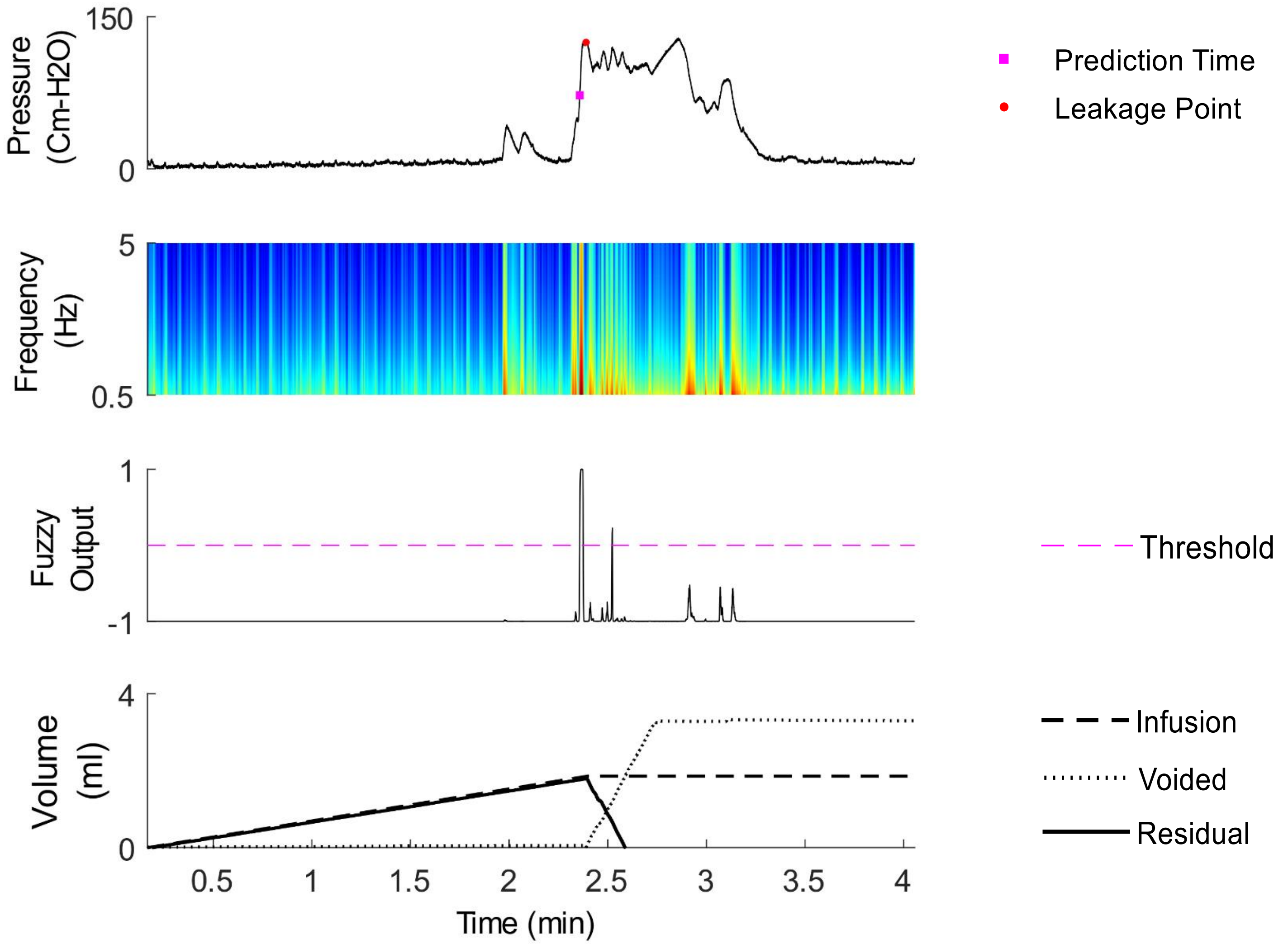

Gender = *male*, **Weight =** *2.35 kg*, **Infusion Rate =** *50 ml/h*, **Prediction Time =** *1.9 s*, **Delay Time =** *2.62 s*, **Pressure Increase =** *61.4 cmH2O*

Cat6_Trial7_OAB

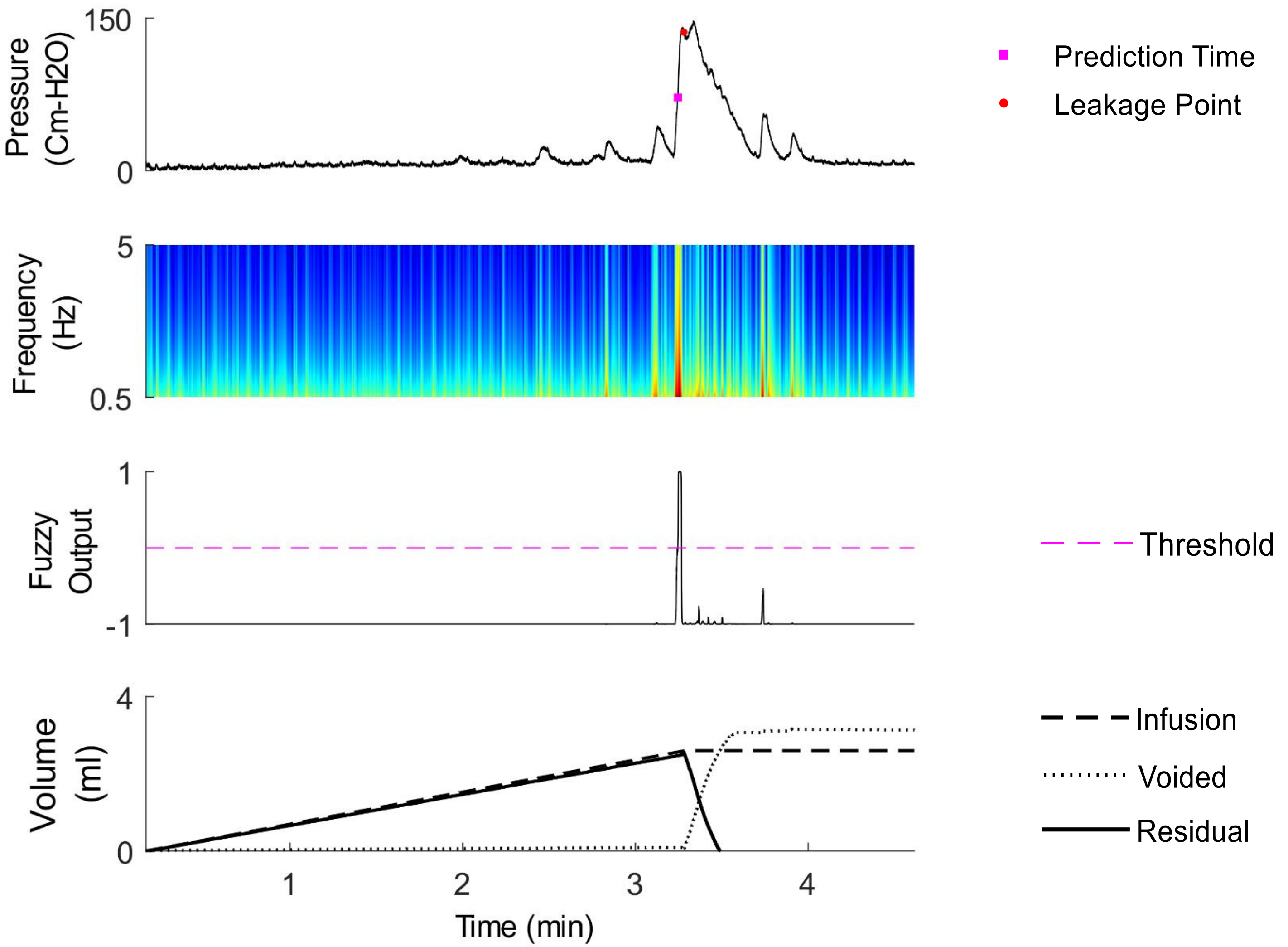

Gender = *male*, **Weight =** *2.35 kg*, **Infusion Rate =** *50 ml/h*, **Prediction Time =** *2.1 s*, **Delay Time =** *1.22 s*, **Pressure Increase =** *54.65 cmH2O*

Cat7_Trial1_OAB

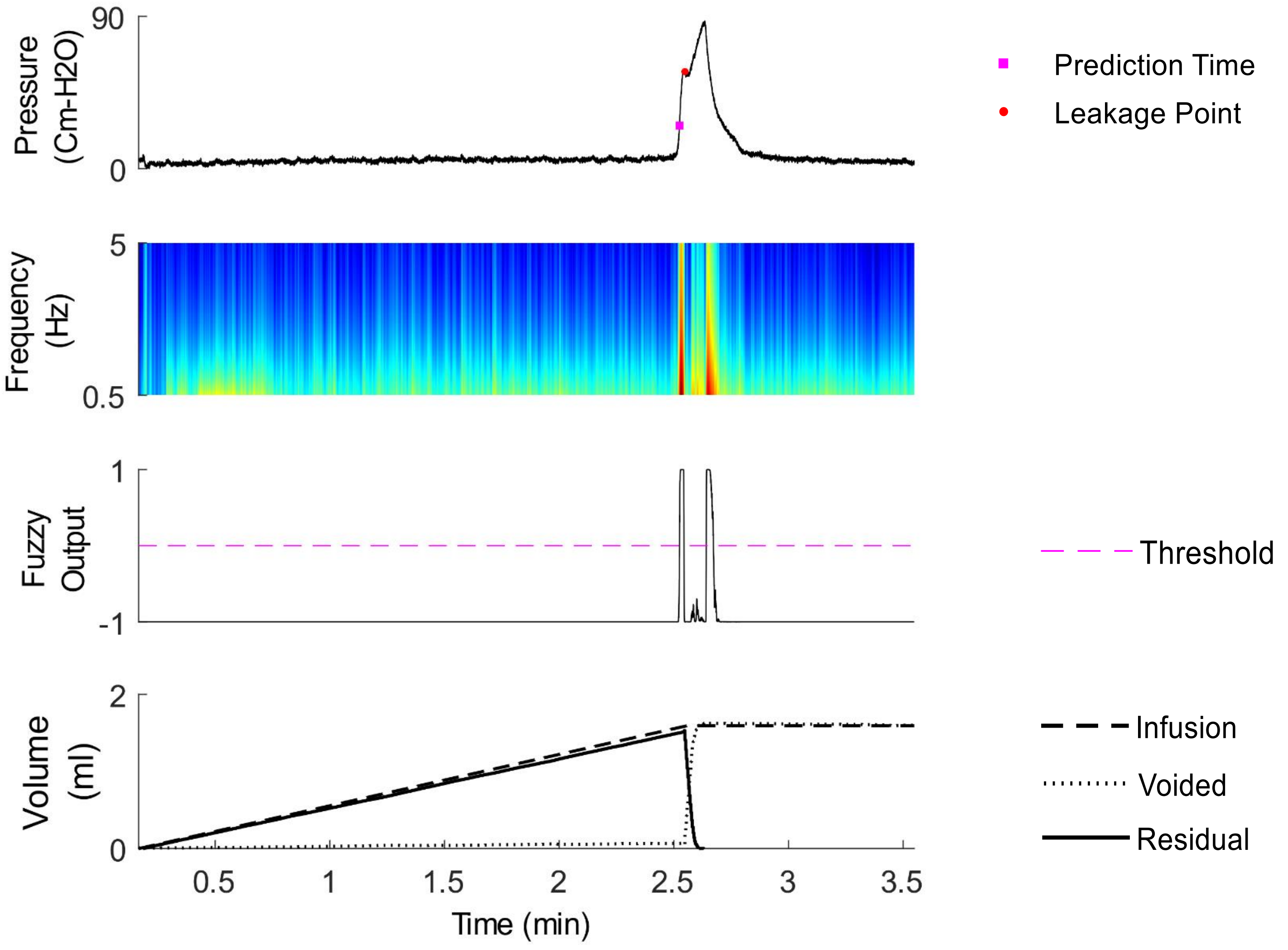

Gender = *male*, **Weight =** *2.93 kg*, **Infusion Rate =** *40 ml/h*, **Prediction Time =** *1.4 s*, **Delay Time =** *0.7 s*, **Pressure Increase =** *16.7 cmH2O*

Cat7_Trial2_OAB

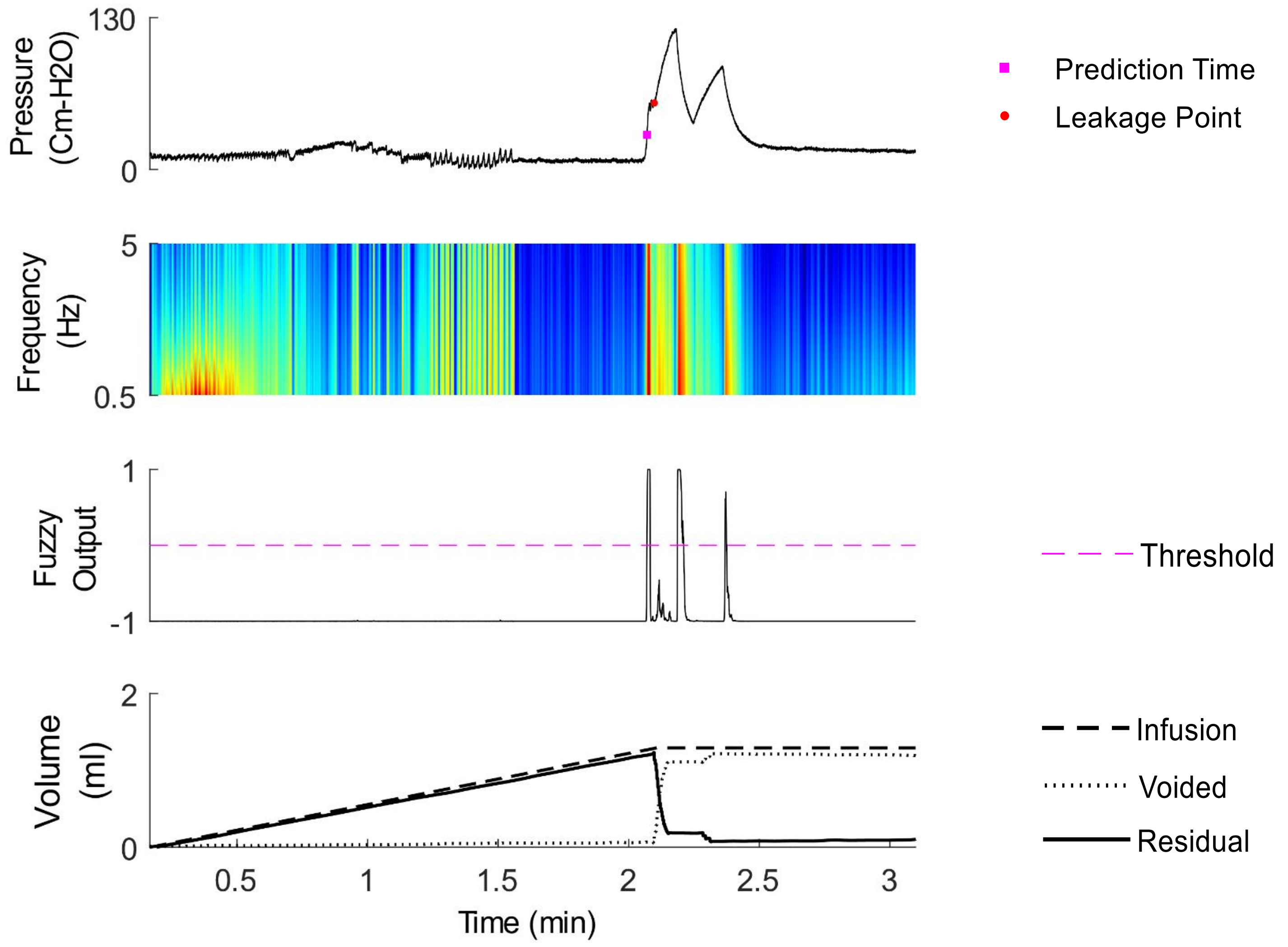

Gender = *male*, **Weight =** *2.93 kg*, **Infusion Rate =** *40 ml/h*, **Prediction Time =** *1.7 s*, **Delay Time =** *0.42 s*, **Pressure Increase =** *17.37 cmH2O*

Cat7_Trial3_OAB

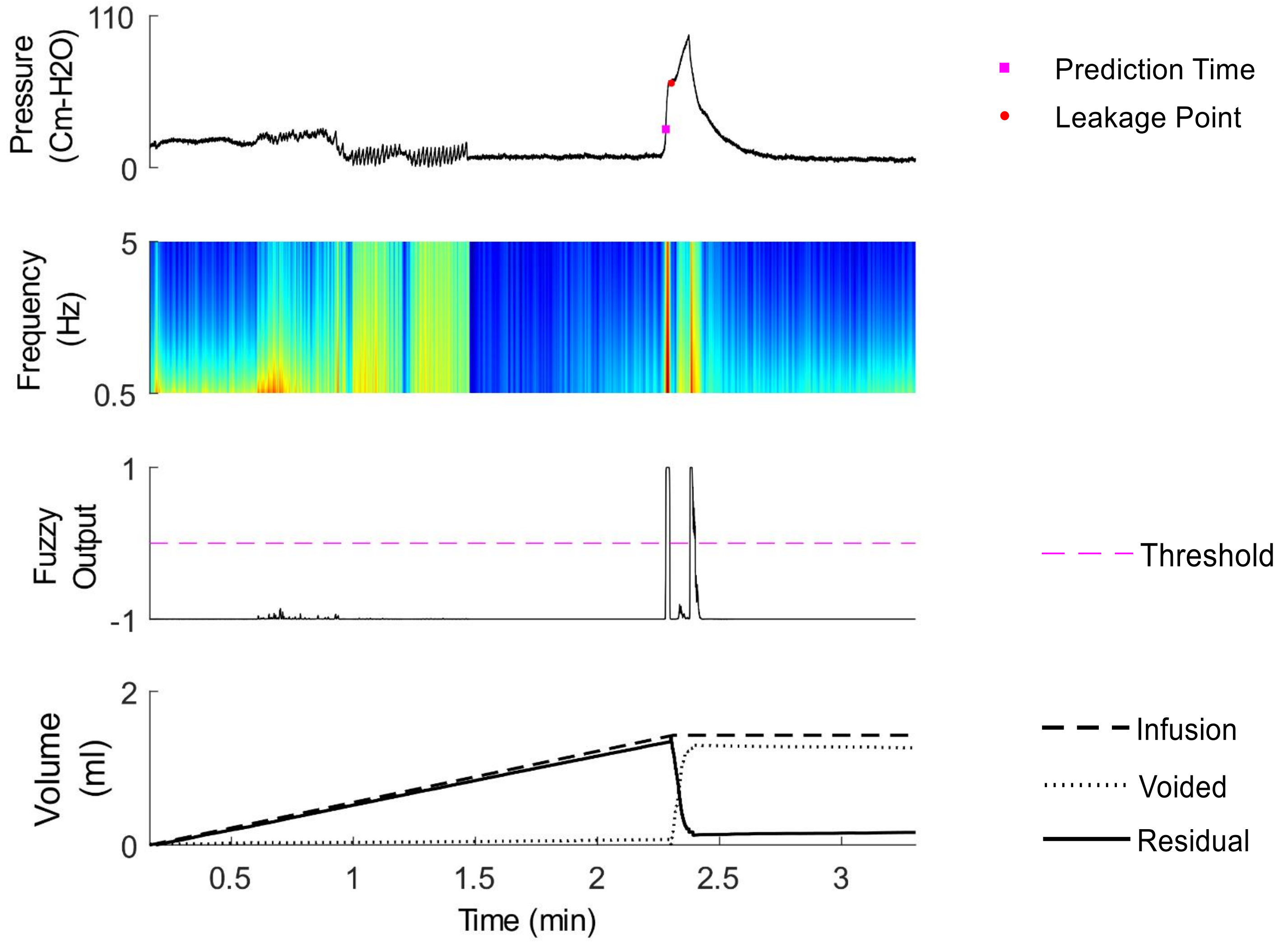

Gender = *male*, **Weight =** *2.93 kg*, **Infusion Rate =** *40 ml/h*, **Prediction Time =** *1.38 s*, **Delay Time =** *0.6 s*, **Pressure Increase =** *17.11 cmH2O*

Cat7_Trial4_OAB

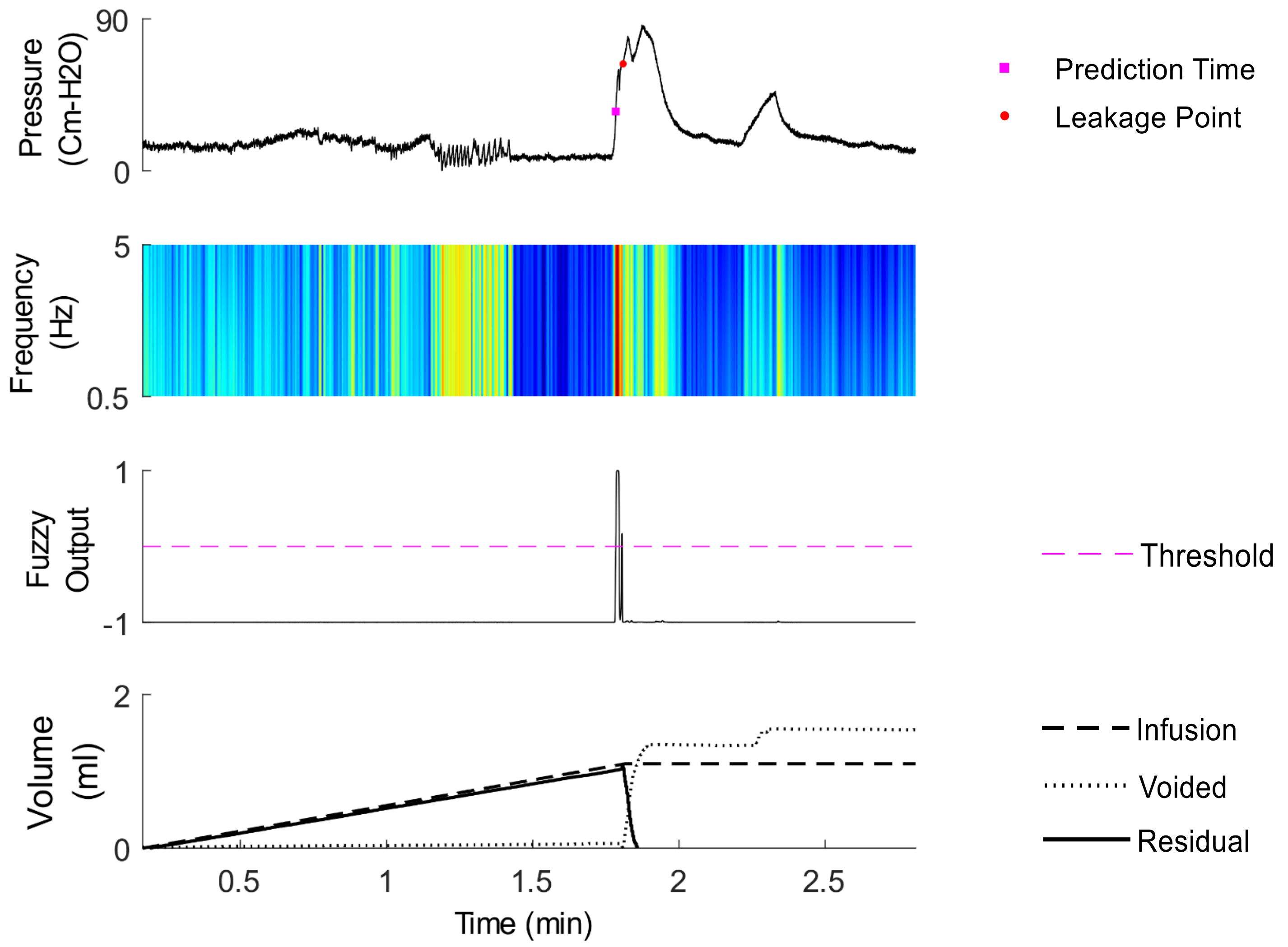

Gender = *male*, **Weight =** *2.93 kg*, **Infusion Rate =** *40 ml/h*, **Prediction Time =** *1.5 s*, **Delay Time =** *0.5 s*, **Pressure Increase =** *24.08 cmH2O*

Cat7_Trial5_OAB

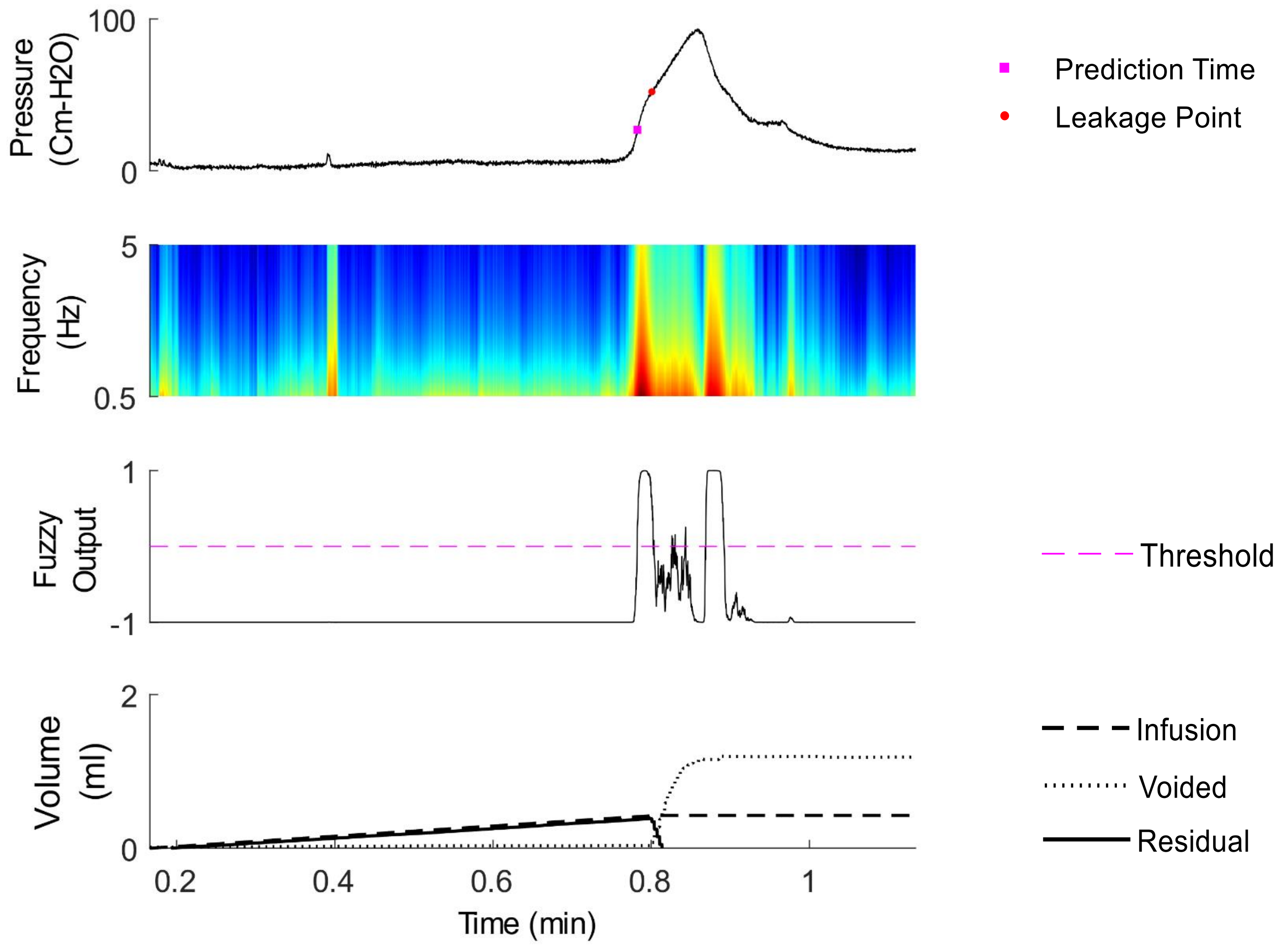

Gender = *male*, **Weight =** *2.93 kg*, **Infusion Rate =** *40 ml/h*, **Prediction Time =** *1.04 s*, **Delay Time =** *0.7 s*, **Pressure Increase =** *17.22 cmH2O*

Cat7_Trial6_OAB

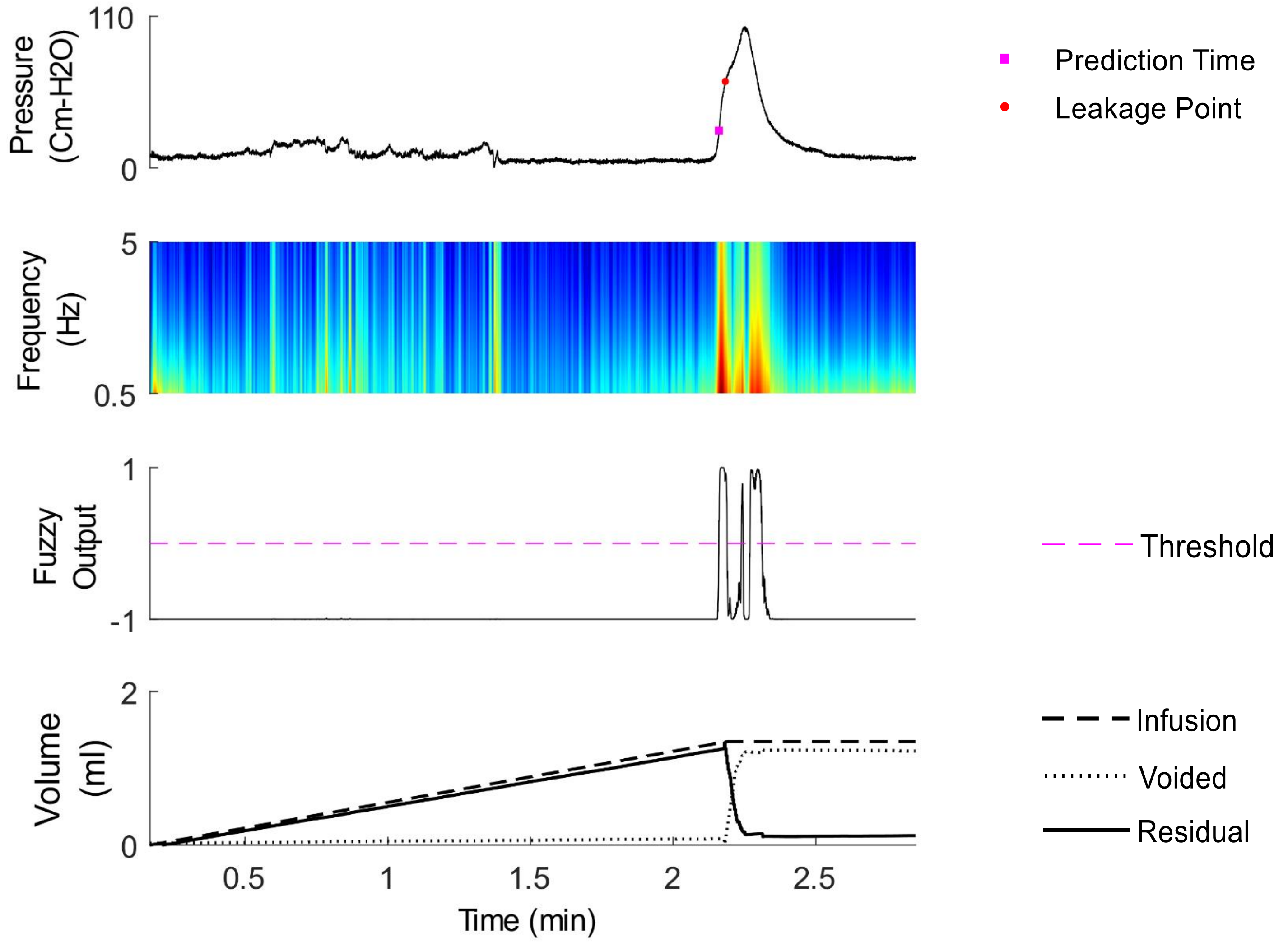

Gender = *male*, **Weight** *= 2.93 kg*, **Infusion Rate =** ***40 ml/h***, **Prediction Time =** ***1.34 s***, **Delay Time =** ***0.68 s***, **Pressure Increase =** ***17.46 cmH2O***

Cat7_Trial7_OAB

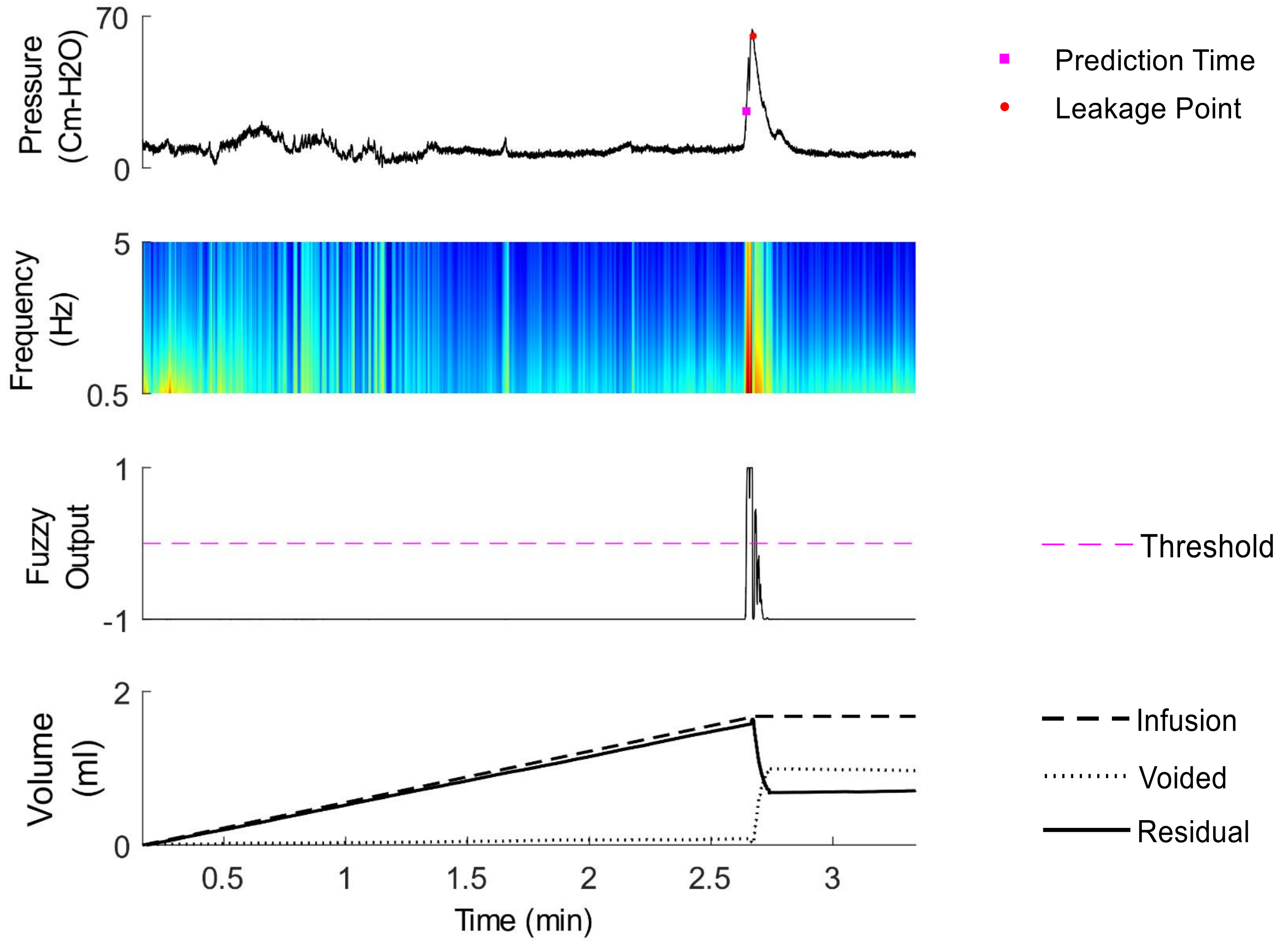

Gender = *male*, **Weight** *= 2.93 kg*, **Infusion Rate =** *40 ml/h*, **Prediction Time =** *1.66 s*, **Delay Time =** *0.66 s*, **Pressure Increase =** *15.81 cmH2O*

Cat8_Trial1_OAB

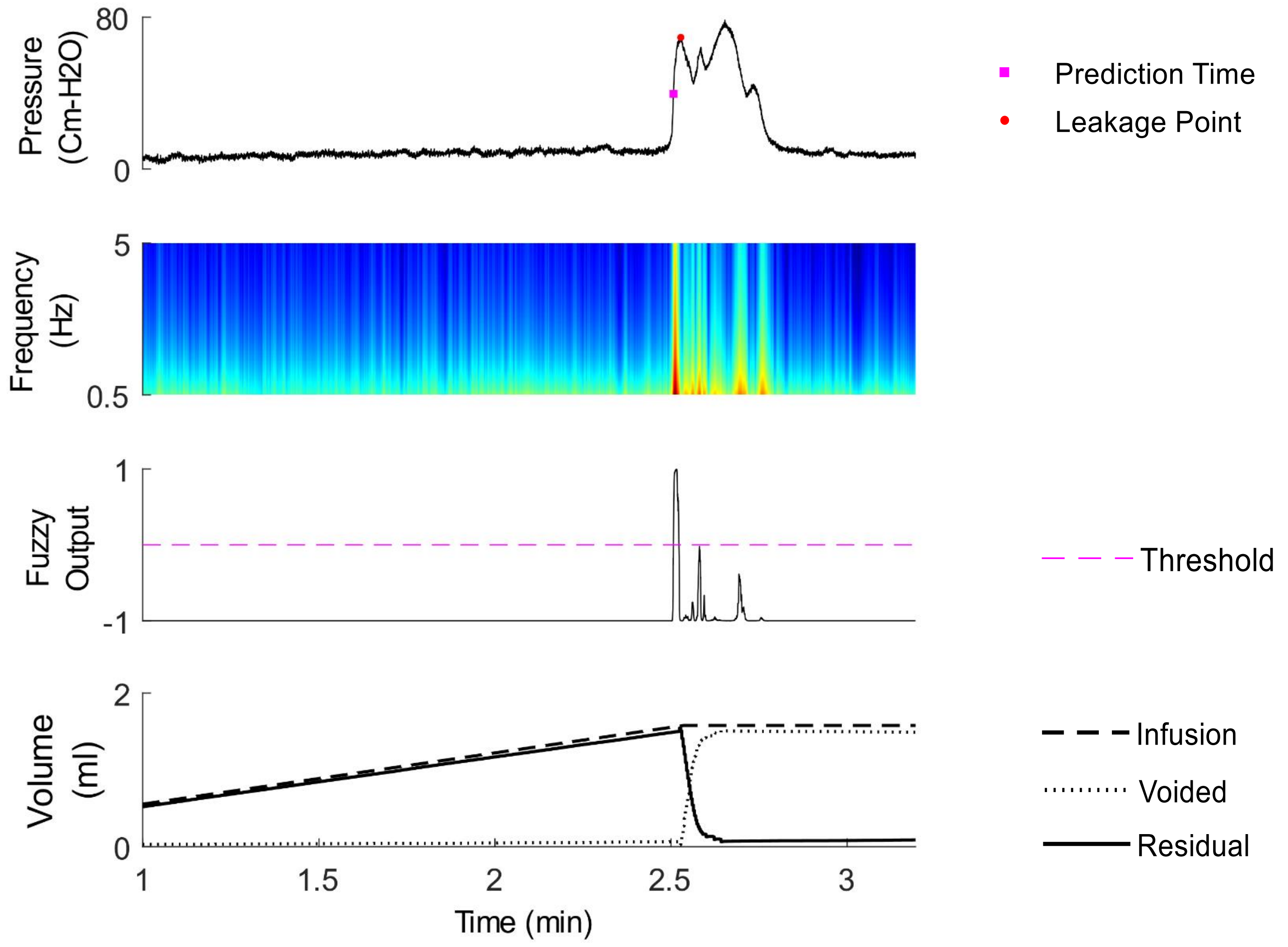

Gender = *male*, **Weight =** *1.90 kg*, **Infusion Rate =** *40 ml/h*, **Prediction Time =** *1.28 s*, **Delay Time =** *0.5 s*, **Pressure Increase =** *22.97 cmH2O*

Cat8_Trial2_OAB

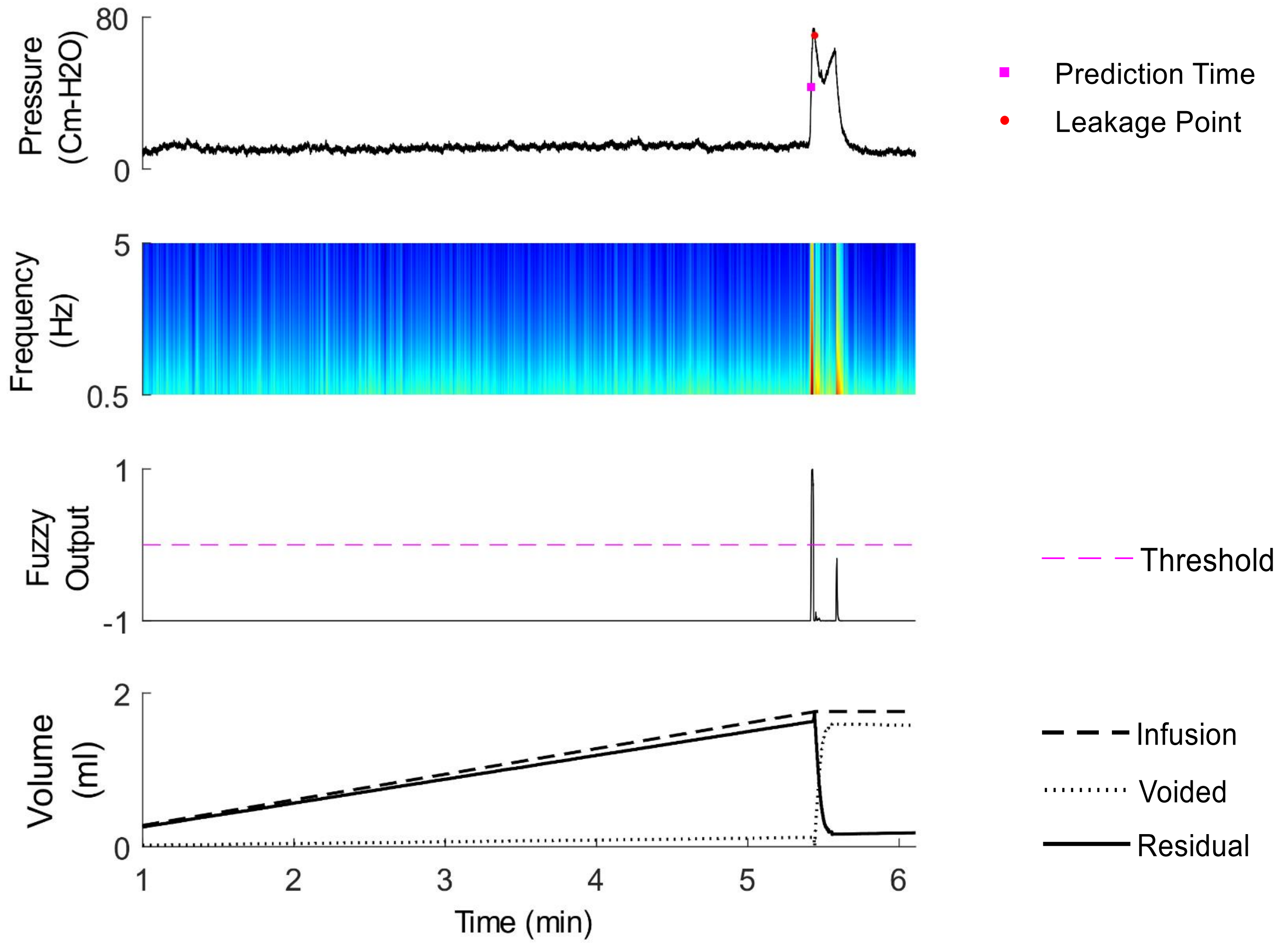

Gender = *male*, **Weight =** *1.90 kg*, **Infusion Rate =** *20 ml/h*, **Prediction Time =** *1.48 s*, **Delay Time =** *0.82 s*, **Pressure Increase =** *24.07 cmH2O*

Cat8_Trial3_OAB

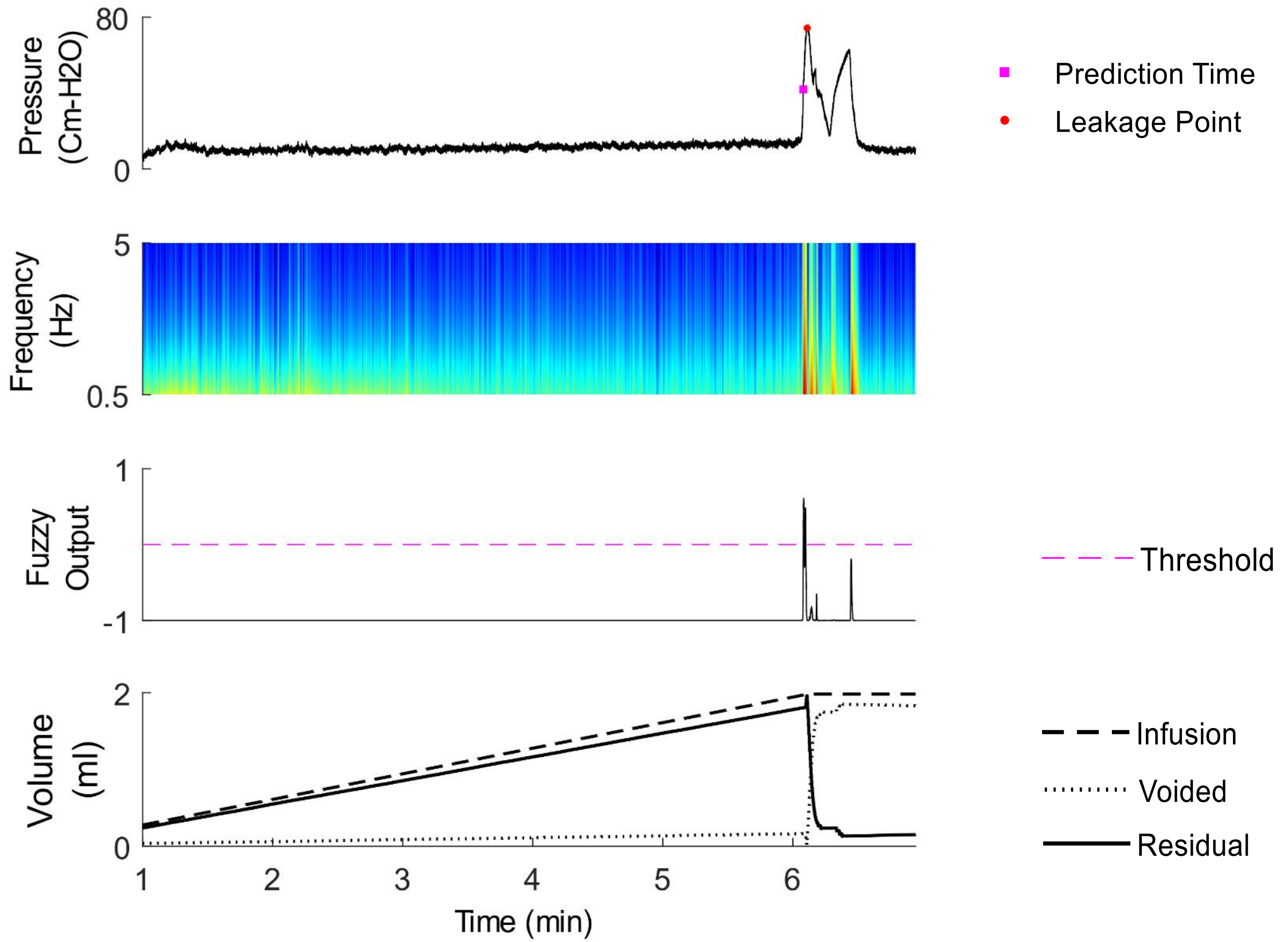

Gender = *male*, **Weight =** *1.90 kg*, **Infusion Rate =** *20 ml/h*, **Prediction Time =** *1.84 s*, **Delay Time =** *0.96 s*, **Pressure Increase =** *24.46 cmH2O*

Cat7_Trial4_OAB

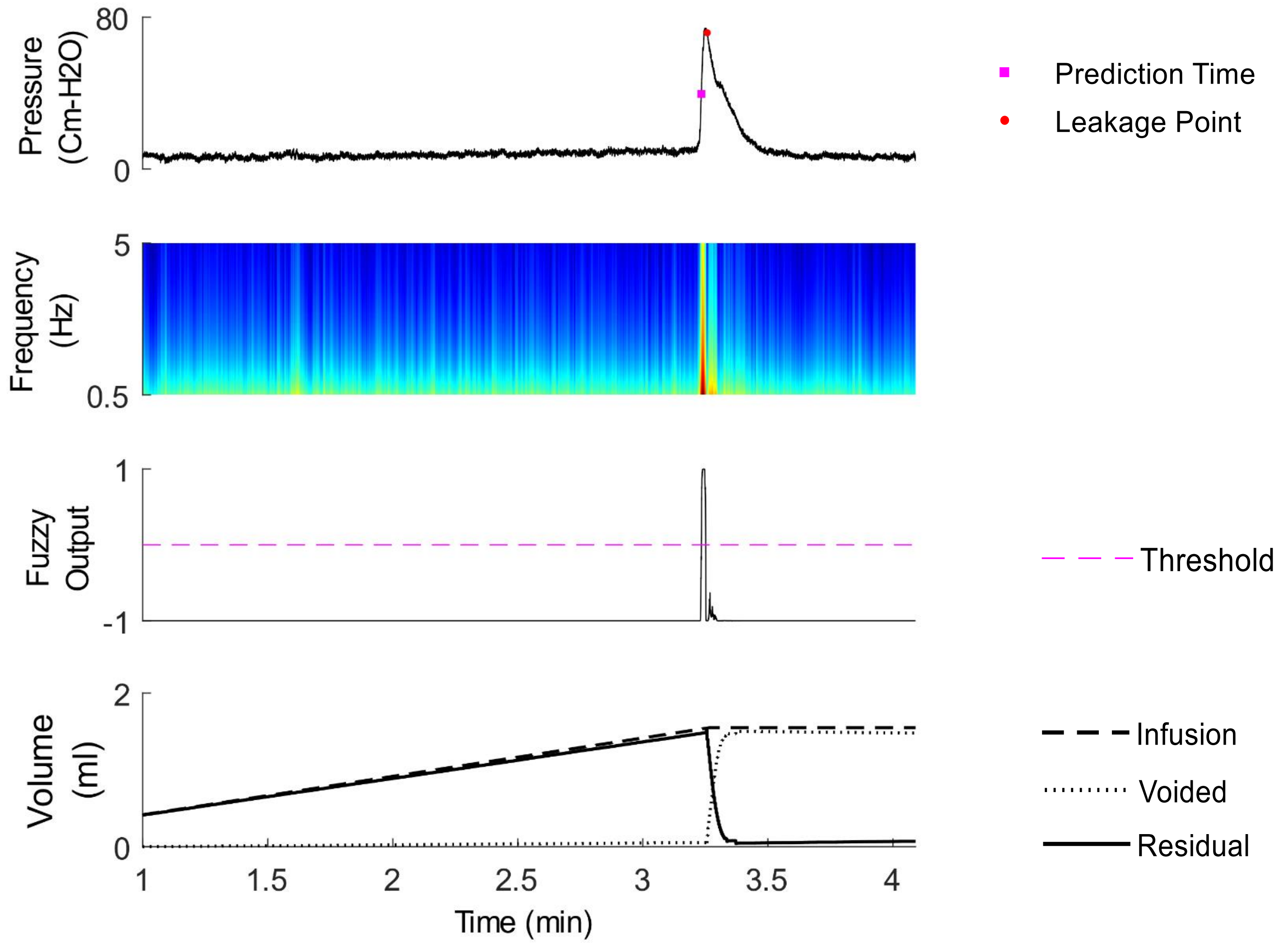

Gender = *male*, **Weight =** *1.90 kg*, **Infusion Rate =** *30 ml/h*, **Prediction Time =** *1.44 s*, **Delay Time =** *1.16 s*, **Pressure Increase =** *27.9 cmH2O*

Cat8_Trial5_OAB

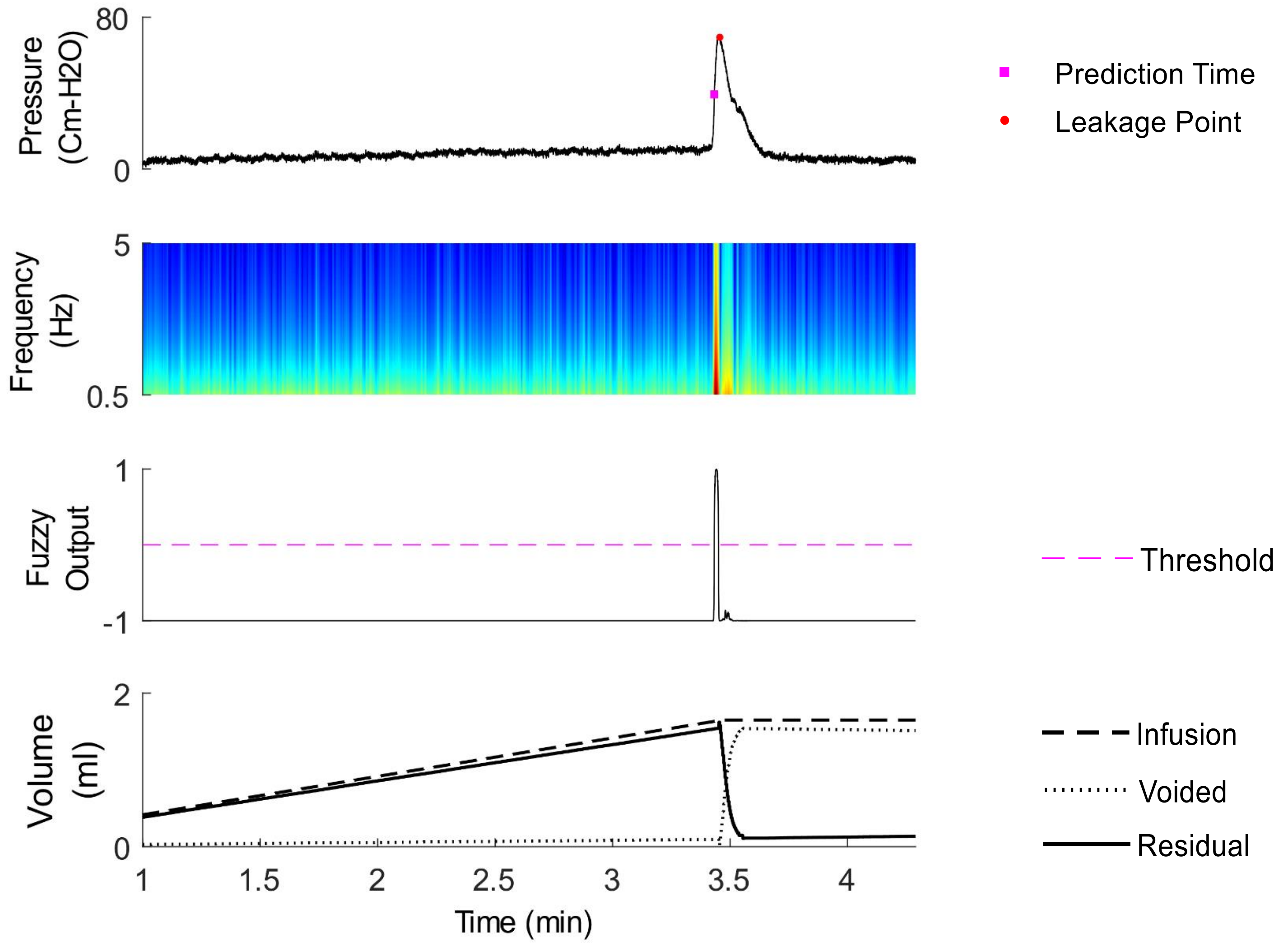

Gender = *male*, **Weight =** *1.90 kg*, **Infusion Rate =** *30 ml/h*, **Prediction Time =** *1.4 s*, **Delay Time =** *0.68 s*, **Pressure Increase =** *26.97 cmH2O*

Cat8_Trial6_OAB

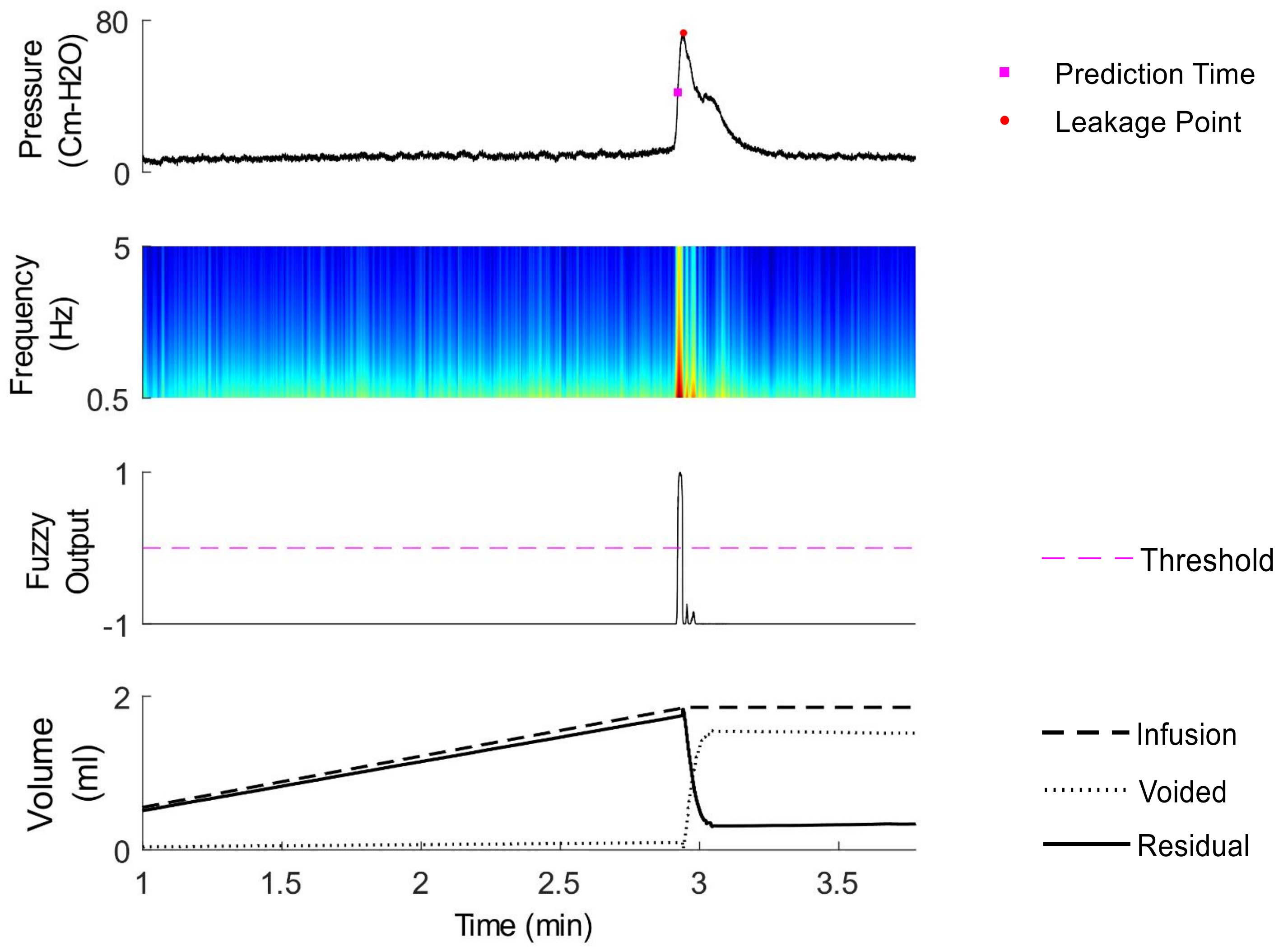

Gender = *male*, **Weight =** *1.90 kg*, **Infusion Rate =** *40 ml/h*, **Prediction Time =** *1.3 s*, **Delay Time =** *1.06 s*, **Pressure Increase =** *26.18 cmH2O*
